# Robust intensification of projected regional precipitation extremes over Africa

**DOI:** 10.1038/s41467-026-73246-2

**Published:** 2026-05-16

**Authors:** Akintomide A. Akinsanola, Thierry N. Taguela, Vishal Bobde

**Affiliations:** Department of Earth and Environmental Sciences, University of Illinois Chicago, Chicago, IL USA

**Keywords:** Climate change, Climate-change impacts

## Abstract

Extreme rainfall is increasing under climate warming, but its future patterns across Africa remain highly uncertain. Using Coupled Model Intercomparison Project Phase 6 (CMIP6) simulations, we assess projected changes in annual maximum one-day precipitation (Rx1day) and rare precipitation extremes across African subregions under both SSP2-4.5 and SSP5-8.5 scenarios. Our results show robust intensification of Rx1day by the late twenty-first century, with increases of ~5–10 mm day⁻¹ (15–23 mm day⁻¹) under SSP2-4.5 (SSP5-8.5), largest in convectively dominated equatorial regions such as West, Central, and Northeast Africa. Rare events that historically occurred once every 50 (100) years are projected to recur every ~5–10 ( ~ 6–14) years, with recurrence intervals as short as 2–3 years in equatorial regions under SSP5-8.5. This intensification is driven primarily by thermodynamic moistening associated with radiation-induced warming, while diabatic heating–driven dynamic changes modulate regional responses and account for much of the intermodel spread. A hierarchical emergent-constraint framework based on observed historical global mean surface temperature trends moderates mean Rx1day intensification by ~11–35% without altering its sign. Constrained and unconstrained projections indicate substantial continent-wide increases in population and gross domestic product exposure.

## Abstract (web summary)

Extreme rainfall will intensify across Africa under future warming, with the largest increases expected in equatorial regions. This intensification is driven primarily by increased atmospheric moisture associated with radiation-induced warming.

## Introduction

Extreme precipitation events, characterized by unusually intense precipitation occurring over short durations, have increased in both frequency and magnitude in many regions across the globe, posing major challenges to societies, ecosystems, and infrastructure^1–8^. These events are often linked to flooding, landslides, infrastructure failure, disruptions to agriculture, and loss of life, leading to widespread socioeconomic losses^9–13^. Between 2000 and 2019, floods accounted for approximately 44% of all weather-related disasters, affecting 1.6 billion people worldwide^14^. Combined with storm-related impacts, flooding caused global losses exceeding US$2 trillion, with low-income countries suffering the largest relative GDP losses^14^. These disproportionate impacts are especially concerning for Africa, which, despite its relatively low contribution to global emissions, is projected to experience some of the most rapid and pronounced warming^13,15–20^. Recent estimates suggest the world is very likely to warm 2.5 °C to 3 °C by the end of the century, with Africa’s warming rate exceeding the global average^21–24^. Even under 2 °C of warming, the area affected by floods in many already flood-prone regions across Africa is projected to increase by up to 25%^25^, potentially leading to further loss of life, economic damage, and regional instability. Consequently, as Africa warms more rapidly than the global average, extreme precipitation events are projected to become more frequent and intense, underscoring the critical importance of understanding their physical drivers to improve adaptation strategies and guide effective mitigation efforts.

Rising global temperatures intensify the hydrological cycle primarily by increasing atmospheric moisture availability and modulating large-scale circulation patterns that govern regional precipitation extremes. A fundamental thermodynamic constraint is provided by the Clausius–Clapeyron (CC) relationship, which implies an approximately 7% increase in atmospheric water-holding capacity per degree Celsius of warming^26,27^. In response to global warming, global-mean precipitation is energetically constrained and is projected to increase more slowly, at roughly 2–3% per degree Celsius^26,28^, whereas short-duration precipitation extremes are more directly controlled by atmospheric moisture availability and vertical motion, and therefore often intensify at rates approaching or exceeding CC scaling. Scaling rates exceeding CC can arise from several mechanisms, including enhanced vertical motion during extreme events, increased precipitation efficiency, or shifts toward more convective precipitation regimes^29–31^. These processes highlight the important role of both thermodynamic moistening and dynamic amplification in shaping changes in precipitation extremes^3,5,32,33^. Across Africa, the impact of this thermodynamic intensification is strongly modulated by regionally relevant circulation features^34,35^. For instance, in northern Africa, including the Saharan and Mediterranean coast, extreme precipitation events are often associated with extratropical dynamics, including upper-tropospheric Rossby wave breaking and surface extratropical cyclones that drive anomalous moisture transport into an otherwise hyper-arid region^36–39^. In West Africa, extreme precipitation is tightly coupled to the dynamics of the West African Monsoon and the seasonal migration of the Intertropical Convergence Zone, which regulate deep convection and low-level moisture convergence over the Sahel and Guinea Coast^40–43^. In equatorial and central Africa, enhanced low-level moisture convergence and Walker-type circulation anomalies related to Atlantic and Indian Ocean thermal forcing play a dominant role in shaping extreme precipitation^44,45^, consistent with the region’s sensitivity to thermodynamic moistening and vertical-motion anomalies^46,47^. In East Africa, interannual and decadal variability in extreme precipitation is strongly influenced by sea-surface temperature forcing in the tropical oceans, particularly the Indian Ocean Dipole (IOD) and the El Niño–Southern Oscillation (ENSO)^48–51^. Southern Africa, while generally drought-prone, experiences episodic extreme precipitation associated with tropical cyclones and synoptic-scale circulation features, such as cut-off lows, which can produce devastating short-duration precipitation events, as exemplified by Cyclone Idai in 2019^52–54^. Under continued global warming, the combined effects of thermodynamic moistening and these regionally specific circulation responses are expected to increase the frequency and intensity of extreme precipitation across Africa, even in regions where mean precipitation is projected to decline^4,55,56^. Despite this well-established physical understanding, large intermodel spread continues to limit confidence in quantitative projections of future precipitation extremes across Africa, motivating the need for physically grounded approaches to constrain uncertainty.

Emergent-constraint (EC) approaches have emerged as one of the most powerful frameworks for reducing the large intermodel spread in projections of precipitation characteristics as well as underlying physical mechanisms, thereby providing more policy-relevant information on future extremes^57,58^. For instance, numerous studies have constrained end-of-21st-century changes in global precipitation characteristics (e.g., mean, extremes) by exploiting statistical relationships between historical trends of mean/extreme precipitation or global mean surface temperature, reporting reductions in projection uncertainties of ~20–40%^59,60^. In this EC framework, physically interpretable relationships are identified across an ensemble of Earth system models between an observable feature of the present climate (the predictor) and a future response (predictand), so that combining these relationships with observations systematically narrows the range of plausible projections for quantities, such as climate sensitivity, hydrological intensification, or regional precipitation extremes^57,61–64^. Importantly, for such a constraint to be credible, the emergent relationship must satisfy statistical rigor and be grounded in a well-understood physical basis. To address these requirements, recent studies have formalized EC theory within hierarchical emergent-constraint (HEC) frameworks, which explicitly account for uncertainties in present–future model relationships, structural model biases, and observational error, yielding a statistically rigorous and physically grounded constraint. Applications of HEC have demonstrated substantial reductions in uncertainty in projections of mean and extreme precipitation mostly at global scales^64–66^. Exploring the applicability of HEC to constrain subregional projections of extreme precipitation across Africa, while isolating the relative roles of thermodynamic and dynamic processes, therefore represents a credible pathway for narrowing uncertainty in future hydroclimate projections.

Although previous studies have documented increases in short-duration precipitation extremes across Africa^4,33,46,67^, substantial uncertainties remain regarding the magnitude, drivers, and societal implications of these changes at subregional scales. While the intensification of precipitation extremes under global warming is broadly consistent with increased atmospheric moisture availability following the CC relation, the extent to which thermodynamic scaling interacts with subregional circulation processes across African climate regimes remains poorly constrained in CMIP6 projections. Most existing studies have focused primarily on quantifying projected increases in precipitation intensity but have provided limited insight into the physical mechanisms governing subregional variations or into reducing the large intermodel spread that limits confidence in regional risk assessments.

Here, we address these challenges using an integrated framework that combines hierarchical emergent constraints, process-based physical diagnostics, and socioeconomic exposure analysis. We apply an HEC constraint framework linking observed historical global temperature trends to projected changes in annual maximum one-day precipitation (Rx1day) across Africa and its subregions, providing an observationally informed constraint on regional extreme precipitation projections. We then diagnose the physical mechanisms underlying projected changes using a precipitation-scaling framework that explicitly separates thermodynamic and dynamic contributions, complemented by diagnostics of surface and thermodynamic energy budgets to identify the processes controlling the changes in atmospheric moisture and vertical motion across African subregions. Finally, we quantify projected changes in rare high-impact events, including historically occurring 1-in-50-year and 1-in-100-year extremes, and translate both unconstrained and constrained projections into quantitative estimates of future population and GDP exposure, providing a direct link between climate dynamics and societal risk.

Together, this integrated approach provides a physically grounded, interpretable, and policy-relevant assessment of future extreme precipitation and exposure across African subregions.

## Results

### Unconstrained projected precipitation extremes

**Fig. 1:**
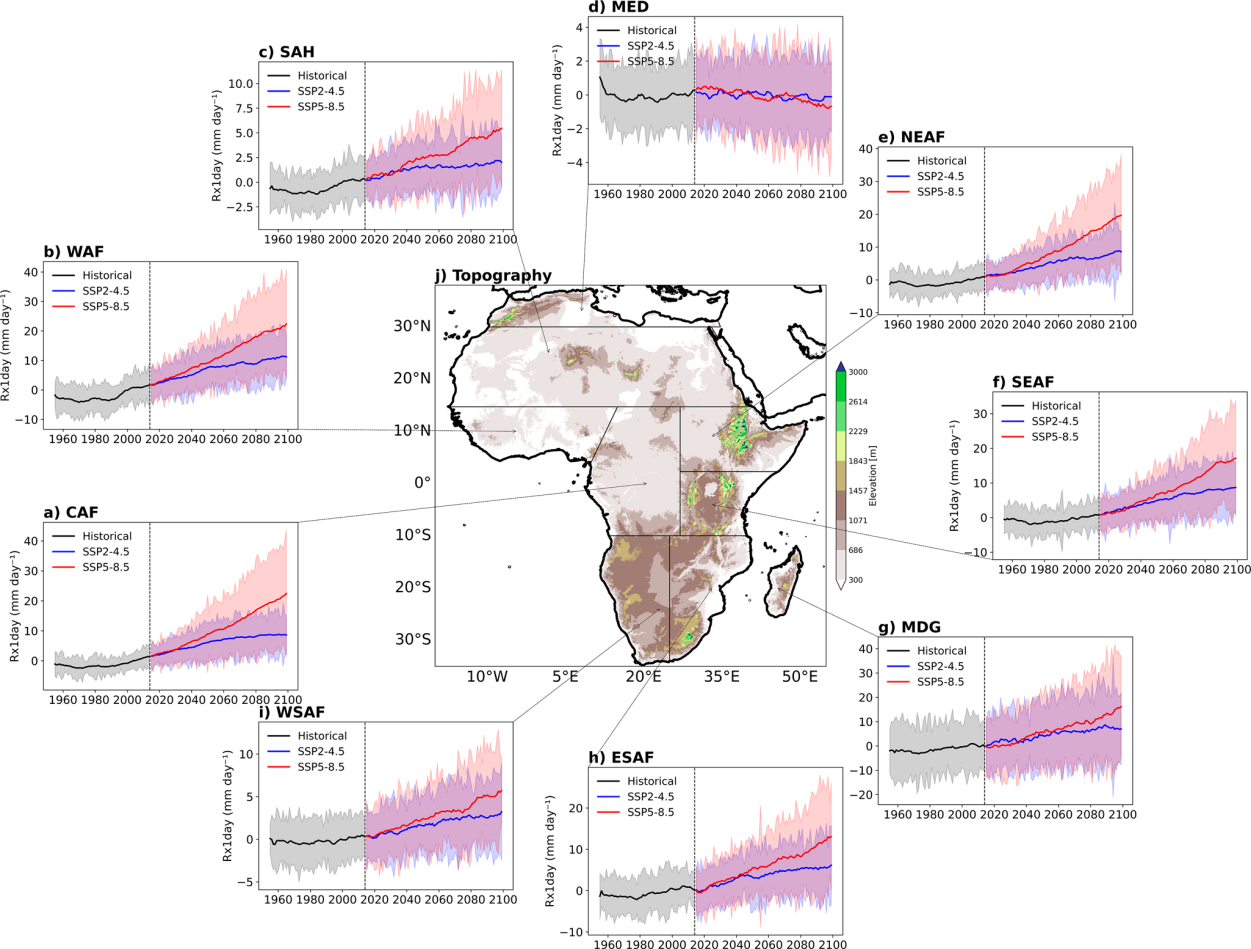
Projected regional changes in annual precipitation extremes (Rx1day) under the SSP2-4.5 and SSP5-8.5 scenarios. **a–i** Weighted area-averaged time series of Rx1day (mm day−1) over nine African regions. The black, blue, and red solid lines represent the CMIP6 ensemble mean (EnsMean) for the historical, SSP2-4.5, and SSP5-8.5 scenarios, respectively. The time series are computed as annual anomalies relative to the historical mean (1985–2014), followed by application of a 10-year running mean. Shaded areas indicate EnsMean ± 1 standard deviation, illustrating intermodel spread for both historical and future projections. **j** Topography (m) derived from the GTOPO30 digital elevation model.

We first assess the overall response of precipitation extremes to global warming by examining the temporal evolution of annual maximum 1-day precipitation (Rx1day) over African subregions using the raw/unconstrained CMIP6 models under the SSP2-4.5 and SSP5-8.5 scenarios (Fig. 1). Across Africa, Rx1day increases robustly throughout the 21st century, with clear scenario-dependent divergence emerging after the middle of the century. In the near term (2020–2040), projected changes are modest and exhibit relatively limited spread across ensemble members. The model ensemble mean (i.e., EnsMean) shows that both scenarios produce similar increases of approximately 1–3 mm day⁻¹ relative to the 1985–2014 historical baseline (Fig. 1). By the middle of the century (2041–2060), the two scenarios begin to separate more distinctly, especially in equatorial regions dominated by deep tropical convection, including West Africa (WAF; Fig. 1b), Central Africa (CAF; Fig. 1a), Northeast Africa (NEAF; Fig. 1e), and Southeast Africa (SEAF; Fig. 1f). In these regions, Rx1day increases to about 5–7 mm day⁻¹ under SSP2-4.5, whereas SSP5-8.5 produces substantially larger increases of roughly 7–12 mm day⁻¹. Ensemble spread also increases during this period, indicating larger intermodel uncertainty. The late-century period (2070–2099) shows the most spatially coherent and robust intensification of Rx1day. Under SSP2-4.5, increases generally range from 5 to 10 mm day⁻¹, with the smallest changes in subtropical southern Africa and the largest changes in the equatorial regions. Under SSP5-8.5, intensification accelerates rapidly after ~2050, leading to late-century increases that typically reach 15–23 mm day⁻¹ in equatorial regions. In several regions, especially CAF and Madagascar (MDG; Fig. 1g), the upper part of the ensemble exceeds 30 mm day⁻¹, suggesting the possibility of unprecedented single-day precipitation extremes. Although the magnitude of change and the associated uncertainty are greater under SSP5-8.5, the overall direction of change is consistent in both scenarios. Given that the most robust and clearly separable changes occur in the late century, the remainder of this study focuses on anticipated changes during the 2070–2099 period.

**Fig. 2:**
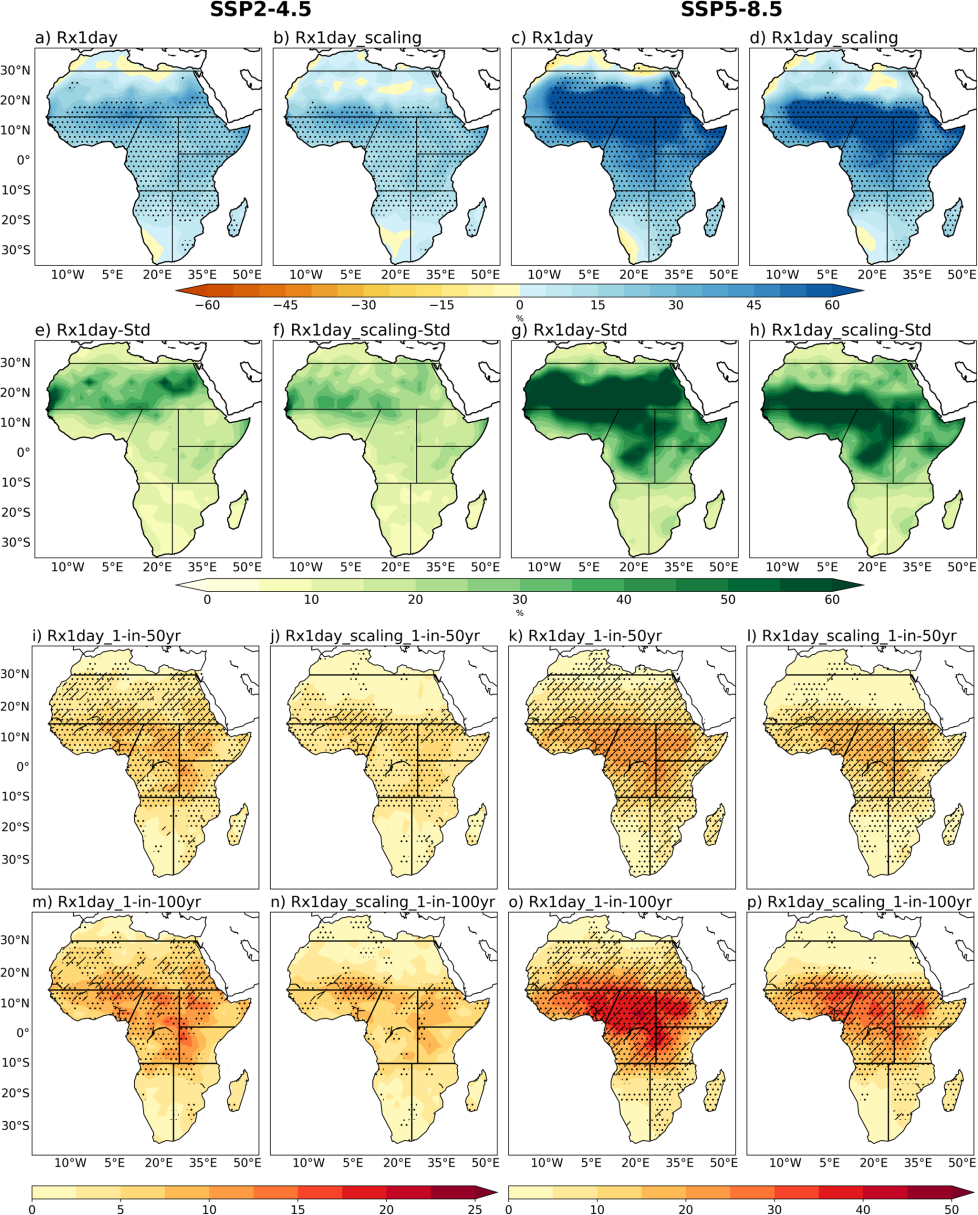
Projected changes in precipitation extremes and their scaling across Africa. **a–d** EnsMean changes in annual 1-day maximum precipitation (Rx1day) and precipitation-extreme scaling (%) under the SSP2-4.5 and SSP5-8.5 scenarios. Stippling denotes regions where at least 70% of models agree on the sign of the EnsMean change. **e–h** Multimodel standard deviation in Rx1day and Rx1day_scaling (%) under the SSP2-4.5 and SSP5-8.5 scenarios. **i–p** Probability ratios (PR) of (**i**,**k**,**m**,**o**) Rx1day and (**j**,**l**,**n**,**p**) Rx1day_scaling frequency relative to the historical baseline period (1985–2014), estimated using the Gumbel extreme value distribution for (i-l) 1-in-50-year and (**m–p**) 1-in-100-year return periods under SSP2-4.5 (**i**,**j**,**m**,**n**) and SSP5-8.5 (**k**,**l**,**o**,**p**). Stippling and hatching in (**i–p**) denote grid points where at least 70% of models agree on the increase/decrease in EnsMean and where the PR is statistically significant based on bootstrapping, respectively.

We further analyze the late 21st-century (2070–2099) spatial and regional-scale changes in Rx1day under SSP2-4.5 and SSP5-8.5 (Fig. 2 and Supplementary Fig. S1). We compare EnsMean of unconstrained projections of Rx1day derived directly from model precipitation with those estimated using a physical-scaling diagnostic^31,68,69^ (hereafter “Rx1day_scaling”; see Methods), which enables further investigation of the physical mechanisms underlying projected changes in precipitation extremes. Our results show that EnsMean of Rx1day_scaling closely reproduces the model-simulated pattern during the historical 1985–2014 period (Supplementary Fig. S2), with a spatial correlation > 0.88 and a root mean square difference of ~6.6 mm day⁻¹ (Supplementary Fig. S3a, b). Although Rx1day_scaling yields slightly lower intensities than the simulated Rx1day (Supplementary Fig. S2f), particularly across equatorial Africa, this discrepancy likely reflects simplifying assumptions inherent in the scaling framework. In particular, the diagnostic does not explicitly account for variations in precipitation efficiency^31^ or changes in updraft strength (vertical velocity) during extreme events, both of which can enhance condensation rates and precipitation intensity^30,31^. Additional nonlinear processes associated with convective organization and mesoscale dynamics may also contribute to departures between the scaling estimate and fully simulated extremes, highlighting an important caveat when interpreting precipitation-scaling diagnostics. Nevertheless, both the simulated Rx1day and Rx1day_scaling capture the dominant continental-scale pattern evident in all four observational products (CHIRPS, GPCC, ARC2, and CPC), characterized by relatively drier conditions in the subtropics and wetter conditions in the equatorial rain belt (Supplementary Fig. S2a–f). We note that these observations exhibit varying Rx1day magnitudes; most notably, compared to the other three observational datasets, GPCC exhibits higher Rx1day over CAF, NEAF, and SEAF (Supplementary Fig. S2b). We address this observational uncertainty by using their ensemble mean (EnsMean_Obs). Relative to this reference, both the simulated Rx1day and Rx1day_scaling overestimate EnsMean_Obs Rx1day over WAF and southern Africa (Supplementary Fig. S2g, h). Nonetheless, despite these regional discrepancies, the close agreement in spatial patterns demonstrates that CMIP6 Rx1day (both simulated and Rx1day_scaling) provides a credible and physically interpretable representation of historical Rx1day, supporting its use in subsequent analysis.

Similarly, as shown in Fig. 2a–d and Supplementary Fig. S3c, f, Rx1day_scaling also reproduces the projected end-of-century changes in Rx1day with high fidelity, exhibiting pattern correlations exceeding 0.9 across Africa except over the Sahara (SAH), where correlation values are around 0.7 and Rx1day_scaling yields weaker projected changes in both scenarios (Supplementary Fig. S4). Nevertheless, our results are consistent with previous global assessments based on CMIP5/6^31,70^, which reported that Rx1day intensifies across most land regions but decreases over parts of the subtropics. To further quantify the magnitude of projected intensification, we examine the relative change in Rx1day, defined as the ratio of future to historical Rx1day values. This metric highlights pronounced regional contrasts in projected Rx1day across Africa (Supplementary Fig. S5). NEAF shows the strongest intensification, with ratios of 1.25 and 1.56 under SSP2-4.5 and SSP5-8.5, respectively, compared to Africa-wide increases of 1.15 and 1.32, respectively. The northern and central Africa subregions (SAH, WAF, CAF, NEAF) also exceed a ratio of 1.4 under SSP5-8.5, whereas MED is the only exception, exhibiting a slight projected decrease (~1–3%). Overall, we find a continent-wide, robust intensification of extreme precipitation by the end of the 21st century, with at least 70% of the models agreeing on the sign of EnsMean across most regions (Fig. 2a–d). Similar projected increases are also evident for the 95th and 99th precipitation percentiles (Supplementary Fig. S6). Both the magnitude and spatial coherence of these changes increase substantially from SSP2-4.5 to SSP5-8.5. Under SSP5-8.5, area-weighted regional EnsMean increases in Rx1day exceed 40–50% across SAH and extend into WAF and CAF, while other equatorial regions show widespread intensification of 35–55% (Supplementary Fig. S1). Although smaller in magnitude, increases under SSP2-4.5 (15–25%) remain similarly robust. We note that despite the widespread projected intensification, substantial uncertainties persist regarding the magnitude of future Rx1day changes under both scenarios (Fig. 2e–h), underscoring the need for approaches to narrow the range of projected changes. Consistent with previous studies that have identified large uncertainties in model projections of tropical precipitation extremes^31,71,72^, we find that uncertainties in projected changes in Rx1day and Rx1day_scaling (Fig. 2e–h) are largest over SAH, CAF, and northern parts of WAF, with values reaching about 60% under SSP5-8.5 (Fig. 2g–h) and ~45% under SSP2-4.5 (Fig. 2e–f). While projected changes in Rx1day provide insight into the overall intensification of precipitation extremes, assessing societal risk also requires examining how the frequency of rare extreme events evolves under warming.

### Projected changes in rare precipitation extremes

We further assess how the regional frequency of rare, high-impact precipitation extreme events, which have major implications for climate-risk and adaptation planning, is projected to change across African subregions (Fig. 2i–p and Supplementary Figs. S7 and S8). We quantify these changes using the probability ratio (PR), which measures how the expected frequency of historically rare events changes under future climate conditions. Historically rare events are defined here as 1-in-50-year and 1-in-100-year annual Rx1day, estimated relative to the 1985–2014 baseline (see Methods). The PR is calculated using both the Gumbel distribution and, for comparison, the generalized extreme value (GEV) distribution. We assess the robustness in PR by bootstrapping each model 1000 times to assess significance at the 95% confidence interval and grid points where at least 70% of the models agree that the PR is significant (see Methods). A PR of 1 indicates no change in event frequency, whereas values greater than 1 signify that events historically considered rare become increasingly common in a warmer climate^23^. For example, a PR of 50 for a 1-in-50-year event implies that, under the statistical assumptions of the fitted extreme-value distribution, such an event would be expected to occur, on average, once per year in the future climate, highlighting substantial risks for agriculture, water resources, and infrastructure. We note that this interpretation reflects a change in the underlying probability distribution and does not imply deterministic annual occurrence.

Based on the Gumbel framework (Fig. 2i–p, and Supplementary Fig. S7a, b), we find that events that historically occurred once every 50 years across Africa are projected to occur, on average, approximately 5 and 11 times within a 50-year period under SSP2-4.5 and SSP5-8.5, respectively, with local increases reaching up to about 20 times the historical frequency (Fig. 2i–l, and Supplementary Fig. S7a). Similarly, historical 1-in-100-year events are projected to occur about 7 and 18 times within a 100-year period under SSP2-4.5 and SSP5-8.5, respectively (Fig. 2m–p, and Supplementary Fig. S7b), with local increases reaching up to 45 times the historical frequency. This implies that precipitation extremes currently occurring once every 50 years are projected to recur approximately once every 10 years under SSP2-4.5 and once every 5 years under SSP5-8.5, while events historically occurring once every 100 years are projected to recur approximately once every 14 years under SSP2-4.5 and once every 5.5 years under SSP5-8.5. These increases are robust and statistically significant for both the 1-in-50-year and 1-in-100-year return levels in SAH, WASF, NEAF, and SEAF under SSP5−8.5, whereas under SSP2-4.5 the robustness of the signal varies across subregions (Fig. 2i–p). Moreover, the magnitude of the projected increase in event frequency exhibits pronounced regional heterogeneity. Subregions dominated by deep tropical convection, including WAF, CAF, NEAF, and SEAF, show the largest PR values, with historical 1-in-50-year and 1-in-100-year events projected to recur, on average, every 2.5 to 3 years by the end of the 21st century under SSP5-8.5. In contrast, more arid or subtropical regions, such as MED, SAH, ESAF, WSAF, and MDG exhibit comparatively smaller, though still positive, increases in event frequency. The scaling results show a pattern broadly similar to the raw projections, except in SAH (20°N and above), where the increase is not robust, and fewer than 70% of models agree that the change is significant.

In addition, results based on the GEV distribution shown in the Supplementary Material (Supplementary Figs. S7c, d, S8) exhibit quantitatively similar spatial increases but are less robust across subregions and return levels, with statistically robust patterns confined primarily to WAF, CAF, NEAF, and SEAF under SSP5-8.5 for the 1-in-50-year return period. These results should be interpreted probabilistically and with the uncertainty expected when estimating very rare-event probabilities from finite samples, most notably uncertainty in the fitted extreme-value parameters (see Methods) and the working assumption of a quasi-stationary climate during the future period. To better understand the physical drivers of these projected changes in precipitation extremes, we next examine the thermodynamic and dynamic processes controlling Rx1day intensification.

### Underlying physical mechanisms of projected regional precipitation extremes

**Fig. 3:**
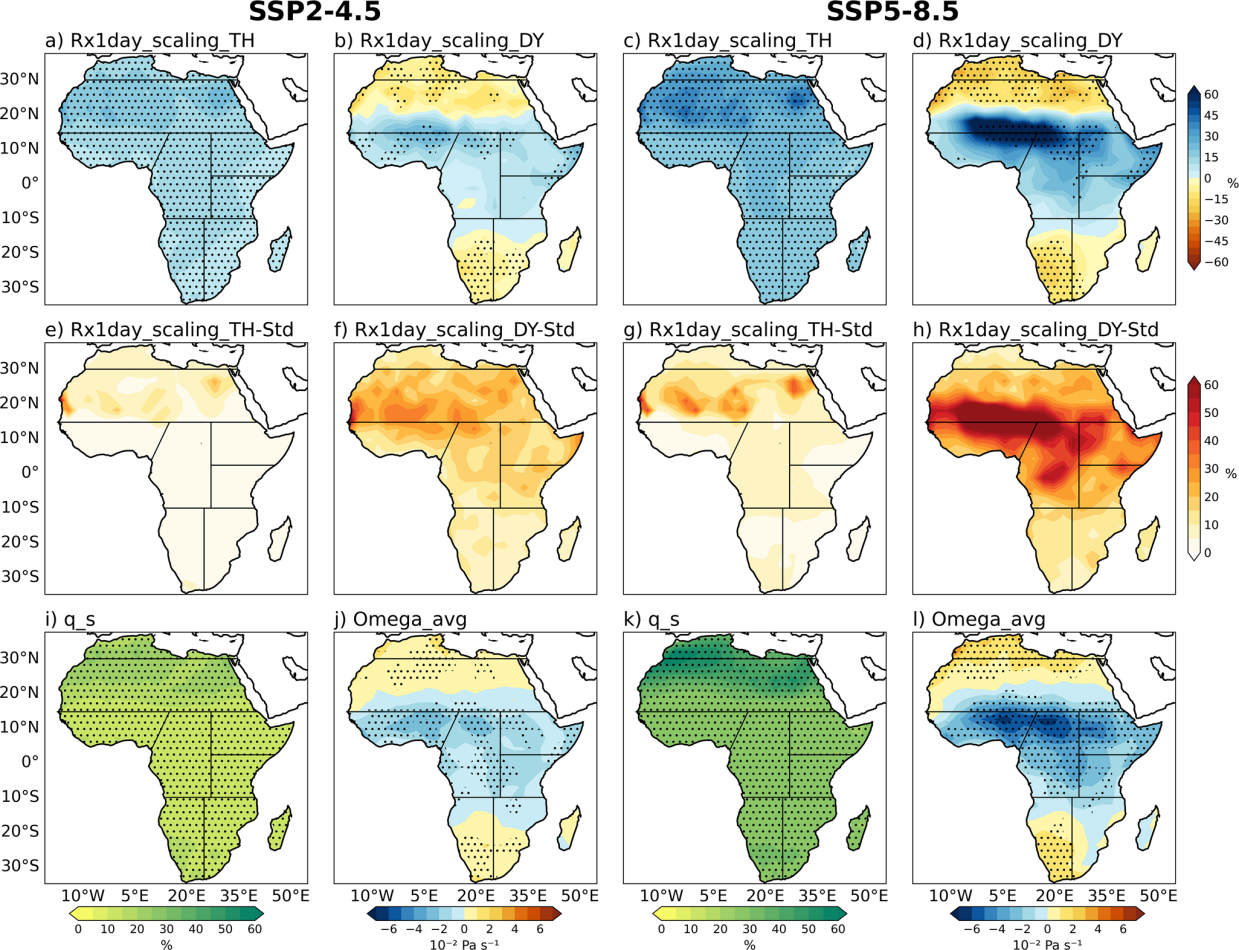
Projected changes in thermodynamic and dynamic scaling and the effects of saturation specific humidity and vertical motion. Ensemble-mean (EnsMean) changes (%) in (**a–d**) thermodynamic and dynamic scaling under the SSP2-4.5 and SSP5−8.5 scenarios. For thermodynamic scaling, vertical velocity (ω) is held fixed at its 1985–2014 mean, whereas for dynamic scaling, the vertical derivative of saturation specific humidity is held fixed at its 1985–2014 mean. **e–h** Multimodel standard deviation in thermodynamic and dynamic scaling (%) under the SSP2-4.5 and SSP5−8.5 scenarios. **i–l** EnsMean changes in vertically integrated saturation specific humidity (*qₛ*) and vertically averaged vertical velocity (negative values denote stronger ascent), both conditioned on extreme precipitation events under SSP2-4.5 and SSP5-8.5. Stippling denotes regions where at least 70% of models agree on the sign of EnsMean. Changes are calculated as the relative difference between the 2070–2099 and 1985–2014 means.

The physical-scaling diagnostic allows us to decompose precipitation extremes (i.e., Rx1day_scaling) into thermodynamic (TH) and dynamic (DY) components, enabling further investigation of the mechanisms driving projected changes (Fig. 3). The residual term, representing nonlinear interactions and transient eddy contributions, is also diagnosed but is not discussed in detail, as it accounts for only ~5% of the total change across most regions (Supplementary Fig. S9). Also, robust quantification of the mechanism modulating these nonlinear and transient processes would require higher temporal resolution data than are available from the CMIP6 models used herein. Nevertheless, our results show that the intensification of Rx1day_scaling over most parts of Africa in both scenarios is attributable largely to positive contributions from the TH component, which dominates the spatial pattern of change across regions (Fig. 3a, c and Supplementary Fig. S10e, g). In fact, the forced changes in TH scaling are consistently positive and broadly follow CC scaling, generally ranging between 5 and 10% K⁻¹. These changes are spatially relatively homogeneous, with moderate regional amplification that is strongest over SAH (Supplementary Fig. S10e, g). Regional mean contributions from the TH component exceed 20% under SSP5-8.5 and 10% under SSP2-4.5 (Supplementary Fig. S1), while the associated forced changes exceed 5% K⁻¹ in both scenarios (Supplementary Fig. S11). The strongest increases occur over SAH, reaching approximately 25% in SSP5-8.5 and 15% in SSP2-4.5, corresponding to about 10% K⁻¹ in both scenarios for this region. Consistent with this dominance, the pattern correlation between Rx1day_scaling and the TH component exceeds 0.6 in most regions, indicating that thermodynamic processes explain a substantial fraction of the spatial variability in projected precipitation extremes. However, this relationship also implies that a considerable portion of the variance remains unexplained by TH alone, highlighting the important contribution of dynamic processes and other regional factors in shaping the spatial distribution of Rx1day changes. Additional regression analysis further shows that the TH contribution is strongest across most African regions (Supplementary Fig. S12).

In contrast, the DY component exhibits pronounced regional contrasts, with robust decreases over WSAF and the northern SAH and MED (Fig. 3b, d). These reductions are associated with weaker upward motion, which acts to dampen the TH amplification of extremes (Fig. 3j, l). We note that the regions associated with projected decreases in DY components fall mostly in the northern and southern parts of the North Atlantic storm track, where most precipitation extremes are associated with extratropical cyclones^31,69^. This pattern is consistent with projected meridional contraction of the winter storm track and a reduction in cyclone frequency^73–75^. Although DY effects are stronger under SSP5−8.5 (Fig. 3d), they are generally insufficient to offset the TH signal, resulting in a net intensification of precipitation extremes across most regions (Fig. 2d). The only exceptions are WAF, CAF, and NEAF, where the DY component intensifies and becomes the dominant driver under SSP5-8.5 (Fig. 3d and Supplementary Fig. S1c–e). Consistent with previous studies, which are mostly globally focused^31,76–78^, changes in the TH contribution are driven primarily by increases in vertically averaged saturation specific humidity during precipitation extremes (*qₛ*; Fig. 3i, k), whereas changes in the DY contribution are controlled by variations in vertical velocity (ωₑ; Fig. 3j, l).

Overall, the results presented herein are robust across scenarios, although the magnitude of change is substantially larger under SSP5-8.5. It is important to note that changes in the DY and TH components are physically coupled and not strictly independent, for example through alterations in moist static stability. Our analysis further indicates that uncertainty in Rx1day projections is dominated by changes in the DY component associated with variability in large-scale atmospheric circulation, whereas uncertainties in the TH contribution are generally small (Fig. 3e–h). This further highlights the robustness of the TH influence on future Rx1day changes. Importantly, despite considerable uncertainties in the magnitude of Rx1day change, particularly north of 10°S, the spatial pattern and sign of the Rx1day response to global warming remain relatively robust across models.

### Mechanisms controlling projected changes in TH and DY components

**Fig. 4:**
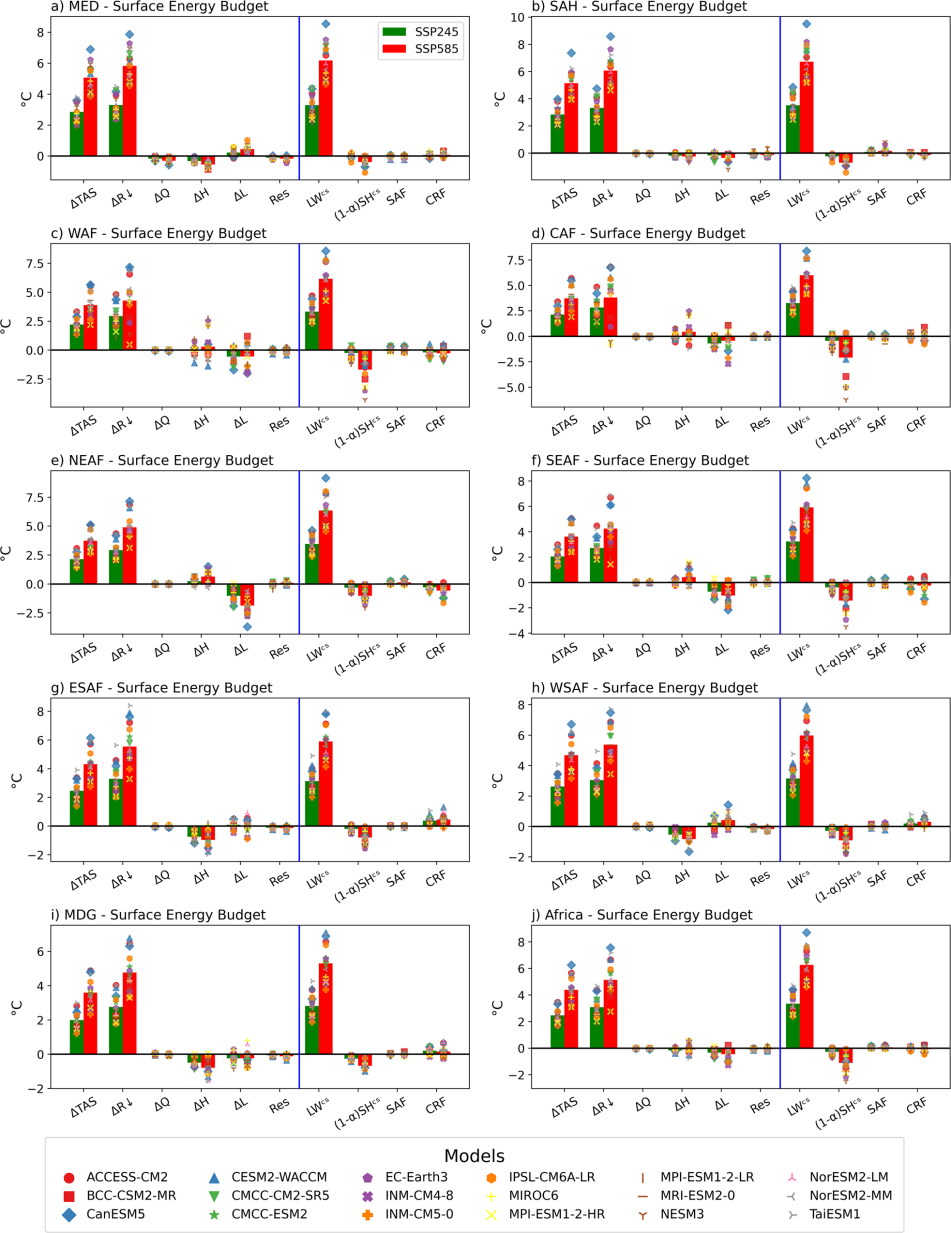
Projected changes in the surface energy budget. **a–j** Area-weighted, averaged diagnostics of the surface energy budget and its radiative components (ΔR ↓ ) over all of Africa and its subregions under the SSP2-4.5 and SSP5−8.5 scenarios. Bars indicate EnsMean, while markers denote individual model estimates. Changes are calculated as the difference between the 2070–2099 and 1985–2014 means.

Having established the relative contributions of TH and DY processes to changes in precipitation extremes, we now examine the physical mechanisms controlling these components across African subregions. Consistent with the thermodynamically driven projected increase in Rx1day over Africa, our results show a robust increase in future near-surface temperature, with SSP5−8.5 consistently exhibiting stronger warming in line with the larger projected increase in Rx1day. We find a strong and statistically significant correlation (r > 0.7, p < 0.05) between changes in *qₛ* (a key variable in calculating the thermodynamic contribution) and changes in near-surface temperature across most of Africa, with the exception of parts of the Sahara and Ethiopia (Supplementary Fig. S13a, c). This indicates that projected warming modulates increases in Rx1day primarily through intensification of thermodynamic processes (Supplementary Fig. S14). To investigate the physical mechanisms underlying near-surface temperature changes and, by extension, TH-induced Rx1day intensification, we diagnose the surface energy budget (SEB; see Methods) and examine key atmospheric variables related to land–atmosphere interactions and radiative processes. Across Africa and its subregions, near-surface temperature is projected to increase robustly by 2–6 °C, depending on the scenario and region (Supplementary Fig. S13b, d). The strongest warming is projected over MED and SAH, exceeding 5 °C, while in most other subregions warming is well below 5 °C under SSP5-8.5, with a similar but weaker pattern under SSP2-4.5. SEB decomposition reveals that net downwelling surface radiation (ΔR) is the dominant contributor to warming across all subregions, with generally negligible and non-robust contributions from heat storage and surface turbulent heat fluxes, except in MED, NEAF, SEAF, ESAF, WSAF, and MDG (Fig. 4). Over NEAF and SEAF, decreased latent heat flux partially offsets the radiative warming associated with enhanced ΔR, with a small positive contribution from sensible heat flux. In MED, ESAF, WSAF, and MDG, changes in sensible heat flux similarly act to slightly offset warming, though their overall influence on the SEB remains minor. Further decomposition of ΔR shows that increased net downwelling clear-sky longwave radiation (LWcs) is the primary driver of the projected net downwelling surface radiation increase (Supplementary Fig. S15c–d). This enhancement reflects greater trapping of outgoing longwave radiation due to increased greenhouse gas concentrations and atmospheric moisture, as supported by increases in total column precipitable water (Fig. 4 and Supplementary Fig. S15a, b). In addition, strengthened subtropical anticyclonic circulation over Africa is reflected in steeper 500 hPa geopotential height gradients relative to the historical period (Supplementary Fig. S15e–f). These enhanced gradients promote subsidence and more persistent warm conditions, with a stronger response poleward of 15°N and 15°S. While subsidence dynamically suppresses ascent, the resulting warming enhances the atmospheric water-holding capacity and total column moisture (Supplementary Fig. S15a–b), thereby providing a larger moisture reservoir that favors more intense precipitation extremes when such events occur.

**Fig. 5:**
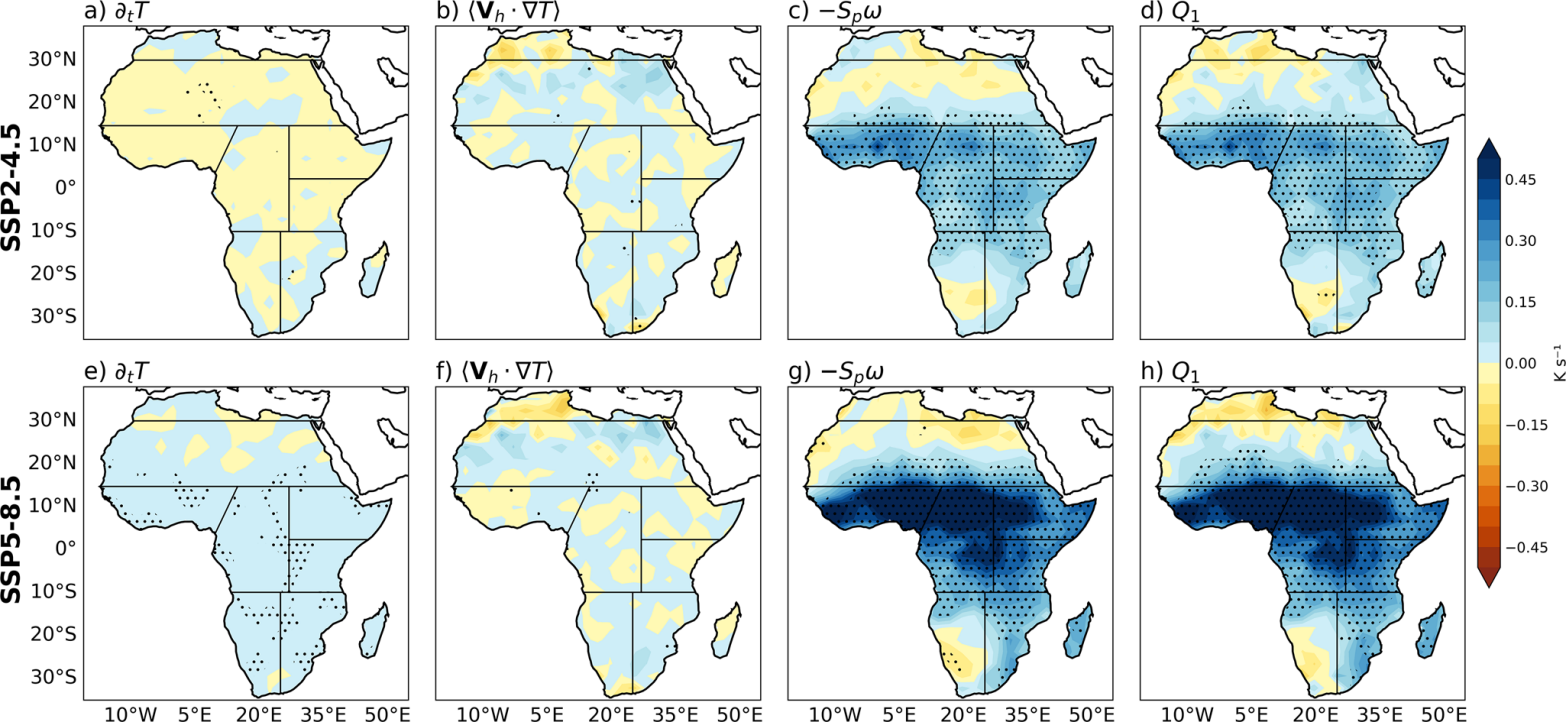
Projected changes in thermodynamic energy balance components. **a–h** EnsMean spatial changes in the thermodynamic energy balance terms under the SSP2-4.5 and SSP5-8.5 scenarios. Terms are computed following Eq. 12: ∂ₜT denotes the temperature tendency, 〈Vₕ · ∇T〉 the horizontal temperature advection, Sₚω the vertical temperature advection (omega) term (multiplied by −1 for direct comparison with Q₁), and Q₁ the total diabatic heating. Stippling denotes regions where at least 70% of models agree on the sign of EnsMean. Changes are calculated as the difference between the 2070–2099 and 1985–2014 means.

Furthermore, we diagnose the thermodynamic energy budget (see Methods) to investigate the physical mechanisms underlying the projected decrease or increase in the DY contribution. Decreased (increased) DY contributions are evident over WSAF as well as northern SAH and MED (CAF, WAF, NEAF, and SEAF; Fig. 3b, d) and are associated with weakened (enhanced) vertical velocity (Fig. 3j, l). Our results show that enhanced ascent in equatorial regions is driven primarily by increases in diabatic heating (Q₁), reflecting stronger latent heat release associated with intensified deep convection under future warmer and moister conditions (Fig. 5d, h). The vertical temperature advection term (−Spω) increases substantially in these regions (Fig. 5c, g) and exhibits a strong and statistically significant correlation with Q₁ (r > 0.9; p < 0.05; Supplementary Fig. S16), demonstrating a tightly coupled feedback between diabatic heating and large-scale vertical motion. In contrast, contributions from local temperature tendencies (∂T/∂t) and horizontal temperature advection (Vₕ·∇T) are weak and statistically insignificant, indicating a limited role for transient or advective processes. Overall, these results suggest that extreme precipitation intensification over most of equatorial Africa not only arises from thermodynamic moistening but is further amplified by dynamically enhanced ascent, driven by increased diabatic heating. In contrast, over subtropical regions, such as WSAF and SAH, contributions from horizontal temperature advection are somewhat comparable in magnitude to diabatic heating (Fig. 5b, d, f, h). These signals are consistent with projected shifts and modifications of midlatitude storm tracks that alter baroclinic eddy activity and moisture transport into subtropical regions^79,80^, contributing to reduced upward motion and limiting convective processes (and thus leading to reduced precipitation extremes) despite strong surface warming.

### Sources of projection uncertainty

**Fig. 6:**
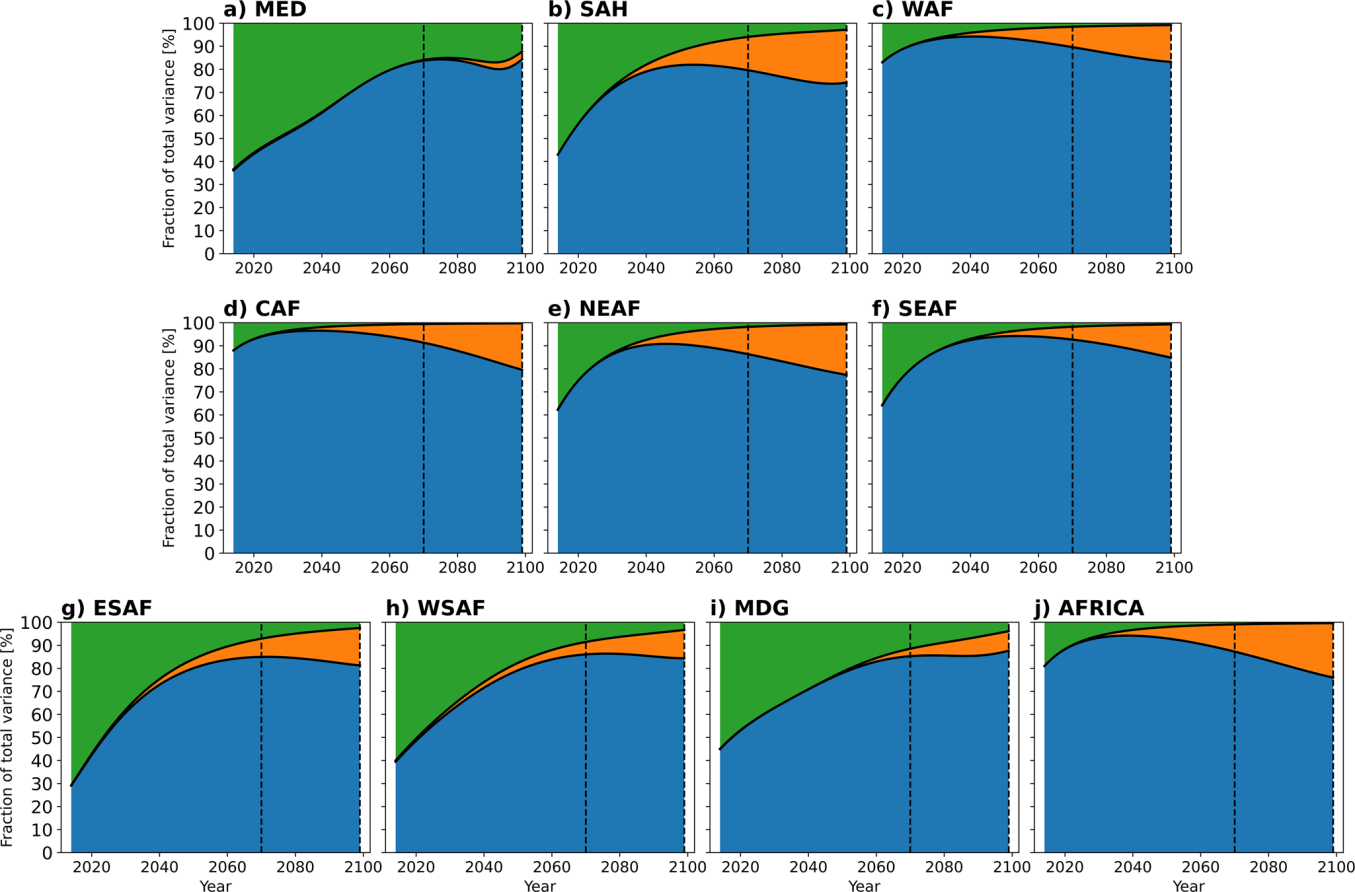
Temporal evolution of uncertainty components. Percentage of total variance in projected annual maximum 1-day precipitation (Rx1day) attributable to the three sources of uncertainty is shown for the African subregions (**a–i**) and for all of Africa (**j**). Blue, green, and orange shading denote model, internal variability, and scenario uncertainty, respectively.

The mechanistic understanding developed above also provides a useful framework for interpreting the sources of uncertainty in projected precipitation extremes. As earlier reported, the projections of Rx1day over Africa and its subregions exhibit substantial uncertainty, with consistently larger intermodel spread under SSP5-8.5 compared to SSP2-4.5. Here, we identify the sources of projection uncertainty by decomposing the total variance in decadal Rx1day projections across the 21st century into contributions from model uncertainty, scenario uncertainty, and internal variability (Fig. 6). Our results show that, across all regions, uncertainty from internal variability (green shading) decreases with lead time, whereas scenario uncertainty (orange shading) generally emerges around mid-century (2030–2040) and increases toward the end of the century. Model uncertainty (blue shading) contributes the most to total variance throughout the projection period, although its fractional contribution slightly decreases in the late 21st century in some regions as scenario uncertainty grows. Overall, model uncertainty dominates the projection uncertainty, accounting for more than 70% of the total variance in Rx1day projections across most African regions and reaching up to about 85% over MDG. Scenario uncertainty, although less influential, becomes the second-largest contributor by the late century, with its highest contribution (about 25% of the total variance) occurring over SAH. Although the magnitudes differ slightly, the variance decomposition of Rx1day_scaling reveals a broadly similar pattern (Supplementary Fig. S17). We further apply the variance decomposition to the TH and DY components of Rx1day_scaling to investigate drivers of uncertainty (Supplementary Figs. S18 and S19). While internal variability likely reflects fluctuations in large-scale atmospheric circulation and vertical motion that modulate the DY component of extreme precipitation, our results reveal that across all subregions, scenario uncertainty is driven primarily by TH processes (Supplementary Fig. S18), whereas model uncertainty is associated largely with DY processes (Supplementary Fig. S19). These have been linked to differences and/or misrepresentations of subgrid-scale processes, especially deep convection, cloud microphysics, and boundary-layer turbulence^34^, as well as differences in how models represent regional atmospheric circulation patterns that regulate moisture transport and vertical motion^81,82^. We note that the potential for directly constraining these DY processes using historical observations is limited by the availability of observed data for dynamic variables over Africa. Furthermore, large-scale circulation changes over Africa are often weak in the historical record and strongly influenced by internal variability. This limits the possibility of directly reducing model uncertainty associated with dynamic process representation through observational constraints alone. Consequently, the emergent-constraint framework applied in this study targets primarily the TH component. In addition, given that TH processes dominate the forced signal of precipitation extremes, this understanding motivates the use of an observationally informed emergent constraint based on historical global warming trends to reduce projection uncertainty.

### Constrained projected precipitation extremes over Africa

To improve the reliability of subregional Rx1day projections, we explore how the climate sensitivity of CMIP6 models can be leveraged to constrain future Rx1day changes. The historical global mean surface temperature (GMST) trend is a useful predictor for constraining future Rx1day projections because it partly reflects intermodel differences in the magnitude of the forced warming response over the historical period^64,77,83,84^. As a robust measure of large-scale thermodynamic forcing, GMST governs atmospheric moistening through CC scaling, whereby near-saturation specific humidity, and thus extreme precipitation intensity, can increase by about 7% per degree of warming. Consequently, models that simulate stronger historical GMST trends tend to exhibit larger increases in atmospheric water vapor (Supplementary Fig. S20) and, in turn, stronger projected intensification of precipitation extremes. GMST therefore serves as a more stable proxy for the large-scale thermodynamic forcing that governs atmospheric moisture availability and the intensity of precipitation extremes. In addition, GMST may also be indirectly linked to changes in atmospheric circulation, such as variations in cross-equatorial flow associated with shifts in interhemispheric thermal gradients^85^. To minimize the influence of anthropogenic aerosols, which have exhibited a nearly constant global emission rate since 1981^24,83^, we restrict the calculation of historical warming trends to the post-1980s period. The GMST trend over 1985–2014 is used as the predictor for our HEC (see Methods). It is important to note that the GMST trend is employed here strictly as a statistical predictor that captures intermodel differences in large-scale thermodynamic response, rather than as a mechanistic or causal driver of regional precipitation extremes. We compute this trend using ordinary least squares regression^59,64,86^. For gridded temperature observations, we use the HadCRUT4 (Hadley Center/Climatic Research Unit Temperature version 4)^87^ and GISTEMP4 (Goddard Institute for Space Studies Surface Temperature Analysis version 4)^88^ datasets.

**Fig. 7:**
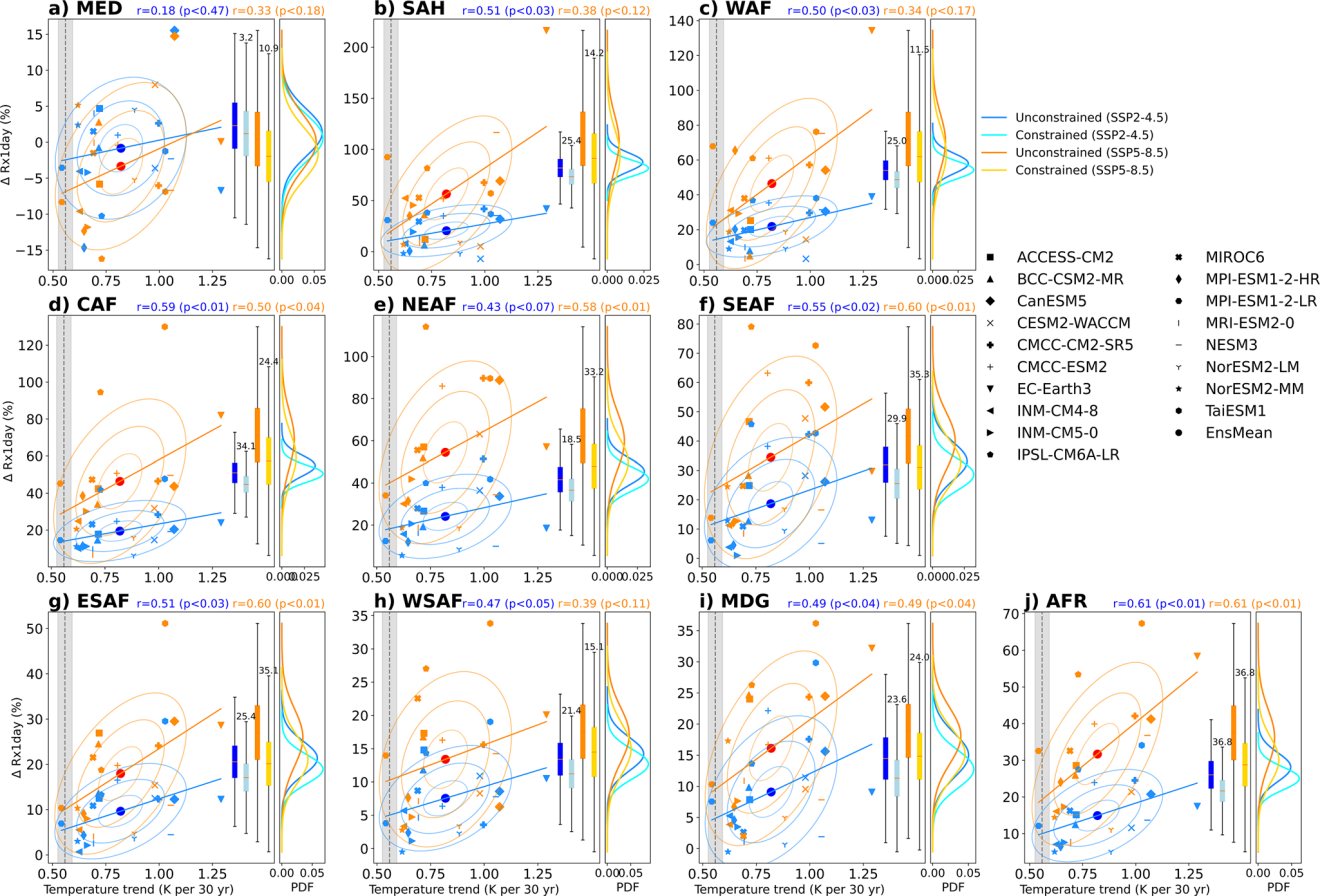
Observationally constrained projected changes in annual maximum 1-day precipitation (Rx1day). **a–i** African subregions and (**j**) all of Africa under the SSP2-4.5 and SSP5−8.5 scenarios. The y-axis shows projected changes in Rx1day from individual CMIP6 models, while the x-axis shows historical (1985–2014) global mean surface temperature (GMST) trends (K per 30 years). Markers represent individual CMIP6 models, and the blue and orange lines denote the emergent relationships (see Methods). The black vertical dashed line indicates the observed historical GMST trend, with uncertainty given by ± 1 standard deviation (gray shading). Ellipses represent the bivariate probability density functions of historical GMST trends and future Rx1day changes for SSP2-4.5 (light blue) and SSP5-8.5 (orange). Box plots summarize the unconstrained and constrained distributions, showing the median, interquartile range (25th–75th percentiles), and whiskers extending to 1.5 times the interquartile range, assuming a Gaussian distribution based on EnsMean and variance. The numbers on the boxes indicate the relative reduction of variance (RRV) for both scenarios. Curves on the right side of each panel show the corresponding Gaussian probability density functions of future Rx1day changes for the unconstrained and constrained projections under the SSP2-4.5 and SSP5-8.5 scenarios. Changes are calculated as the relative difference between the 2070–2099 and 1985–2014 means.

We test the validity of the emergent relationship at the subregional scale by examining intermodel relationships between projected changes in Rx1day and historical GMST trends (Fig. 7). As reported in previous global studies^83^, CMIP6 models tend to simulate stronger historical warming trends than observed, partly due to positive cloud feedback^89,90^. Our results show a statistically significant (p < 0.05) positive relationship between changes in Rx1day and historical GMST trends across most subregions under both SSP2-4.5 and SSP5-8.5. Correlations reach up to 0.59 over CAF under SSP2-4.5 (Fig. 7c) and 0.60 over ESAF under SSP5-8.5 (Fig. 7f). This suggests that models with stronger historical global warming trends tend to project larger Rx1day intensification by the end of the 21st century under both scenarios. However, the strength of this relationship varies across regions. These findings imply that Rx1day intensification is expected to be weaker in constrained than in unconstrained projections, especially in subregions where the HEC holds. To better account for Rx1day intensification in relation to local feedback, one might consider constraining Rx1day using either local temperature trends or historical Rx1day trends. However, our results show that the correlation between changes in Rx1day and local historical temperature trends or local historical Rx1day trends is relatively weaker and often statistically insignificant compared to the relationship with historical GMST trends (Supplementary Figs. S21–22).

We use the historical GMST trends to constrain changes in Rx1day across Africa and its subregions, with emphasis on subregions where the HEC relationship holds. Consistent with previous global studies^59,77,84^, constrained Rx1day projections yield a weaker mean response than unconstrained projections, with reductions of approximately 11–35% across regions (Fig. 7). Notably, this brings the area-averaged projected relative increase in Rx1day from above 40% in unconstrained projections (as high as 56% in NEAF) to below 35% across all subregions, thereby reducing the most extreme projected signals (Supplementary Fig. S5). This reduction arises primarily because most CMIP6 models systematically overestimate observed historical warming rates, accompanied by substantial intermodel spread in the magnitude of these trends. Nevertheless, even after applying observational constraints, the resulting Rx1day projections still show robust intensification, underscoring the need for effective adaptation measures. This result is consistent under both SSP2-4.5 and SSP5-8.5 but is more pronounced under the high-emission scenario, highlighting the increasing predictive power of the emergent relationship as the climate system moves further away from natural variability. Furthermore, we find that the HEC substantially reduces projection uncertainty, primarily through a reduction in dynamic uncertainty (Supplementary Fig. S23), as constrained distributions are significantly narrower than those from the unconstrained EnsMean. Importantly, the added value of the HEC is strongly region dependent. The reduction in uncertainty is particularly evident in regions dominated by deep tropical convection. For example, in CAF, the relative reduction in variance (RRV; see Methods) reaches 34.1% under SSP2-4.5 (Fig. 7d), while in ESAF it reaches 35.3% under SSP5-8.5 (Fig. 7g). In these regions, thermodynamic processes associated with atmospheric moisture availability exert strong control over precipitation extremes. Accordingly, both unconstrained and constrained projections consistently indicate substantial increases in Rx1day relative to the historical period, consistent with the enhanced moisture-holding capacity of a warming atmosphere^2,12^. By contrast, in regions where circulation anomalies play a stronger reversing role, such as MED, WSAF, and SAH, the HEC relationships are weaker, particularly under SSP5-8.5, with RRVs of 10.9%, 15.1%, and 14.2%, respectively. Even in these regions, however, the emergent constraint still narrows the projected distributions and slightly reduces the spread of projected outcomes. Overall, across Africa, constrained projections indicate widespread and significant intensification of extreme precipitation. The continental-mean response increases by roughly one-third under SSP2-4.5 and slightly more under SSP5-8.5. Assuming a Gaussian probability density function (see Methods), constrained projections are not only narrower than the unconstrained EnsMean but also tend to show smaller Rx1day increases, suggesting that unconstrained projections may overestimate the magnitude of future changes.

In addition, we apply the HEC for projected Rx1day changes at global warming levels of 1.5 °C and 2 °C, providing further insight into its physical credibility and implications for future water-cycle changes (Supplementary Tables S2 and S3). At both warming levels, the HEC consistently reduces intermodel spread, with slightly stronger reductions at 2 °C. For example, at 2 °C warming, the unconstrained mean Rx1day change over SAH is 21.23 ± 14.10 mm yr⁻¹ under SSP5-8.5, while over SEAF, it is 9.96 ± 5.44 mm yr⁻¹ under SSP2-4.5. After applying the HEC, these estimates decrease to 15.48 ± 13.56 mm yr⁻¹ and 7.12 ± 5.10 mm yr⁻¹, respectively, corresponding to RRVs of 7.4% and 12.1%. Overall, the magnitude of variance reduction is generally modest, typically ranging from a few percent to about 10%, with the largest RRV (28.5%) found over MED at 2.0 °C under SSP5-8.5. These results further reinforce the benefits of emergent constraints for increasing confidence in subregional projections of hydrological extremes, which we now translate into potential societal exposure by combining them with demographic and economic projections.

### Impacts on urban and rural populations and gross domestic product

**Fig. 8:**
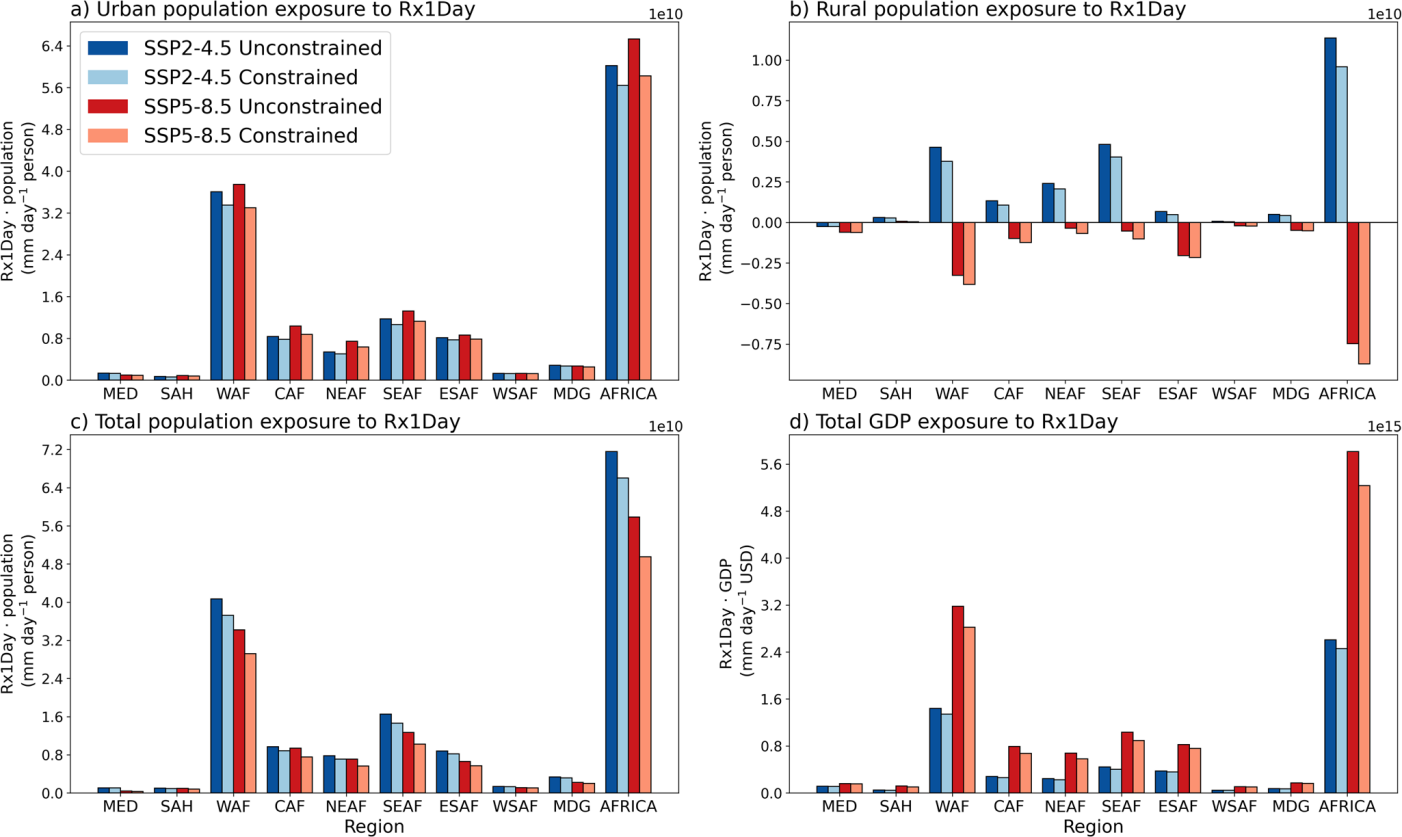
Population and economic exposure to extreme precipitation. Bar plots show the change in total exposure for unconstrained and constrained Rx1day for **a** urban population, **b** rural population, **c** total population, and **d** gross domestic product (GDP) across Africa and its subregions under the SSP2-4.5 and SSP5-8.5 scenarios. Change is calculated for the future period (2070–2099) compared to the historical period (1985–2014).

Extreme precipitation is a major hydrometeorological hazard in a warming climate, and Africa is particularly exposed due to rapid population growth, accelerating urbanization, and limited adaptive capacity. Increases in Rx1day can intensify flood risk, disrupt infrastructure, and undermine socioeconomic development, making robust assessments of future changes essential. We note that flood risk is also shaped by multiple interacting factors, including catchment characteristics, antecedent soil moisture conditions, flood defense measures, urban drainage capacity, and the vulnerability of built infrastructure, in addition to precipitation extremes and population exposure^13,91,92^. Here, we quantify the impacts of projected Rx1day changes on the exposure of urban and rural populations and gross domestic product (GDP) across Africa, comparing outcomes under unconstrained projections with those expected in constrained projections (Fig. 8). The area-weighted regional averages of population exposure reveal a consistent pattern, with urban and rural populations responding differently to the same climate forcing and together shaping total exposure (Fig. 8a–c). Our results show that model constraints consistently moderate the projected impacts. Decomposition of total exposure into climate, population/GDP, and interaction effects reveals that population/GDP changes are the dominant driver, followed by the interaction effect, with the climate effect being the smallest contributor, an order of magnitude lower than the others (Supplementary Figs. S24–26). The climate effect contributes positively across all regions, except for a minor negative contribution in the MED region, consistent with the decrease in Rx1day also noted in the relative change analysis (Supplementary Fig. S24). Urban populations experience substantial increases in exposure under both SSP2-4.5 and SSP5-8.5, regardless of whether projections are constrained or unconstrained. WAF exhibits the largest urban exposure, increasing from approximately 3.61 × 10¹⁰ person mm day⁻¹ under SSP2-4.5 to nearly 3.75 × 10¹⁰ under SSP5-8.5 in the unconstrained projections (Fig. 8a). The constrained EnsMean shows lower increases, around 3.35 × 10¹⁰ and 3.30 × 10¹⁰ person mm day⁻¹, respectively. CAF, NEAF, SEAF, and ESAF also show moderate increases, from about 0.5 × 10¹⁰ to nearly 1.1 × 10¹⁰ person mm day⁻¹. Increases in MED, SAH, WSAF, and MDG are relatively smaller but consistent with their population sizes. These upward trends indicate that both Rx1day intensification and demographic growth, reflected in the large population and interaction effects, contribute to increasing urban exposure across all subregions (Fig. 8a, and Supplementary Figs. S25a, S26a).

On the other hand, rural population exposure shows modest increases under SSP2-4.5 but declines under SSP5-8.5. WAF exhibits the largest negative rural exposure, approximately −0.33 × 10¹⁰ person mm day⁻¹ in the unconstrained SSP5-8.5 projections and −0.38 × 10¹⁰ under constraints (Fig. 8b). CAF, NEAF, SEAF, and ESAF also show modest negative rural exposure up to −0.2 × 10¹⁰ in the unconstrained and constrained SSP5-8.5 scenario. Decomposition analysis clarifies that this rural decline is driven primarily by the population effect, specifically the projected decrease in rural population due to high migration and urbanization under SSP5-8.5^93^, with the interaction effect compounding the reduction, while the climate effect remains positive (Supplementary Figs. S24–26b). These contrasting magnitudes and directions of change in urban and rural exposure influence total exposure. Because urban exposure is consistently positive and increases by several tens of billions of person mm day⁻¹, whereas rural exposure decreases by only a few billion, total regional averages closely follow urban trends. Even in regions where rural averages are strongly negative, such as WAF and ESAF, the magnitude of rural declines is much smaller than the increase in urban exposure. Consequently, total population exposure continues to rise under both emissions pathways. Under SSP2-4.5, Africa-wide total exposure reaches approximately 7.16 × 10¹⁰ person mm day⁻¹ in the unconstrained ensemble and ~6.60 × 10¹⁰ under constraints. Under SSP5-8.5, the unconstrained total falls to 5.78 × 10¹⁰, while the constrained mean is reduced to roughly 4.95 × 10¹⁰ (Fig. 8c). These lower increases in SSP5-8.5 compared to SSP2-4.5 are due primarily to weaker overall population growth in SSP5-8.5 in Africa^93^, rather than a negative change in rural exposure, which is also reflected in the population effect in urban exposure (Fig. 8a-c and Supplementary Figs. S24–S26a-c).

In contrast to population exposure, which is generally higher under SSP2-4.5, projected GDP exposure to Rx1day (Fig. 8d) is markedly greater under SSP5-8.5 across all African subregions, with model constraints similarly moderating but not reversing the direction of change. Under SSP2-4.5, Africa-wide GDP exposure reaches ~2.26 × 10¹⁵ USD mm day⁻¹ in the unconstrained EnsMean and is reduced to ~2.45 × 10¹⁵ USD mm day⁻¹ when constraints are applied. The amplification under SSP5-8.5 is much larger: unconstrained exposure climbs to ~5.81 × 10¹⁵ USD mm day⁻¹ and is reduced to ~5.24 × 10¹⁵ USD mm day⁻¹ with the constrained EnsMean. WAF dominates the continental signal, with unconstrained SSP5-8.5 exposure of ~3.18 × 10¹⁵ USD mm day⁻¹ compared to ~2.82 × 10¹⁵ under constraints, while CAF, NEAF, and SEAF (MED, SAH, WSAF, and MDG) exhibit moderate (or minimal) but consistent increases. Decomposition of GDP exposure confirms that all three effects contribute positively across subregions, except for the climate and interaction effects in MED (Supplementary Figs. S24–S26d). These result in a minor negative contribution that partially offsets the net positive increase in GDP exposure from the GDP effect in MED. Overall, these patterns reflect the underlying socioeconomic assumptions of the two scenarios. SSP2-4.5 represents moderate population growth and steady economic development, whereas SSP5-8.5 assumes similar but slightly weaker population growth, with high migration and urbanization leading to rapid GDP expansion^93,94^. Consequently, higher total populations under SSP2-4.5 contribute to higher total population exposure, while accelerated economic growth under SSP5-8.5 may increase GDP exposure in densely populated or rapidly urbanizing regions.

## Discussion

Projected changes in extreme precipitation across Africa show widespread and robust intensification of annual precipitation extremes (Rx1day) throughout the 21st century, with the strongest increases in equatorial Africa and under SSP5-8.5. Beyond confirming the general thermodynamic intensification reported in previous global assessments, this study provides a continent-wide, subregional assessments that simultaneously characterizes the magnitude, mechanisms, and uncertainty structure of African precipitation extremes within a unified analytical framework. Specifically, consistent with previous global assessments of CMIP5/6^31,64,70,77^, our results indicate that most African subregions will experience substantial intensification of precipitation extremes, even under moderate warming levels. The most pronounced increases occur in regions dominated by deep convection, such as WAF, CAF, NEAF, and SEAF, where Rx1day increases by about 5–7 mm day⁻¹ under SSP2-4.5 and 7–12 mm day⁻¹ under SSP5-8.5. Although uncertainty in magnitude emerges after 2023 and grows toward the end of the century, models strongly agree that by the late century (after 2070), extreme precipitation intensification becomes spatially coherent across nearly all African subregions, with scenario-dependent divergence particularly pronounced under SSP5-8.5. Our analysis extends beyond intensity changes by explicitly quantifying shifts in the return periods of rare events, revealing that historically rare 1-in-50-year and 1-in-100-year precipitation extremes are projected to occur more frequently by the end of the century. These findings highlight a profound shift in hydrological risk for African societies, with implications for flood frequency and severity, agricultural productivity and food security, hydropower reliability, and broader socioeconomic stability.

We used a physical-scaling diagnostic to decompose changes in extreme precipitation into thermodynamic (moisture-related) and dynamic (circulation-related) components^31,68^. While previous studies often attribute intensification primarily to CC scaling, our analysis advances this understanding by combining precipitation-scaling diagnostics with explicit surface and thermodynamic energy-budget analyses to identify the physical processes that amplify thermodynamic moistening and modulate projected changes in vertical motion across African subregions. Our results reveal that thermodynamic processes are the dominant driver, consistent with theoretical expectations from the CC relationship. Warming-induced increases in saturation specific humidity strengthen moisture availability for extreme precipitation, producing spatially uniform patterns of intensification that closely match Rx1day changes. The strong correlation between changes in saturation specific humidity and changes in near-surface temperature highlights the tight coupling between atmospheric warming and precipitation extremes across the continent. Diagnostics of the surface energy budget further reveal that the projected increase in net downward radiation, due primarily to enhanced greenhouse forcing, is the main driver of the projected warming, with latent heat flux partially offsetting radiative warming in regions with high moisture availability. In addition, analysis of the thermodynamic energy equation further highlights the role of diabatic heating and associated vertical motion in modulating regional responses, providing a process-level explanation for why thermodynamic amplification manifests differently across African subregions. Furthermore, we find that dynamic contributions exhibit substantial regional contrasts: reduced upward motion contributes to decreases in extreme precipitation in parts of subtropical southern Africa and the northern Sahara, whereas enhanced upward motion driven by increased diabatic heating reinforces thermodynamic amplification in equatorial regions. Although the dynamic response strengthens under SSP5-8.5, except in WSAF, ESAF, and MDG (Supplementary Fig. S1), it generally does not offset thermodynamic intensification. Instead, the interplay between thermodynamic moistening and dynamic anomalies shapes regional heterogeneity in Rx1day responses, particularly over WAF, CAF, and NEAF, where dynamics become increasingly influential.

Despite the robust Rx1day intensification, substantial regionally heterogeneous uncertainties persist. To better understand these sources of projection uncertainties, we explicitly decompose projection variance into contributions from model uncertainty, scenario uncertainty, and internal variability, an analysis that has rarely been conducted specifically for African precipitation extremes at subregional levels. While most models agree on the direction of change in Rx1day and associated TH and DY components, noticeable variations exist in magnitude. Variance decomposition shows that model uncertainty dominates Rx1day projections by the end of the century, accounting for more than 70% of total variance across Africa and exceeding 80% in Madagascar. These contributions, which are dominated by dynamic processes, likely reflect both structural differences in how CMIP6 models represent subgrid-scale physical processes, such as deep convection, cloud microphysics, and boundary-layer turbulence as well as differences in the simulation of regional circulation patterns that regulate large-scale ascent and moisture transport. These factors have remained major sources of uncertainty in tropical precipitation projections^34,95–97^. Uncertainty from internal variability decreases with lead time, contributing less to total variance toward the end of the century. While scenario uncertainties emerge as we progress toward the end of the century, they emerge relatively earlier than in mean precipitation projections^34^, becoming the second-largest contributor by mid-century and reaching up to ~25% of total variance in the Sahara. We find that thermodynamic processes largely drive scenario uncertainty, emphasizing the role of future warming levels in shaping extreme precipitation risk.

One critical question is whether the uncertainty in projected Rx1day can be meaningfully reduced through the well-established observational emergent-constraint relationship. Applying a hierarchical emergent constraint (HEC)^64,77,83,84^ based on observed historical global mean surface temperature trends offers a powerful means to reduce subregional uncertainties and refine projections. We find a robust relationship between historical GMST trends and projected Rx1day increases across most African subregions, with correlations up to 0.6 in SEAF and ESAF under SSP5-8.5. Models with stronger historical warming tend to project larger extreme precipitation increases, consistent with recent global studies^64,77,83^. We note that this constraint specifically targets the thermodynamic component of precipitation extremes, which is closely tied to global moisture availability and CC scaling. While dynamic contributions dominate intermodel variance, constraining using historical circulation trends is challenging because regional dynamic processes over Africa are highly heterogeneous, observationally uncertain, and often dominated by internal variability. For example, historical trends in 500-hPa vertical velocity correlate only weakly with projected changes in the dynamic contribution (Supplementary Fig. S27), indicating insufficient predictive power for an emergent constraint. This complexity is illustrated in the Sahel, where wetter conditions have traditionally been linked to a strengthened Tropical Easterly Jet (TEJ) that enhances upper-level divergence and deep convection^98^, yet future projections show increased precipitation despite a weakening TEJ, suggesting a reduced role of dynamic controls relative to thermodynamic forcing under warming^99^. Nonetheless, although the HEC framework primarily constrains the thermodynamic contribution to precipitation extremes through large-scale atmospheric moistening, a dominant driver of Rx1day intensification under warming, it may also indirectly reduce some dynamic uncertainty, because the diabatic heating that drives vertical motion depends partly on moisture availability, which is itself constrained by GMST. Implementing this constraint substantially reduces projection uncertainty, with the highest reductions observed in regions where thermodynamic processes dominate (e.g., CAF and SEAF), with relative reductions in variance exceeding 30–35%. Constrained Rx1day projections are consistently weaker in magnitude than unconstrained ones, particularly under SSP5-8.5, suggesting that CMIP6 models tend to overestimate historical warming. This effect is also reflected in the projected magnitude of change, where the Africa-wide increase in Rx1day decreases from about 15% to 9% under SSP2-4.5 and from about 32% to 19% under SSP5-8.5 after applying the constraint. The largest increases persist in northeastern Africa, where unconstrained projections reach roughly 56% under SSP5-8.5 but are reduced to about 34% after applying the constraint. Even in regions where the HEC relationship is relatively weaker, the constraint still narrows the distribution while producing physically plausible projections. Across most regions the constraint therefore both reduces variance and lowers the projected relative change, indicating that unconstrained ensembles tend to produce stronger increases in extreme precipitation. Furthermore, analysis at 1.5 °C and 2 °C global warming levels shows that the HEC consistently reduces intermodel spread, with slightly stronger reductions at 2 °C. Relative reductions in variance reach 7.4% over SAH and 12.1% over SEAF, with the largest reduction (28.5%) over MED at 2.0 °C under SSP5-8.5. Although RRVs are generally modest (typically a few percent to ~10%), these reductions reinforce the benefit of emergent constraints for increasing confidence in regional hydrological-extreme projections. A key limitation of the HEC applied here is that it primarily constrains the thermodynamic contribution to precipitation extremes through large-scale atmospheric moistening, as represented by its relationship with GMST. It does not directly constrain uncertainties associated with local surface conditions or subregional dynamic processes that are important for triggering convection and its subsequent development. In addition, the constraint is derived from the CMIP6 ensemble, in which convection is parameterized rather than explicitly resolved. As a result, systematic biases in representing convective variability and precipitation extremes may not be corrected by the constraint, and the constrained projections may therefore represent a conservative estimate of future change. Although the constraint reduces intermodel spread and improves consistency with observed large-scale warming, the constrained magnitudes of Rx1day change should be interpreted with caution.

The implications of these constrained and unconstrained projections for African populations and economies are substantial. By linking physically constrained projections of extreme precipitation to demographic and economic datasets, this study provides a direct translation of climate extremes into societal exposure, an aspect often missing from purely physical analyses of precipitation change. Our results further show that this dominance is driven primarily by socioeconomic factors: decomposition analysis indicates that although Rx1day increases substantially, the projected changes in population (or GDP) are even more pronounced. Consequently, socioeconomic growth accounts for the largest share of the increase in exposure, whereas the isolated impact of Rx1day intensification remains comparatively minor. In general, urban exposure dominates total exposure trends due to rapid urbanization and high population densities, particularly in WAF, where both demographic and climatic drivers are expected to amplify flood risk. Because urban population growth and economic concentration are large and positive across most regions, urban exposure increases substantially, whereas rural exposure changes remain comparatively small. Consequently, even where rural exposure declines (SSP5-8.5), the magnitude of increases in urban exposure overwhelmingly controls total exposure trends across Africa. Similarly, GDP exposure rises across all regions, with the strongest increases in economically dynamic and densely populated areas, such as WAF and CAF. Constraints reduce exposure by roughly 5–25% across subregions, but the direction of change remains unchanged. This highlights that even after accounting for observationally consistent model behavior, Africa faces a future of significantly heightened hydrological risk, driven by climate change, population growth, and economic expansion.

It is important to note that all CMIP6 models used in this study rely on parameterized convection, which may influence the representation of short-duration precipitation extremes. Convection-permitting (kilometer-scale) simulations that explicitly resolve deep convective processes often produce stronger intensification of rainfall extremes compared with convection-parameterized models. For example, high-resolution simulations over Africa show that explicitly resolving convection can substantially increase projected changes in extreme precipitation intensity relative to parameterized models^46^. While such simulations provide valuable insights into convective processes, they are currently limited in spatial coverage, ensemble size, and simulation length, making them difficult to use in emergent-constraint frameworks that require large multimodel ensembles. The consistent direction of change between CMIP6 models and convection-permitting simulations, both projecting stronger precipitation extremes under warming, provides confidence in the qualitative signal reported herein. Also, the CMIP6 models used herein allow us to systematically assess model agreement and quantify uncertainties across models and scenarios in a statistically robust framework. However, because convection is not explicitly resolved in CMIP6 models, the constrained projections presented in this study should be interpreted as a likely conservative estimate, or lower bound, of future intensification of precipitation extremes across Africa. Future studies combining large-ensemble convection-permitting simulations with constraint frameworks may further refine these projections as such datasets become more widely available. Overall, this study advances the understanding of African precipitation extremes by integrating mechanistic diagnostics, uncertainty attribution, hierarchical emergent constraints, and socioeconomic exposure analysis within a single framework. Robust intensification of extreme precipitation is likely unavoidable across much of Africa, even under moderate emissions pathways. By bridging the gap between global thermodynamic theory and regionally constrained climate projections, the results provide a more physically grounded and policy-relevant assessment of future hydroclimate risk across African subregions. Reducing future risks will therefore require strategically integrating these scientific findings into socioeconomic planning.

## Methods

### CMIP6 Simulations

This study analyzes daily data from 18 CMIP6 models in historical simulations and future projections under the SSP2-4.5 and SSP5-8.5 scenarios^100,101^. Detailed information on the selected models, including their originating institutions and native grid spacing, is provided in Supplementary Table S1. We computed the annual maximum daily precipitation (Rx1day) from daily precipitation and analyzed near-surface air temperature, surface pressure, specific humidity, atmospheric temperature, and horizontal and vertical wind components across pressure levels. Besides these variables, we also analyzed the following variables at monthly resolution: surface temperature, near-surface air temperature, latent and sensible heat fluxes, upwelling and downwelling shortwave and longwave radiation under both clear-sky and all-sky conditions, geopotential height at 500 hPa, and total precipitable water. To ensure consistency across models and enable direct intercomparison, all variables were interpolated to a common horizontal resolution of 2.81° × 2.81°, corresponding to the coarsest model grid, using first-order conservative interpolation. For regional analyses, we focus on nine subregions based on the IPCC AR6 WGI regional classification^102^. These include eight African subregions: Sahara (SAH), Western Africa (WAF), Central Africa (CAF), North Eastern Africa (NEAF), South Eastern Africa (SEAF), East Southern Africa (ESAF), West Southern Africa (WSAF), and Madagascar (MDG). In addition, we include the Mediterranean (MED) region from the IPCC AR6 classification; however, only the portion of MED that overlaps with Africa is considered in this analysis. The historical baseline period is 1985–2014, and the late 21st-century period is 2070–2099.

### Observations

Previous studies have documented substantial differences among gridded observational precipitation products due to variations in data sources and processing methods^103–105^. To account for these uncertainties, for the 1985–2014 period, model-computed Rx1day is evaluated against multiple independent observational datasets, including: (i) the Climate Hazards Group Infrared Precipitation with Stations (CHIRPS) at 0.05° resolution^106^; (ii) the Global Precipitation Climatology Center (GPCC) full daily version 2018 at 1° resolution^107^; (iii) the CPC unified gauge-based daily precipitation analysis at 0.5° resolution^108^; and (iv) the NOAA CPC FEWS African Rainfall Climatology (ARC2) at 0.1° resolution^109^. Observed temperature trends are derived from the HadCRUT^87^ and GISTEMP4^88^ records. Observed GMST combines land–ice surface air temperatures with sea-surface temperatures, differing from the GMST definition in model output. To address this mismatch, the HadCRUT4 trend is scaled by a factor of 1.074^110^, and 0.014 °C per decade is added to the GISTEMP4 trend following Tokarska et al. (2020)^83^. This correction is not applied to regional trends shown in the corresponding figure (Supplementary Fig. S22).

### Exposure of population and GDP

The NASA Socioeconomic Data and Applications Center (SEDAC) provides projected gridded population data following SSP pathways at 1/8° resolution, with the year 2000 as the baseline^111^. Similarly, Murakami et al. (2021)^112^ provide the projected GDP across SSP scenarios at 1/12° resolution. Using these datasets, we evaluate changes in population and GDP exposure for each grid cell and the relative importance of different factors in driving these changes^113,114^. These factors include the climate, population/GDP, and interaction effects.

1 $$\Delta {{Exposure}}_{{Pop-Rx1day}}=	 \overline{{Pop}}\times \Delta {Rx1day}+\Delta {Pop}\times \overline{{Rx1day}} \\ 	+\Delta {Pop}\times \Delta {Rx1day}$$

2 $$\Delta {{Exposure}}_{{GDP-Rx1day}}=	 \overline{{GDP}}\times \Delta {Rx1day}+\Delta {GDP}\times \overline{{Rx1day}} \\ 	+\Delta {GDP}\times \Delta {Rx1day}$$

Here, Δ represents the mean change between the future period (2070–2099) and the historical period (1985–2014), and the overbar represents the mean during the historical period. For the population/GDP, the year 2000 was chosen as the historical period rather than a multiyear mean because socioeconomic data are available at decadal intervals, and the 2010 data are projections under the SSP scenarios rather than observations. The climate effect is defined as the impact of the Rx1day change on the historical population/GDP (1^st^ term in the equation). The population/GDP effect is the impact of changes in population/GDP relative to historical Rx1day (2^nd^ term). The interaction effect accounts for changes in both population/GDP and Rx1day (3^rd^ term).

### Probability ratio

The probability ratio (PR), defined as the change in the probability of extreme events between a future and a historical period^115^, is used here to evaluate how the frequency of rare events evolves under climate change. To identify the threshold for rare extremes, we fitted both the generalized extreme value (GEV) distribution and the Gumbel extreme value distribution to historical Rx1day data independently, to characterize the temporal distribution of extremes at specified return periods^116–118^. The Gumbel distribution is a special case of the GEV with a fixed (zero) shape parameter and cannot represent heavy- or bounded-tail behavior, which can bias high-return-level estimates if the true tail deviates from exponential decay. For the more flexible GEV, estimation of the shape parameter, which governs tail behavior, is often highly uncertain in finite samples, especially for rare return periods, making return-level and exceedance-probability estimates sensitive to sample size and fitting uncertainty^119,120^. We focused on 50-year and 100-year return periods over the historical baseline period (1985–2014), where the historical probabilities of occurrence, *P*_*0*_, are 2% and 1%, respectively. The fitted distributions provide the Rx1day thresholds associated with these return periods, which are then applied to the future period (2070–2099) to determine the exceedance probability under projected climate conditions, *P*_*1*_. PR is then computed as the ratio *P*_*1*_*/P*_*0*_, quantifying how much the likelihood of these rare events increases or decreases relative to the historical baseline. Therefore, PR values quantify the amplification of historically rare events; for the 50-year and 100-year return periods considered here, PR can reach values of approximately 50 and 100, respectively, if future probabilities approach 1, though in general PR has no strict upper limit.

To quantify statistical uncertainty in PR estimates and assess where changes are robust, we applied a parametric bootstrap procedure independently to the Gumbel and GEV fits for each grid cell and climate model^121,122^. For each grid cell, extreme value distribution (Gumbel or GEV) was first fitted to the historical Rx1day by maximum likelihood estimation, with the GEV shape parameter constrained to a physically plausible range of 0.8 to exclude unrealistically heavy or bounded tails^123,124^. For each return period (50 and 100 years), we performed a parametric bootstrap by generating 1000 synthetic samples from the fitted Gumbel or GEV distribution using the estimated parameters from the historical period. Each synthetic sample matched the original sample size, and the distribution was refitted to recompute the corresponding threshold. Each bootstrap threshold was applied to the original (non‑resampled) future Rx1day series to obtain a bootstrap replica of the exceedance probability and thus of PR, so that uncertainty reflects only the sampling variability in estimating the historical extreme‑value distribution, consistent with standard practice for parametric bootstrap inference in extreme‑value analysis^125^. The resulting empirical distribution of PR values was used to construct two‑sided 95% confidence intervals as the 2.5th and 97.5th percentiles; PR was classified as statistically significant where the confidence interval excluded unity (lower bound > 1 or upper bound <1)^126,127^, and intervals were reported only when at least 100 valid bootstrap replicates were available for a given grid cell and return period.

### Thermodynamic and dynamic decomposition

To quantify the thermodynamic (TH) and dynamic (DY) contributions to projected changes in precipitation extremes, we applied the physical-scaling diagnostic^31,68^. This diagnostic provides a physically consistent framework for linking extreme precipitation intensity to large-scale atmospheric thermodynamics and dynamics. At each grid point and for each model simulation, the precipitation associated with daily extremes (denoted as *P*_*e*_) is approximated by the vertically integrated condensation rate, expressed as:

3 $${P}_{e} \sim -\left\{{\omega \,}_{e}{\frac{{{dq}}_{s}}{{dp}}|}{{\theta }^{*}}\right\}$$

where ω_e_ is the vertical velocity (Pa s^−1^) corresponding to the occurrence of precipitation extremes, *q*_*s*_ is the saturation specific humidity on the day of the extreme event, and ${\frac{{{dq}}_{s}}{dp}}|{{\theta }^{*}}$ is the vertical derivative of *q*_*s*_ at constant saturation equivalent potential temperature (θ∗). Curly braces {⋅} indicate a mass-weighted vertical integration over all ascending layers in the troposphere. The troposphere is defined as all pressure levels from the surface up to the level with a lapse rate greater than 2 K km^−1^ and below 50 hPa; *q*_*s*_ is computed using a modified Tetens formula^128^, which employs a quadratic interpolation between saturation over ice and liquid water for temperatures between 250 K and 273 K. This formulation provides a more accurate representation of the potential thermodynamic contribution associated with atmospheric temperature variations than using simulated daily mean specific humidity.

To isolate the individual effects of thermodynamic and dynamic processes on projected precipitation extremes, we decomposed total changes in *P*_*e*_ between historical and future climates into contributions from thermodynamic (TH), dynamic (DY), and residual terms (RES):

4 $${\delta P}_{e} \sim -\delta \left\{{\omega \,}_{e}{\frac{{{dq}}_{s}}{{dp}}|}_{{\theta }^{*}}\right\}=\delta {TH}+\delta {DY}+{RES}$$

where $\delta$(⋅) denotes the difference between the future (2070–2099) and historical (1985–2014) periods. The thermodynamic effect, $\delta {TH},$ caused by changes in saturation specific humidity, is defined as:

5 $$\delta {TH}=-\left\{{{\omega }_{e}}^{{his}}{\left({\frac{{{dq}}_{s}}{{dp}}|}_{{\theta }^{*}}\right)}^{{fut}}\right\}+\left\{{{\omega }_{e}}^{{his}}{\left({\frac{{{dq}}_{s}}{{dp}}|}_{{\theta }^{*}}\right)}^{{his}}\right\}$$

and the dynamic effect, *δDY*, due to changes in vertical velocity, is defined as:

6 $$\delta {DY}=-\left\{{{\omega }_{e}}^{{fut}}{\left({\frac{{{dq}}_{s}}{{dp}}|}_{{\theta }^{*}}\right)}^{{his}}\right\}+\left\{{{\omega }_{e}}^{{his}}{\left({\frac{{{dq}}_{s}}{{dp}}|}_{{\theta }^{*}}\right)}^{{his}}\right\}$$

where superscripts *his* and *fut* denote the historical and future climatological states, respectively.

In contrast to Pfahl et al. (2017)^31^, we compute the DY contribution separately rather than estimating it as the residual between the full scaling (Eq. 3) and the TH contribution, thereby avoiding the inclusion of nonlinear variations and transient eddy effects in the DY term. The RES term, representing the difference between the full scaling and the sum of TH and DY, is generally small in projections^31,76,77^ and also evident over Africa (Supplementary Fig. S9), and it is thus neglected herein.

The diagnostic was computed at each model grid cell using daily data. To avoid spurious percentage anomalies, grid points with weak climatological mean extreme precipitation or scaling amplitude (<0.2 mm day^−1^) during 1985–2014 were excluded, following Pfahl et al. (2017)^31^. For each grid cell, the day of maximum daily precipitation (Rx1day) within each year was identified, and corresponding ω_e_ and temperature (used to derive *q*_*s*_) profiles were extracted for analysis. By holding either the vertical motion or the thermodynamic structure constant, this scaling framework quantitatively separates the extent to which future changes in extreme precipitation are driven by enhanced moisture availability (thermodynamics) versus intensified or weakened vertical motion (dynamics). The decomposition allows for a physically interpretable assessment of projected changes in Rx1day across models and regions.

### Surface energy budget

The near-surface temperature decomposition was done using the surface energy budget following the method proposed by Lu and Cai (2009)^129^ (Eqs. 7–11). This framework, which has been applied in several previous studies^23,130,131^, allows us to isolate the contribution of individual energy budget components to changes in near-surface temperature.

7 $$\Delta {TAS}\,=\frac{\Delta {R}^{\downarrow }-\,\Delta L-\Delta H-\Delta Q}{4\sigma \bar{{T}_{s}}}+{Res}$$

8 $$\Delta {Q}^{\downarrow }=\Delta {{LW}}^{\downarrow }-\Delta {{LW}}^{\uparrow }+\Delta {{SW}}^{\downarrow }-\Delta {{SW}}^{\uparrow }-\Delta H-\Delta L$$

Here, Δ refers to the change between the future period (2070-2099) and historical period (1985-2014), and the overbar denotes the historical mean. *R↓* is the net downwelling surface radiation reaching the surface. *L* and *H* are the upward latent and sensible heat flux, respectively. *Q* is the net heat storage, representing energy transferred to or from the Earth’s surface. *LW* and *SW* represent longwave and shortwave radiation, respectively; and the arrows (↓ or ↑) indicate downwelling or upwelling components. *TAS* and *TS* are near-surface air temperature and surface temperature, respectively, while σ is the Stefan–Boltzmann constant. *Res* is the residual term denoting the difference between near-surface temperature and surface temperature.

The change in net downwelling surface radiation can be further decomposed into contributions from clear-sky conditions, surface albedo feedback (SAF), and cloud radiative forcing (CRF), using Eqs. 9–11:

9 $$\Delta {R}^{\downarrow }=\Delta {{LW}}^{{cs}\downarrow }+\Delta {{SW}}^{{cs}\downarrow }+\Delta {SAF}+\Delta {CRF}$$

10 $$\Delta {SAF}=-(\Delta {{SW}}^{{as}\downarrow }+\,\overline{{{SW}}^{{as}\downarrow }})\Delta \alpha$$

11 $$\Delta {CRF}=(1-\bar{\alpha })\Delta {{SW}}^{{cl}\downarrow }+\Delta {{LW}}^{{cl}\downarrow }$$

Here, the superscripts *as*, *cs*, and *cl* denote all-sky, clear-sky, and cloud components, respectively. α is the surface albedo, computed as the ratio of outgoing shortwave radiation to incoming shortwave radiation at the surface.

### Thermodynamic energy equation

We diagnose the processes modulating projected changes in vertical motion using the thermodynamic energy budget. Under hydrostatic balance, the thermodynamic energy equation in pressure coordinates^132^ is written as

12 $$\frac{\partial T}{\partial t}=-{V}_{h}\cdot \nabla T+{S}_{p}\omega+\frac{{Q}_{1}}{{C}_{p}}$$

where *T* denotes temperature (K), *Vₕ* is the horizontal wind (m s⁻¹), $\omega$ is the pressure vertical velocity (Pa s⁻¹), *Cₚ* is the specific heat of dry air at constant pressure (1004.8 J kg⁻¹ K⁻¹), and *Q₁* is the net diabatic heating rate (K s⁻¹). The term *Sₚ* represents static stability and is calculated as: ${S}_{p}\,=-\,\frac{T}{\theta }\frac{\partial \theta }{\partial p}\,$(${{K\; Pa}}^{-1}$)^133^, where $\theta$ is the potential temperature (K). Under conditions where stability varies little with height, the contribution from vertical temperature advection is governed mainly by the ω term^134–136^, and θ is the potential temperature in kelvins. In Eq. 12, the term on the left-hand side, $\frac{\partial T}{\partial t}$, denotes the local temperature tendency. The three terms on the right-hand side describe the processes governing this tendency. The first term, $-{V}_{h}\cdot \nabla T,$ represents horizontal temperature advection, i.e., the lateral transport of heat by the large-scale circulation. The second term, ${S}_{p}\omega,$ represents temperature changes due to vertical advection, such that upward motion (ω < 0) leads to adiabatic cooling and downward motion (ω > 0) leads to adiabatic warming, with the magnitude of the response modulated by the static stability *Sₚ*. The third term, $\frac{{Q}_{1}}{{C}_{p}}$, represents diabatic heating, i.e., net non-adiabatic heating of the column arising from processes, such as latent heat release, radiation, and sensible heat fluxes.

### Emergent constraints on projected extreme precipitation

To constrain the projected Rx1day over Africa, we apply the HEC framework proposed by Bowman et al. (2018)^58^ and widely used in recent studies. The HEC framework builds on the concept of emergent constraints, which uses physically interpretable relationships across climate models between an observable aspect of the present-day climate and a future climate response. In this study, we use the historical global mean surface temperature (GMST) trend as the predictor variable because it captures intermodel differences in the large-scale thermodynamic response to warming, which influences precipitation extremes. The HEC approach provides a Bayesian formulation that accounts for uncertainties in both model simulations and observations. Unlike classical emergent-constraint methods based on simple regression, the hierarchical framework explicitly incorporates observational uncertainty and the strength of the intermodel relationship between the predictor and projected response. Under this framework, the constrained projections represent a probabilistic estimate of the future response conditioned on the observed value of the predictor variable.

Following Bowman et al. (2018)^58^, constrained projections of extreme precipitation changes are assumed to follow a Gaussian distribution, characterized by a mean and variance. This assumption provides a good approximation for describing the intermodel spread, although deviations from normality may occur in some ensembles.

The mean of the constrained future projections is defined as:

13 $${\mu }_{z|y}={\mu }_{z}+\frac{\rho {\sigma }_{z}{\sigma }_{x}}{{\sigma }_{x}^{2}+{\sigma }_{y}^{2}}\left({\mu }_{y}-{\mu }_{x}\right)$$

where *x* and *z* denote the historical simulations and future projections, respectively, and *y* denotes observations. The symbols μ and σ refer to the mean and standard deviations, respectively, and *ρ* is the intermodel correlation coefficient between *x* and *z*.

The adjustment factor $\frac{\rho {\sigma }_{z}{\sigma }_{x}}{{\sigma }_{x}^{2}+{\sigma }_{y}^{2}}$ determines how strongly observational information modifies the unconstrained model mean. This factor increases when the intermodel correlation between the predictor and predictand is strong and when observational uncertainty is small relative to the spread of model simulations. Conversely, when observational uncertainty is large or the intermodel relationship is weak, the adjustment factor becomes small, and the constrained projection approaches the original multimodel mean.

The variance of the constrained future projections is computed as:

14 $${\sigma }_{{z|y}}^{2}=\left(1-\frac{{\rho }^{2}}{1+{\left(\frac{{\sigma }_{x}^{2}}{{\sigma }_{y}^{2}}\right)}^{-1}}\right){\sigma }_{z}^{2}$$

which reflects the reduction in projection uncertainty obtained by conditioning on observational information. Both unconstrained and constrained projections are assumed to be approximately Gaussian. The relative reduction of variance (RRV) is then calculated as:

15 $${RRV}=\left(1-\frac{{\sigma }_{z|y}^{2}}{{\sigma }_{z}^{2}}\right)\times 100\%$$

This metric quantifies the extent to which observational information reduces projection uncertainty.

The HEC framework relies on several key assumptions. First, it assumes an approximately linear relationship across models between the historical predictor and the future response. Second, uncertainties are assumed to be approximately Gaussian, allowing the constrained distribution to be described by its mean and variance. Third, the observational estimate is assumed to be an unbiased representation of the true climate state. Deviations from these assumptions, including nonlinear predictor–predictand relationships, structural model errors, or underestimated observational uncertainties, may influence the strength of the constraint. Therefore, the robustness of the emergent constraint depends on the physical plausibility of the predictor–predictand relationship and the reliability of the observational dataset used to constrain the projections.

### Sources of projection uncertainty

There are three primary sources of uncertainty in climate projections, as reported in previous studies^5,137–140^: natural internal variability, structural differences among models, and the unpredictability of future greenhouse gas and aerosol emissions. We quantify these uncertainties following the framework of Hawkins and Sutton (2009, 2011)^137,138^, and the procedure outlined in Akinsanola et al. (2024; see their Supporting Information, Eq. 1–6)^5^. For each model and scenario, the time series of Rx1day is fitted with a smooth fourth-order polynomial, and deviations from this fit are taken as residuals. These residuals are attributed to natural internal variability arising from intrinsic fluctuations in the climate system. Internal variability is estimated from the multimodel mean of the variance of residuals across scenarios and time. Model uncertainty is defined as the multiscenario mean of the variance of the smooth fit across models. Scenario uncertainty is defined as the variance of the multimodel and multiscenario mean smooth fit. Assuming model uncertainty, scenario uncertainty, and internal variability are independent of each other, the total uncertainty can be expressed as the sum of these three components. The relative contribution of each component is computed as its fraction of the total variance in projected Rx1day changes. This decomposition provides a quantitative framework for assessing how the importance of different sources of uncertainty evolves throughout the 21st century.

### Reporting summary

Further information on research design is available in the Nature Portfolio Reporting Summary linked to this article.

## Supplementary information

Supplementary Information

Reporting Summary

Transparent Peer Review file

## Data Availability

All datasets used in this study are publicly and freely available. CMIP6 data are publicly available through the Earth System Grid Federation at http://esgf.llnl.gov/. The Global Precipitation Climatology Center (GPCC) dataset was retrieved from https://psl.noaa.gov/data/gridded/data.gpcc.html/. Climate Hazards Group InfraRed Precipitation with Stations (CHIRPS) products can be downloaded from https://www.chc.ucsb.edu/data/. The CPC Global Unified Gauge-Based Analysis of Daily Precipitation (CPC-Unified) is available at https://psl.noaa.gov/data/gridded/data.cpc.globalprecip.html/. The African Rainfall Climatology (ARC) dataset can be accessed at https://iridl.ldeo.columbia.edu/SOURCES/.NOAA/.NCEP/.CPC/.FEWS/.Africa/.DAILY/.ARC2/.daily/index.html?Set-Language=en/. Observational temperature data are available at https://www.metoffice.gov.uk/hadobs/hadcrut4/ for HadCRUT4 and https://data.giss.nasa.gov/gistemp/ for GISTEMP4. Projected gridded population data are provided by the NASA Socioeconomic Data and Applications Center [10.7927/M30P-J498]^141^, and projected gridded GDP data are available at 10.6084/M9.FIGSHARE.12016506^142^. The processed data are made available in an open-access Zenodo repository [10.5281/zenodo.19053148]^143^, with all raw inputs accessible via the sources listed above.

## Code availability

All analyses and figures were produced using Python (https://www.python.org/). All code required to reproduce the figures in this study is publicly available in an open-access Zenodo repository [10.5281/zenodo.19053148]^143^.
