# Supplementary material for: Robust intensification of projected regional precipitation extremes over Africa
Source: Nat Commun. 2026 May 16;17:6492. doi: 10.1038/s41467-026-73246-2 (PMC13377057; doi:10.1038/s41467-026-73246-2)
Supplement: Supplementary file 1 — Supplementary Information [file 41467_2026_73246_MOESM1_ESM.pdf]

Supplementary Information for

Robust intensification of projected regional precipitation extremes over Africa

Akintomide A. Akinsanola*, Thierry N. Taguela, and Vishal Bobde

Department of Earth and Environmental Sciences, University of Illinois Chicago, IL, USA
*Corresponding author: Akintomide A. Akinsanola
Email: aakinsan@uic.edu

Contents of this file

Table S1–S3

Figures S1–S18

Table S1. Information on the 18 CMIP6 models used in this study.

Model	Institute	Resolution	Reference
ACCESS-CM2	Commonwealth Scientific and Industrial Research Organisation	1.88° × 1.25°	Dix et al. (2019)[1-3]
BCC-CSM2-MR	Beijing Climate Center (BCC)	1.125°×1.125°	Wu et al., (2018)[4]; Xin et al., (2019)[5,6]
CanESM5	Canadian Earth System Model	2.81° × 2.81°	Swart et al., (2019)[7-9]
CESM2-WACCM	Climate and Global Dynamics Laboratory, Boulder, USA.	0.9° × 1.25°	Danabasoglu et al., (2019)[10-12]
CMCC-CM2-SR5	Euro-Mediterranean Center on Climate Change, Italy	0.94° × 1.25°	Lovato & Peano, (2020)[13-15]
CMCC-ESM2	Euro-Mediterranean Center on Climate Change, Italy	0.94° × 1.25°	Lovato et al., (2021)[16-18]
EC-Earth3	EC-EARTH consortium	0.70° × 0.70°	EC-Earth, (2019)[19-21]
INM-CM4-8	Institute for Numerical Mathematics	2° × 1.5°	Volodin et al., (2019)[22-24]
INM-CM5-0	Institute for Numerical Mathematics	2° × 1.5°	Volodin et al., (2019)[25-27]
IPSL-CM6A-LR	Institute Pierre-Simon Laplace (IPSL)	2.5° × 1.26°	Boucher et al., (2018,2019)[28-30]
MIROC6	Japan Agency for Marine-Earth Science and Technology, Atmosphere and Ocean Research Institute (The University of Tokyo), and National Institute for Environmental Studies	1.41° × 1.41°	Tatebe et al., (2018)[31]; Shiogama et al., (2019)[32,33]
MPI-ESM1-2-HR	Max Planck Institute	0.94° × 0.94°	Jungclaus et al., (2019)[34]; Schupfner et al., (2019)[35,36]

MPI-ESM1-2-LR	Max Planck Institute	1.88° × 1.88°	Wieners et al., (2019)[37-39]
MRI-ESM2-0	Meteorological Research Institute (MRI)	1.13° × 1.13°	Yukimoto et al., (2019)[40-42]
NESM3	Nanjing University of Information Science and Technology (NUIST), China.	1.88° × 1.88°	Cao & Wang, (2019)[43]; Cao et al., (2019)[44,45]
NorESM2-LM	Norwegian Climate Centre	1.875° × 2.5°	Seland et al., (2019)[46-48]
NorESM2-MM	Norwegian Climate Centre	0.94° × 1.25°	Bentsen et al., (2019)[49-51]
TaiESM1	Research Center for Environmental Changes (RCEC), Academia Sinica, Nankang, Taipei, Taiwan.	0.94° × 1.25°	Lee & Liang, (2020)[52-54]

Table S2. Periods in which each CMIP6 model reaches 1.5 °C and 2.0 °C global warming levels (GWLs) under the SSP2-4.5 and SSP5-8.5 scenarios[55].

Model Name	SSP2-4.5		SSP5-8.5	
	**GWL 1.5°C**	**GWL 2.0°C**	**GWL 1.5°C**	**GWL 2.0°C**
ACCESS-CM2	2019-2038	2031-2050	2016/2035	2029-2048
BCC-CSM2-MR	2026-2045	2048-2067	2021-2040	2034-2053
CanESM5	2004-2023	2015-2034	2003-2022	2013-2032
CESM2-WACCM	2015-2034	2030-2049	2011-2030	2024-2043
CMCC-CM2-SR5	2016-2035	2029-2048	2012-2031	2024-2043
CMCC-ESM2	2021-2040	2031-2050	2020-2039	2030-2049
EC-Earth3	2013-2032	2035-2054	2015-2034	2026-2045
INM-CM4-8	2026-2045	2054-2073	2021-2040	2037-2056
INM-CM5-0	2028-2047	2063-2082	2021-2040	2037-2056
IPSL-CM6A-LR	2009-2028	2024-2043	2009-2028	2025-2044
MIROC6	2037-2056	2064-2083	2031-2050	2044-2063
MPI-ESM1-2-HR	2028-2047	2054-2073	2024-2043	2040-2059
MPI-ESM1-2-LR	2027-2046	2048-2067	2025-2044	2039-2058
MRI-ESM2-0	2021-2040	2040-2059	2017-2036	2029-2048
NESM3	2015-2034	2033-2052	2011-2030	2024-2043
NorESM2-LM	2046-2065	2076-2095	2033-2052	2047-2066
NorESM2-MM	2037-2056	2069-2088	2030-2049	2045-2064
TaiESM1	2022-2041	2034-2053	2019-2038	2027-2046

Table S3. EnsMean and standard deviation (in brackets) of unconstrained (U) and constrained (C) projected Rx1day at 1.5°C and 2.0°C global warming levels (GWLs) under the SSP2-4.5 and SSP5-8.5 scenarios. RRV denotes the relative reduction in variance (%).

Region Name	SSP2-4.5						SSP5-8.5					
	GWL 1.5°C			GWL 2.0°C			GWL 1.5°C			GWL 2.0°C		
	U	C	RRV	U	C	RRV	U	C	RRV	U	C	RRV
MED	-0.48 (3.94)	-3.04 (3.55)	18.7%	-0.05 (4.94)	-2.92 (4.55)	15%	2.16 (4.41)	0.78 (4.32)	4.4	-0.18 (5.81)	-4.82 (4.92)	28.5%
SAH	11.29 (11.73)	6.57 (11.30)	7.2%	16.68 (11.66)	12.03 (11.24)	7.1%	12.49 (11.21)	8.40 (10.87)	5.9%	21.23 (14.10)	15.48 (13.56)	7.4%
WAF	9.10 (5.18)	8.30 (5.16)	1%	14.86 (6.30)	13.66 (6.25)	1.6%	11.18 (5.61)	10.47 (5.59)	0.7%	17.35 (9.25)	14.89 (9.10)	3.2%
CAF	9.07 (3.96)	8.41 (3.94)	1.3%	13.59 (5.66)	12.24 (5.59)	2.5%	10.01 (4.82)	11.86 (4.66)	6.5%	16.32 (5.89)	18.29 (5.74)	5%
NEAF	9.54 (4.72)	9.29 (4.71)	0.1%	14.16 (6.66)	12.20 (6.53)	3.9%	8.96 (5.04)	9.75 (5.01)	1.1%	17.33 (7.86)	18.17 (7.84)	0.5%
SEAF	7.06 (4.09)	6.32 (4.06)	1.4%	9.96 (5.44)	7.12 (5.10)	12.1%	6.16 (4.27)	4.92 (4.19)	3.8%	11.52 (6.53)	8.98 (6.30)	6.8%
ESAF	2.89 (2.92)	3.12 (2.92)	0.3%	5.93 (4.33)	4.15 (4.16)	7.5%	3.65 (4.04)	2.90 (4.01)	1.5%	6.71 (4.10)	5.75 (4.05)	2.4%
WSAF	3.06 (2.97)	2.51 (2.95)	1.5%	3.86 (3.58)	3.48 (3.57)	0.5%	3.17 (2.91)	3.50 (2.90)	0.6%	5.57 (3.68)	6.64 (3.61)	3.8%
MDG	2.15 (3.73)	4.33 (3.44)	15.2%	4.60 (5.00)	4.33 (4.99)	0.1%	1.45 (4.18)	1.08 (4.18)	0.3%	5.02 (4.62)	4.43 (4.61)	0.7%
AFRICA	6.38 (2.67)	5.93 (2.66)	1.3%	9.95 (3.84)	8.54 (3.73)	5.9%	6.88 (3.41)	6.63 (3.40)	0.2%	11.89 (4.13)	11.56 (4.13)	0.3%

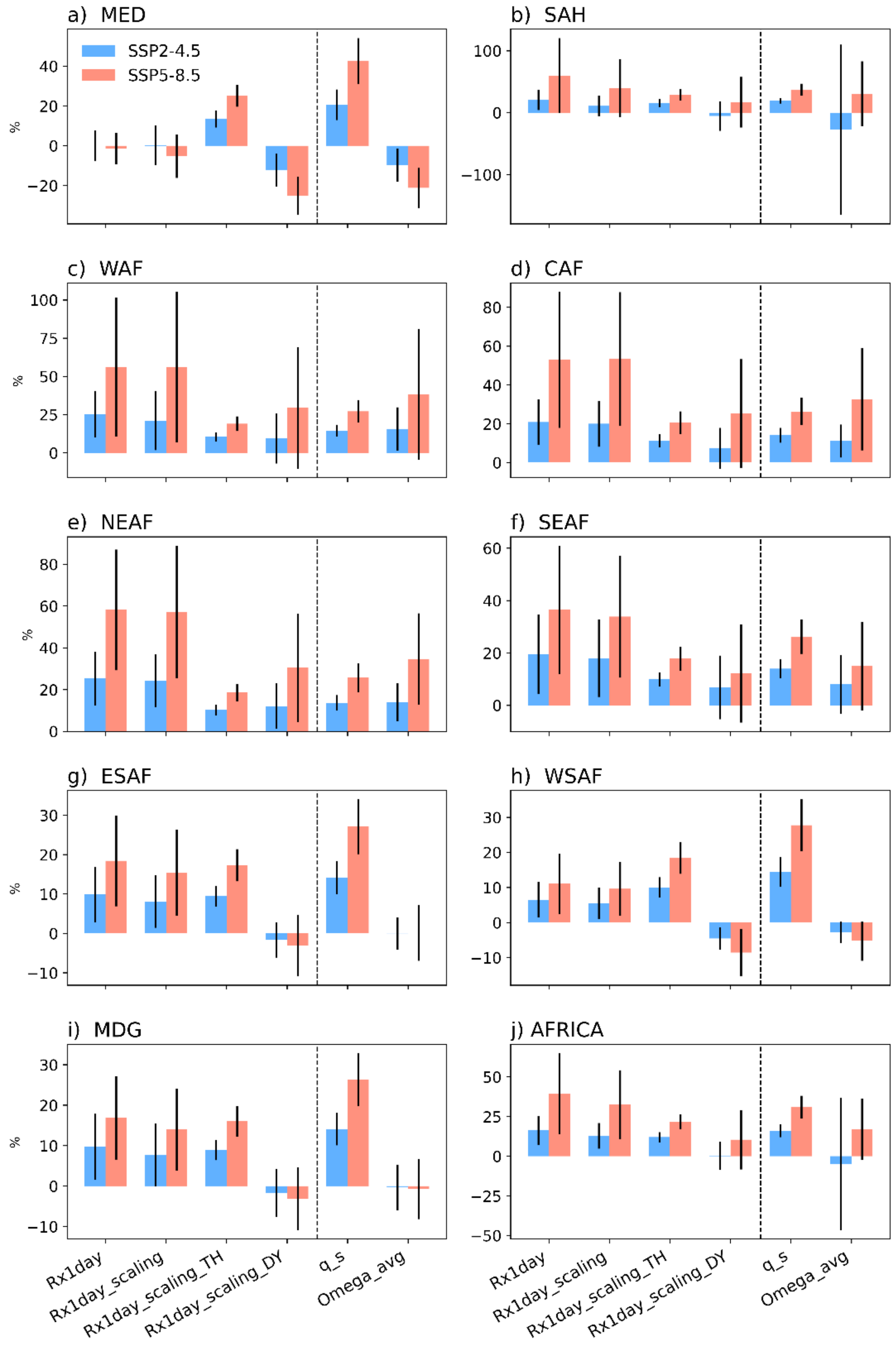

Figure S1. (a–j) Weighted area-averaged regional mean changes (%) in Rx1day, Rx1day_scaling, and individual scaling components (thermodynamic and dynamic), along with changes in vertically integrated saturation specific humidity and vertically averaged vertical velocity under the SSP2-4.5 and SSP5-8.5 scenarios. Bars represent the CMIP6 EnsMean, while vertical lines denote ±1 standard deviation, illustrating intermodel spread. Changes are calculated as the relative difference between the 2070–2099 and 1985–2014 means.

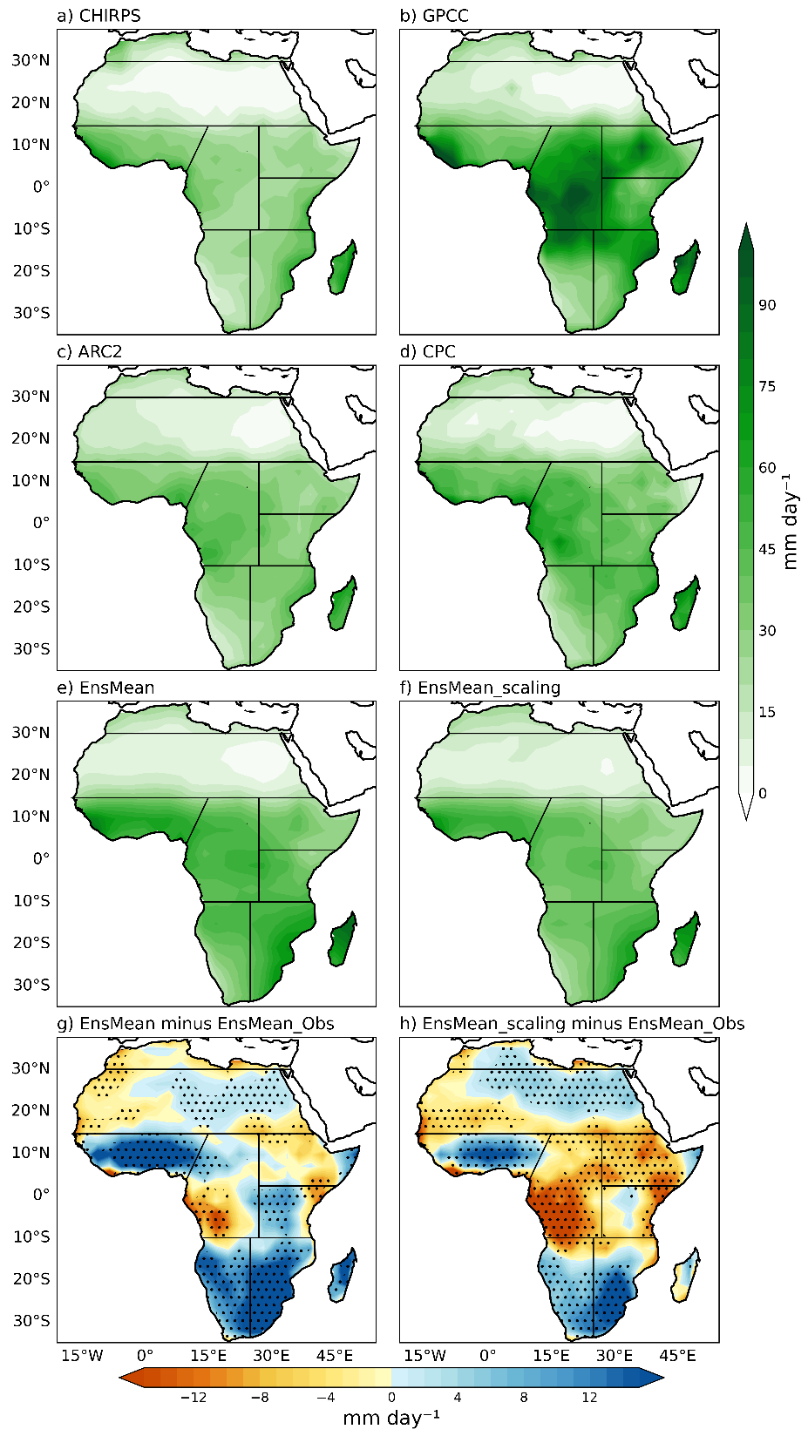

Figure S2. Mean annual maximum 1-day precipitation (Rx1day, mm/day) during the historical period (1985–2014) from (a–d) observational datasets (CHIRPS, GPCC, ARC2, and CPC), (e) the CMIP6 ensemble mean (EnsMean) Rx1day, and (f) the EnsMean computed from physical-scaling diagnostics (EnsMean_scaling). Panels (g) and (h) show the bias in EnsMean Rx1day and EnsMean Rx1day_scaling, respectively, relative to the observational mean. Stippling in (g) and (h) indicates grid points where differences are statistically significant at the 95% confidence level using a Student's t-test.

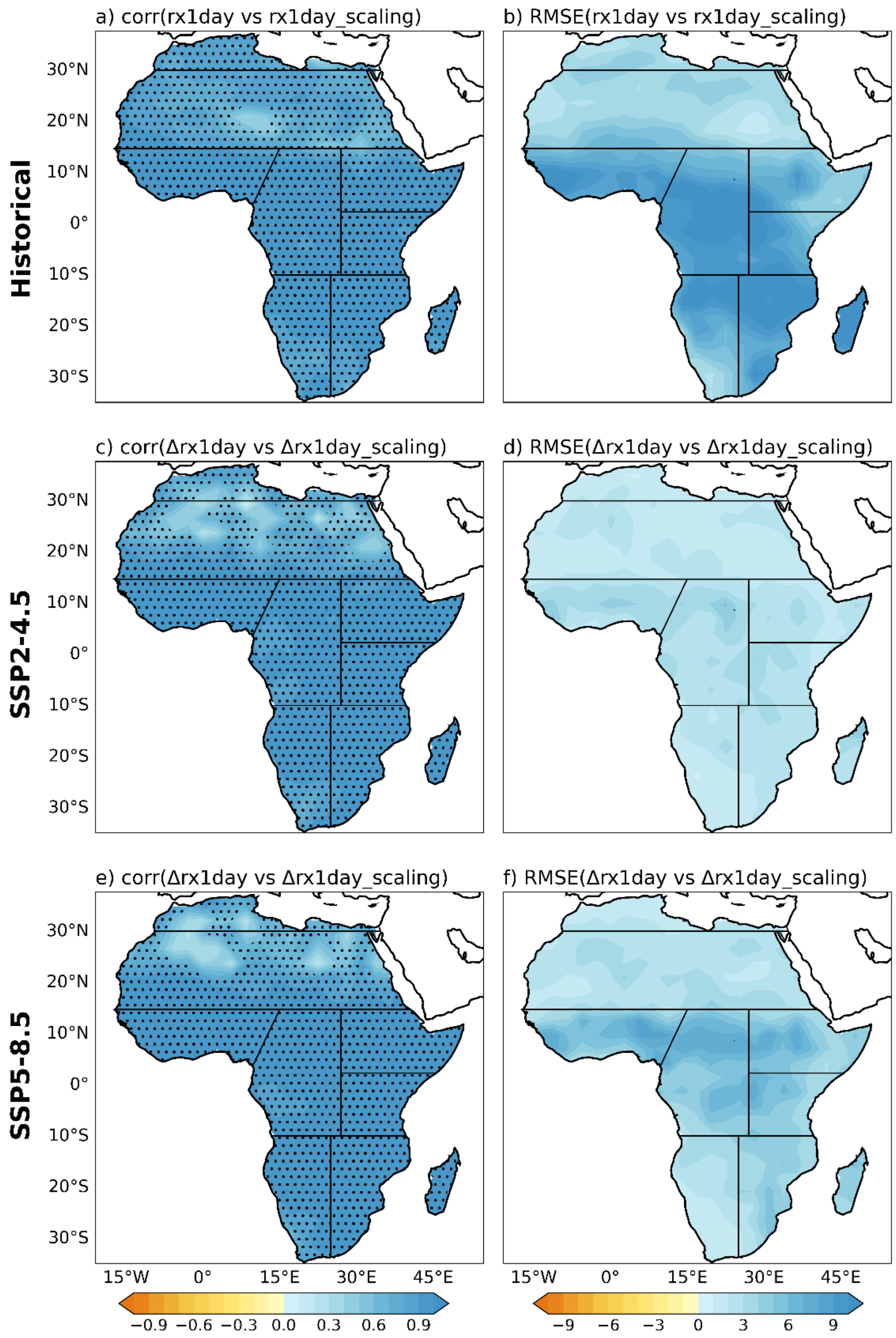

Figure S3. (a–b) Intermodel correlation and root-mean-square error (RMSE) between Rx1day and Rx1day_scaling for the historical period (1985–2014). Intermodel correlation and RMSE of projected changes in Rx1day and Rx1day_scaling under SSP2-4.5 (c–d) and under SSP5-8.5 (e–f). Stippling in (a, c, and e) indicates grid points where correlations are statistically significant at the 95% confidence level based on a Student's t-test. Changes are calculated as the difference between the 2070–2099 and 1985–2014 means.

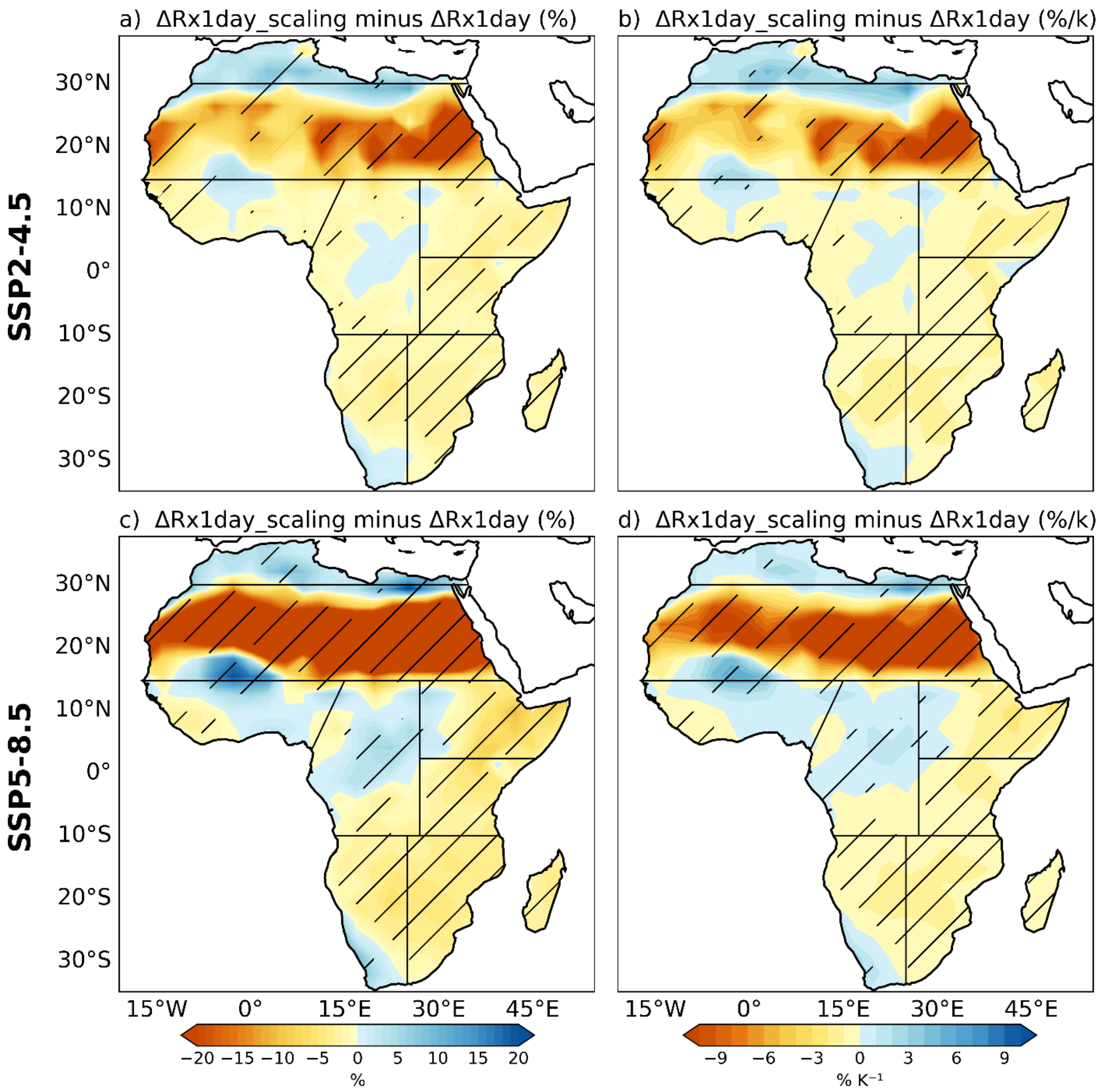

Figure S4. (a–d) EnsMean differences between projected changes in Rx1day and Rx1day_scaling under SSP2-4.5 and SSP5-8.5. Differences are shown as (a, c) percentages (%) and (b, d) % K^{-1} for the (a, b) SSP2-4.5 and (c, d) SSP5-8.5 scenarios. Hatched regions indicate grid points where differences are statistically significant at the 95% confidence level based on a Student's t-test. (a, c) Changes are calculated as the relative difference between the 2070–2099 and 1985–2014 means, and (b, d) are the same as (a, c) but scaled by the GMST change.

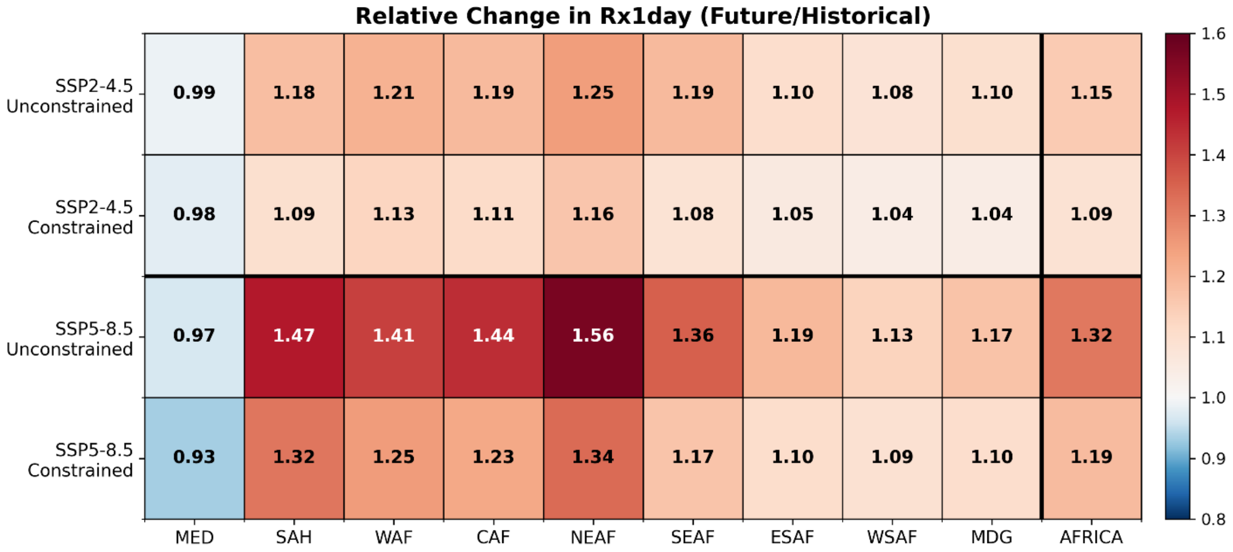

Figure S5. The relative change in Rx1day, expressed as the ratio of the weighted area-averaged future Rx1day (2070-2099) to the historical period (1985-2014), for the SSP2-4.5 and SSP5-8.5 scenarios across Africa and its subregions.

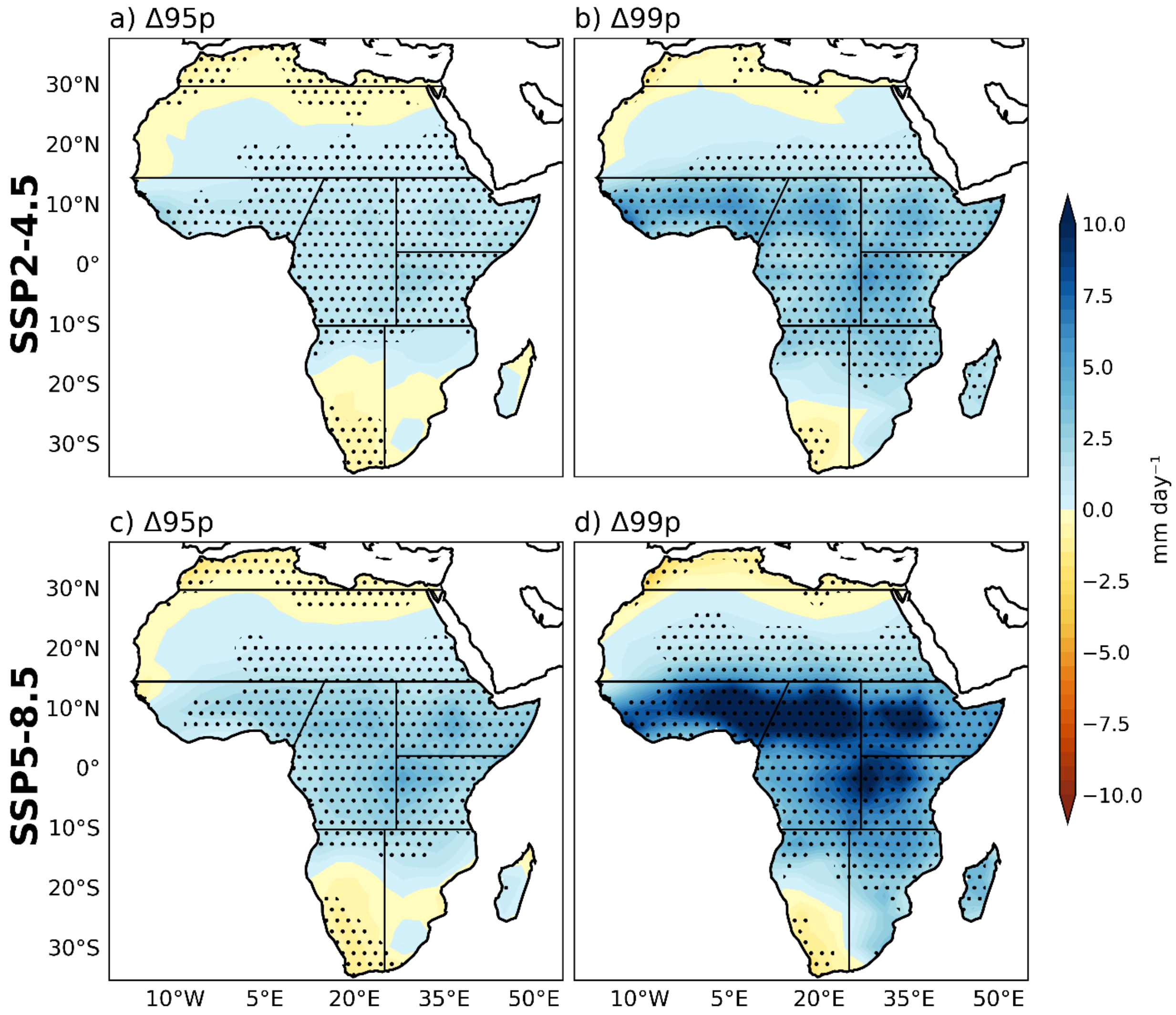

Figure S6. EnsMean changes (mm day^{-1}) in the 95th and 99th percentile precipitation under the SSP2-4.5 (a, b) and SSP5-8.5 (c, d) scenarios. Stippling indicates regions where at least 70% of models agree on the sign of the EnsMean change. Changes are calculated as the difference between the 2070–2099 and 1985–2014 means.

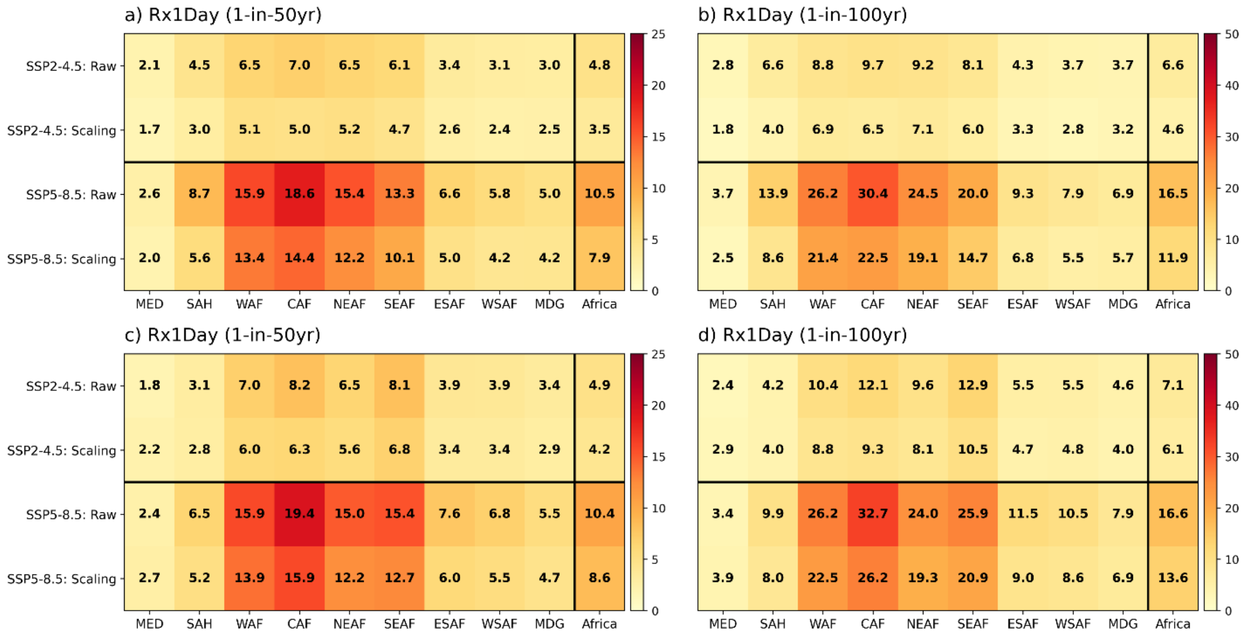

Figure S7. Area-weighted probability ratios for the occurrence of (a, c) 1-in-50-year and (b, d) 1-in-100-year future-to-historical Rx1day events, estimated using a (a, b) Gumbel extreme value and (c, d) GEV distribution, under the SSP2-4.5 and SSP5-8.5 scenarios. Results are presented for both the raw Rx1day projections and Rx1day_scaling, covering all of Africa and its individual subregions under both SSP2-4.5 and SSP5-8.5.

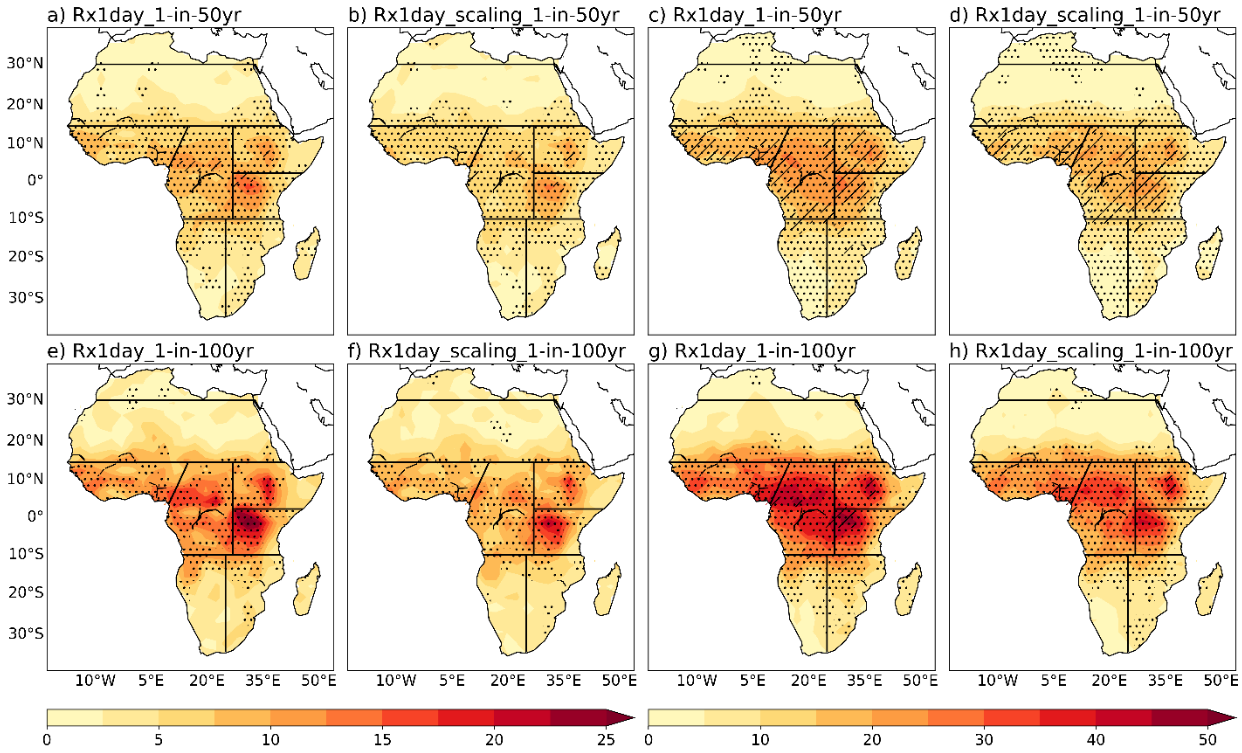

Figure S8. Probability ratios (PR) of (a,c,e,g) Rx1day and (b,d,f,h) Rx1day scaling frequency relative to the historical baseline period (1985–2014), estimated using the generalized extreme value distribution for (a-d) 1 in 50 year and (e-h) 1 in 100 year return periods under (a,b,e,f) SSP2 4.5 and (c,d,g,h) SSP5 8.5. Stippling and hatching denote grid points where at least 70% of models agree on the increase/decrease of EnsMean and where PR is statistically significant based on bootstrapping, respectively.

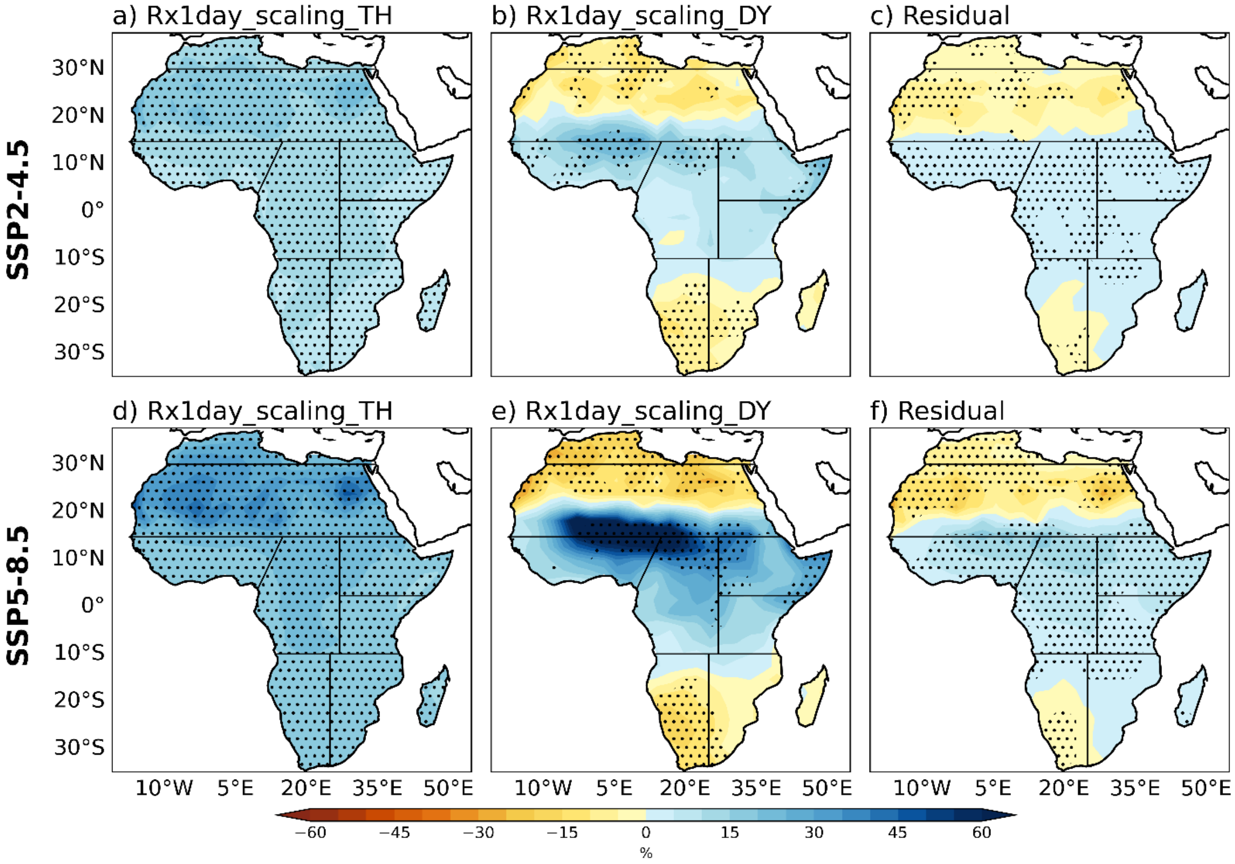

Figure S9. Ensemble-mean (EnsMean) changes (%) in thermodynamic, dynamic, and residual scaling under the (a-c) SSP2-4.5 and (d-f) SSP5-8.5 scenarios. Stippling denotes regions where at least 70% of models agree on the sign of EnsMean. Changes are calculated as the relative difference between the 2070–2099 and 1985–2014 means.

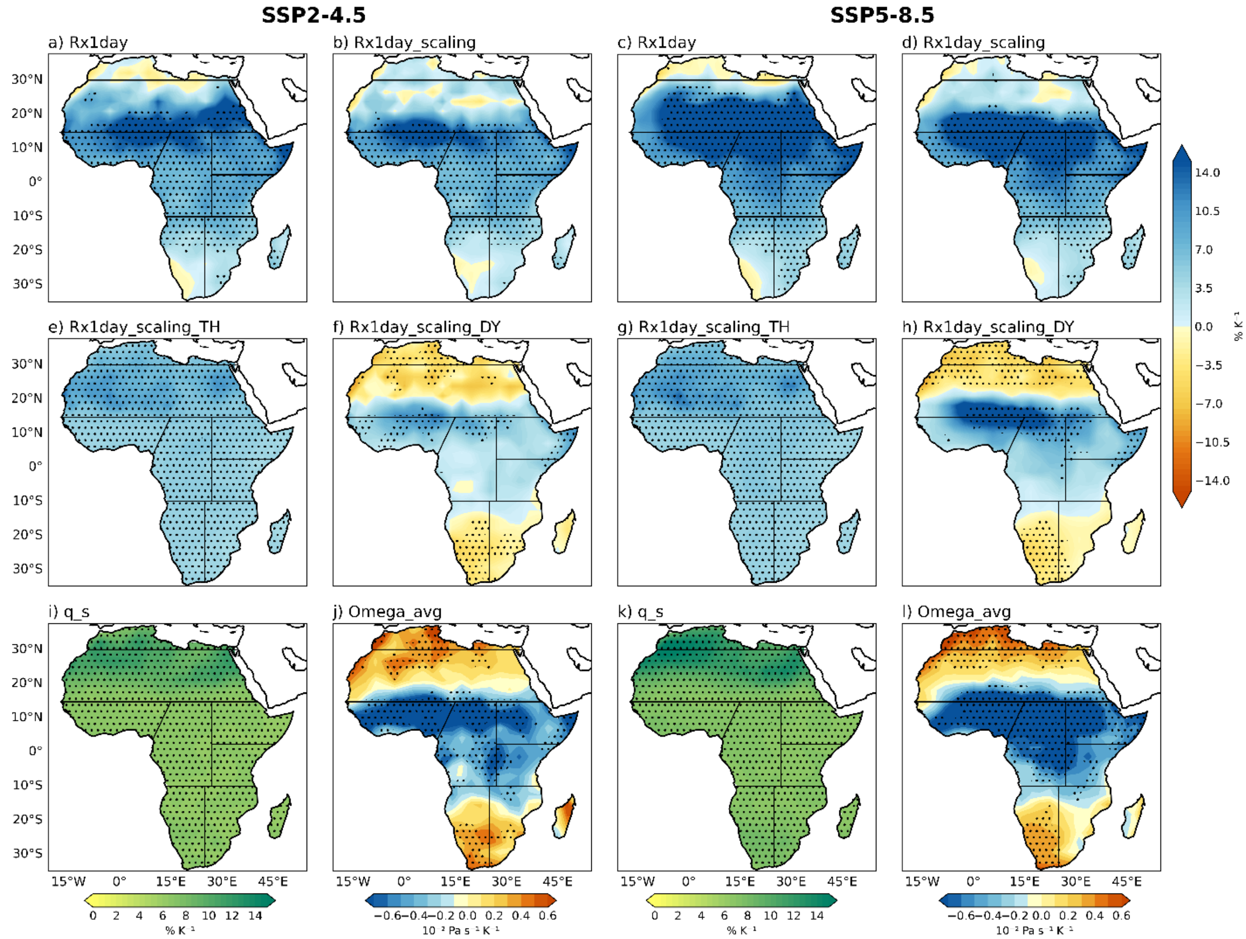

Figure S10. (a–d) EnsMean forced changes (% K^{-1}) in Rx1day and Rx1day_scaling under the SSP2-4.5 and SSP5-8.5 scenarios. (e–h) Thermodynamic and dynamic scaling under the SSP2-4.5 and SSP5-8.5 scenarios. For thermodynamic scaling, vertical velocity (ω) is held fixed at its 1985–2014 mean, whereas for dynamic scaling, the vertical derivative of saturation specific humidity is held fixed at its 1985–2014 mean. (i–l) Vertically integrated saturation specific humidity (q_s) and vertically averaged vertical velocity (negative values denote stronger ascent), both conditioned on extreme precipitation events under SSP2-4.5 and SSP5-8.5. Stippling indicates regions where at least 70% of models agree on the sign of EnsMean.

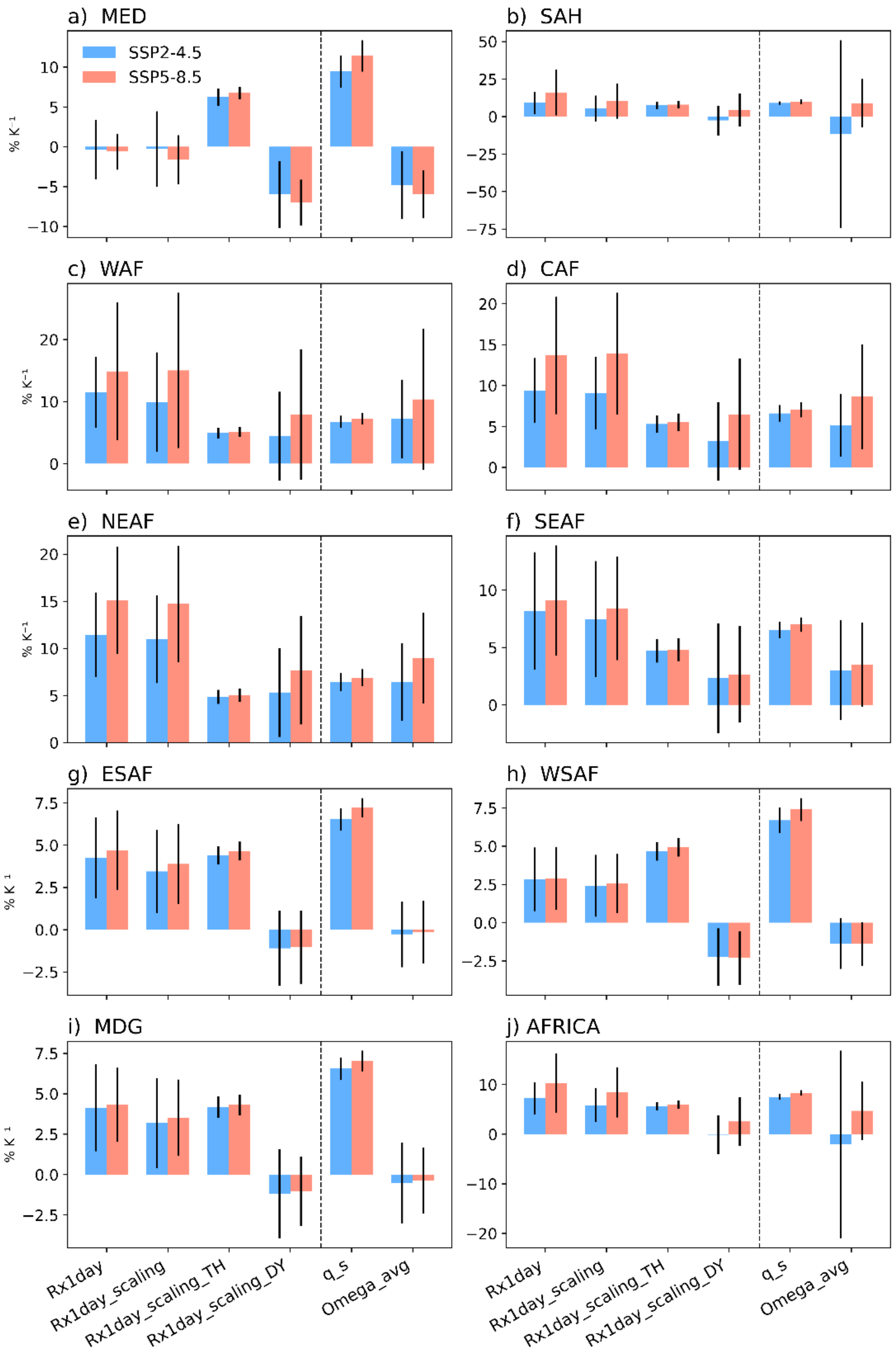

Figure S11. (a–j) Weighted area-averaged regional forced mean changes (%/K) in Rx1day, Rx1day_scaling, and individual scaling components (thermodynamic and dynamic), along with changes in vertically integrated saturation specific humidity and vertically averaged vertical velocity under the SSP2-4.5 and SSP5-8.5 scenarios. Bars represent the CMIP6 EnsMean, while vertical lines denote ±1 standard deviation, illustrating intermodel spread. Changes are calculated as the relative difference between the 2070–2099 and 1985–2014 means, scaled with GMST change.

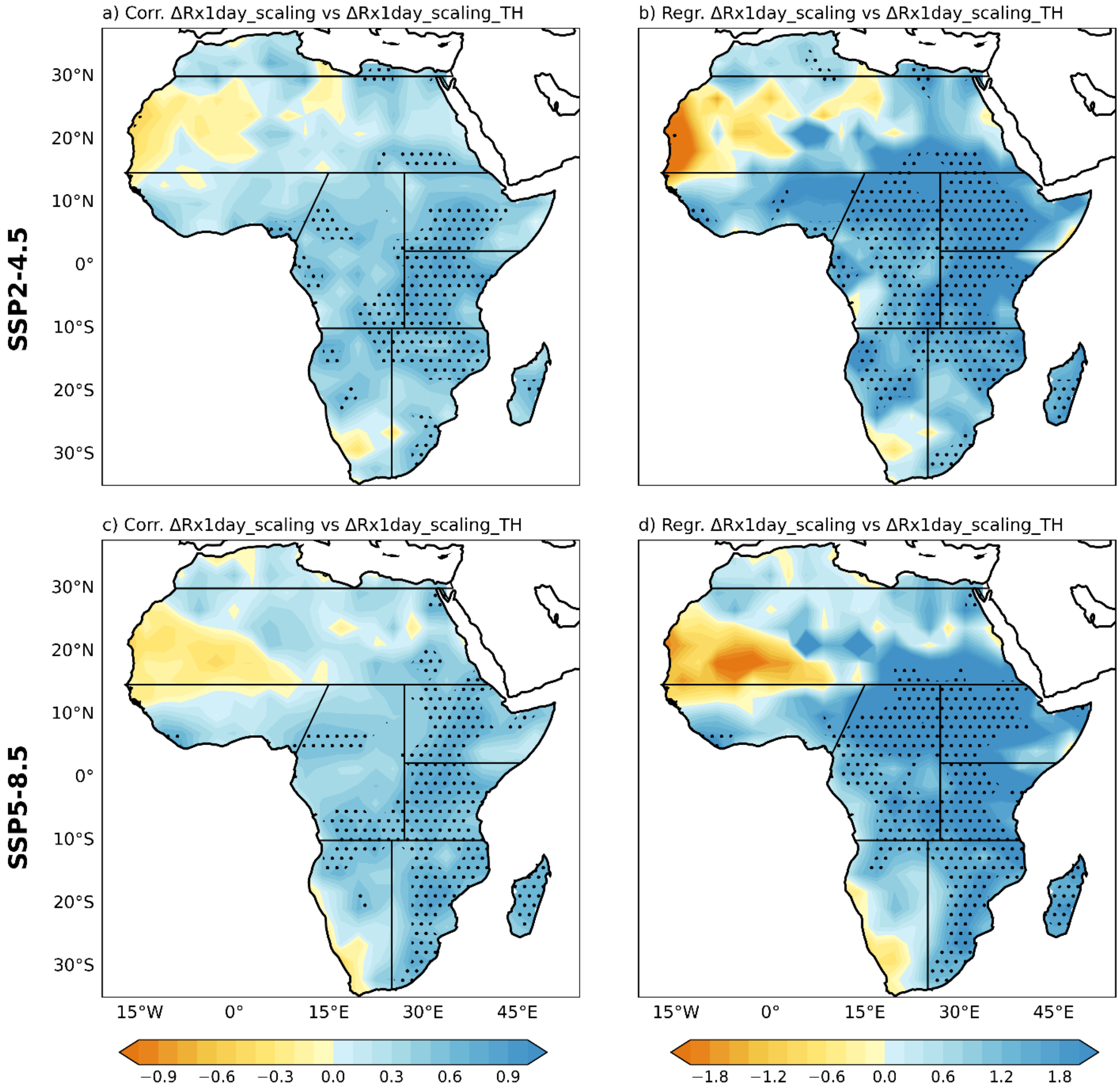

Figure S12. (a, c) Intermodel correlation and (b, d) regression between changes in Rx1day_scaling and its thermodynamic component under the (a, b) SSP2-4.5 and (c, d) SSP5-8.5 scenarios. Stippling indicates grid points where correlations/regressions are statistically significant at the 95% confidence level based on a Student's t-test. Changes are calculated as the difference between the 2070–2099 and 1985–2014 means.

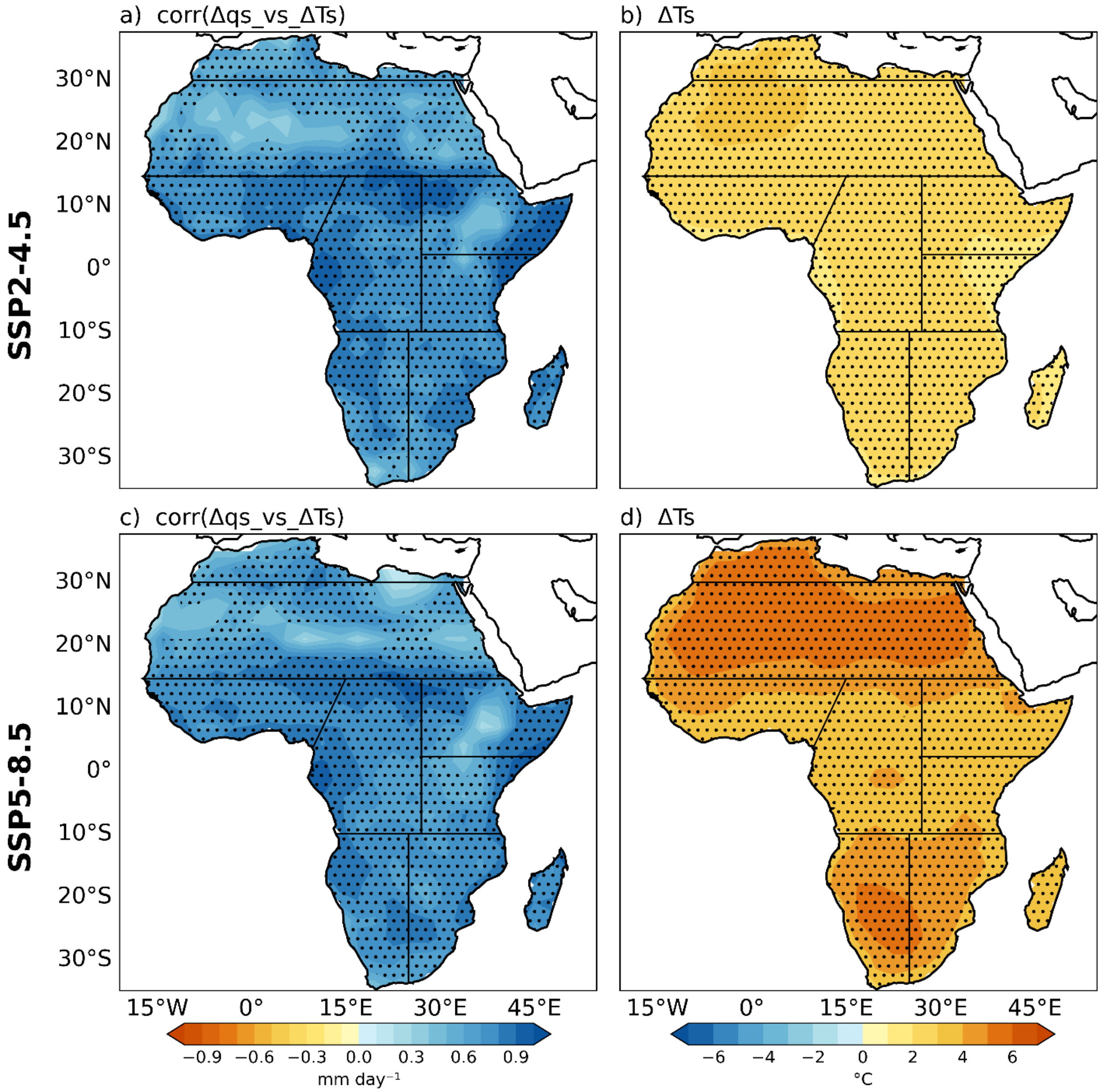

Figure S13. Intermodel correlation between changes in vertically integrated saturation specific humidity and changes in near-surface temperature under (a) SSP2-4.5 and (c) SSP5-8.5. Stippling indicates grid points where correlations are statistically significant at the 95% confidence level based on a Student's t-test. EnsMean changes in near-surface temperature (°C) are shown under (b) SSP2-4.5 and (d) SSP5-8.5. Stippling indicates regions where at least 70% of models agree on the sign of the EnsMean change. Changes are calculated as the difference between the 2070–2099 and 1985–2014 means.

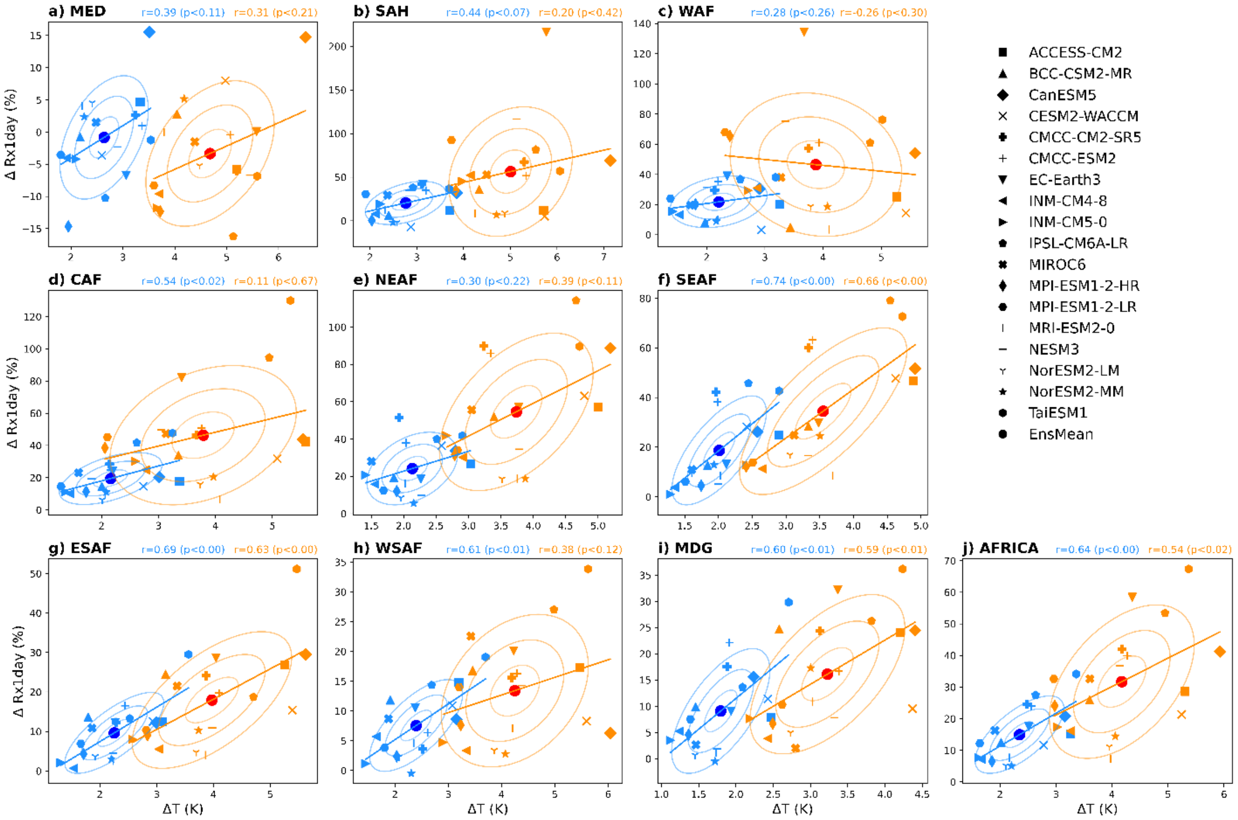

Figure S14. Intermodel relationship between projected changes in Rx1day and changes in near-surface temperature across Africa and its subregions under SSP2-4.5 and SSP5-8.5. Markers represent individual models. Ellipses denote the bivariate probability density of mean surface temperature changes and future Rx1day changes under the SSP2-4.5 (light blue) and SSP5-8.5 (orange) scenarios.

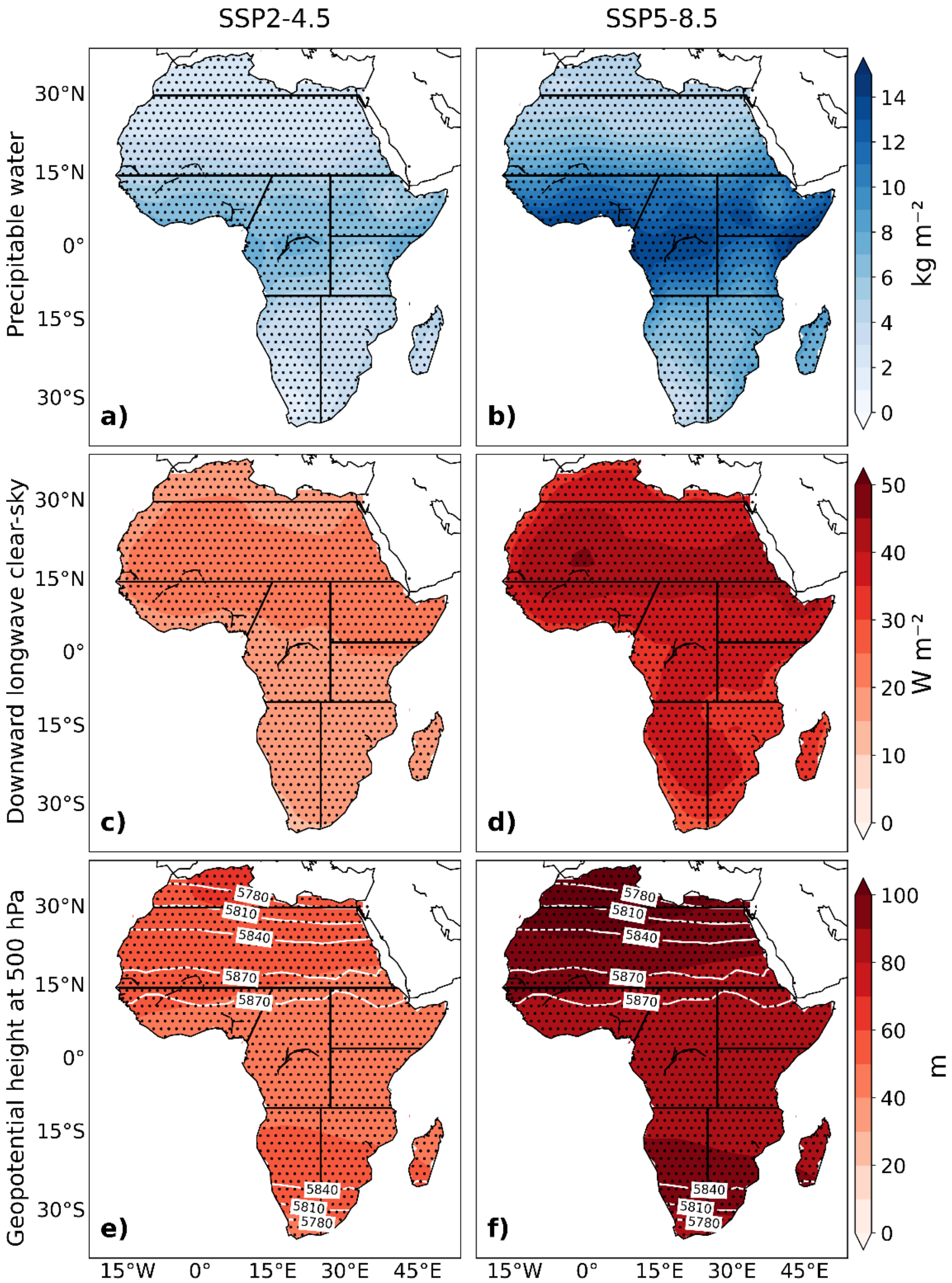

Figure S15. EnsMean changes in (a, b) precipitable water, (c, d) clear-sky downward longwave radiation, and (e, f) 500-hPa geopotential height for the late twenty-first century (2070–2099) relative to the historical baseline period (1985–2014) under the SSP2-4.5 and SSP5-8.5 scenarios. Stippling indicates regions where at least 70% of models agree on the sign of the EnsMean change. White contours in (e, f) indicate the climatological mean 500-hPa geopotential height during the historical baseline period (1985–2014).

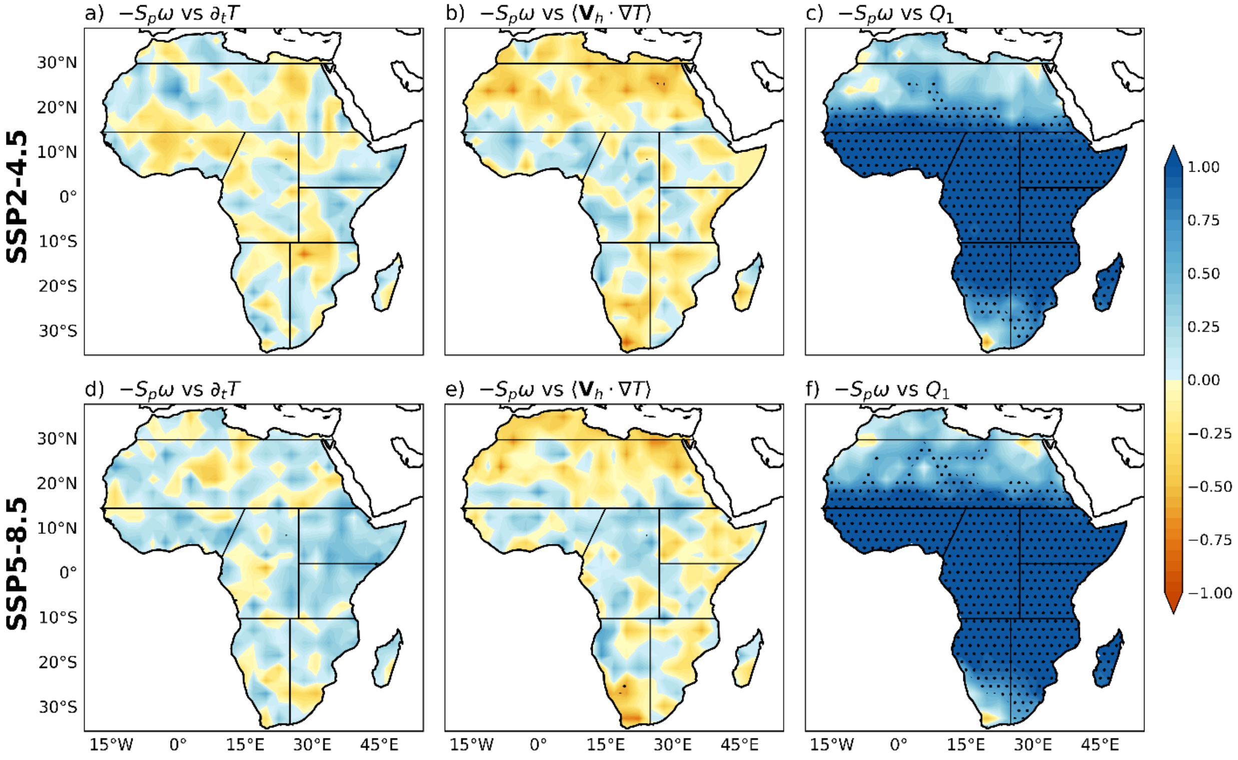

Figure S16. Intermodel spatial correlations between projected changes in vertical temperature advection ($-S_p\omega$) and (a, d) temperature tendency ($\partial_t T$), (b, e) horizontal temperature advection ($V_h \cdot \nabla T$), and (c, f) total diabatic heating (Q_1) under the SSP2-4.5 (a–c) and SSP5-8.5 (d–f) scenarios. Stippling indicates grid points where correlations are statistically significant at the 95% confidence level based on a two-sided Student's t-test. Changes are calculated as the difference between the 2070–2099 and 1985–2014 means.

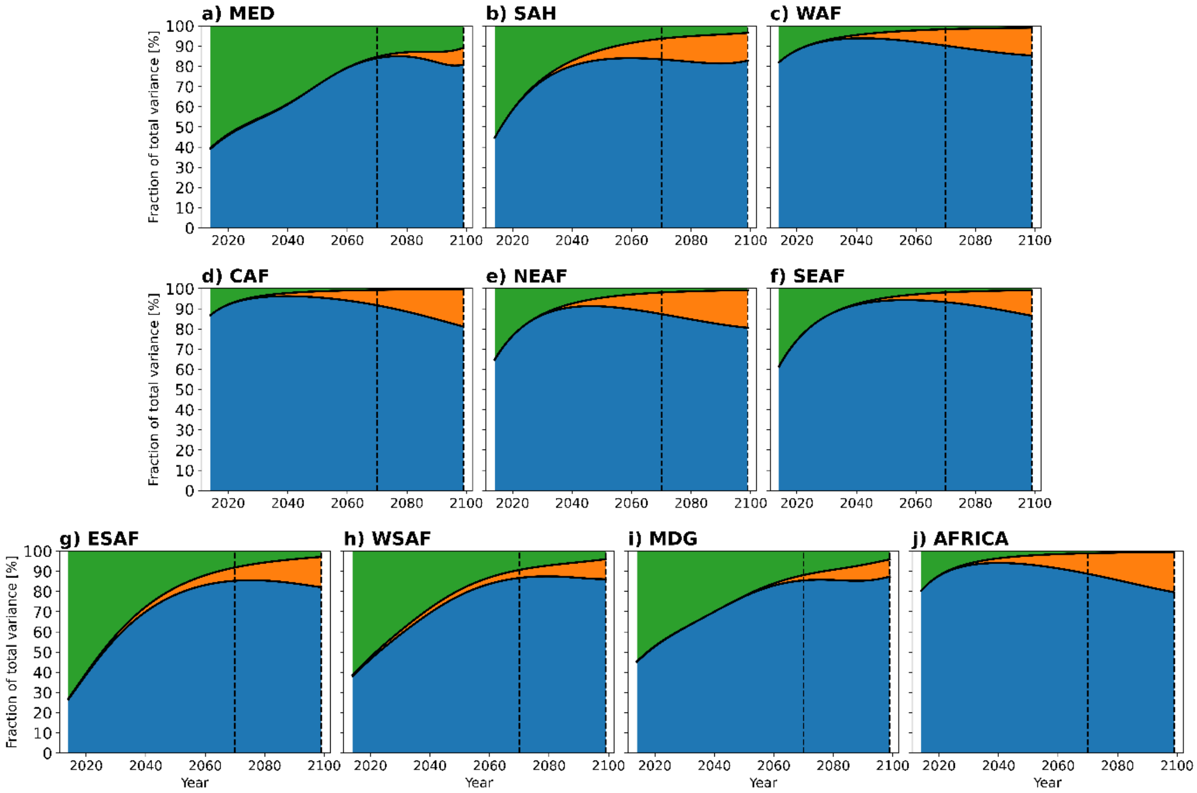

Figure S17. Percentage of total variance in projected annual maximum 1-day precipitation scaling (Rx1day_scaling) attributable to three sources of uncertainty, shown for the African regions (a–i) and for all of Africa (h). Blue, green, and orange shading denote model, internal variability, and scenario uncertainty, respectively.

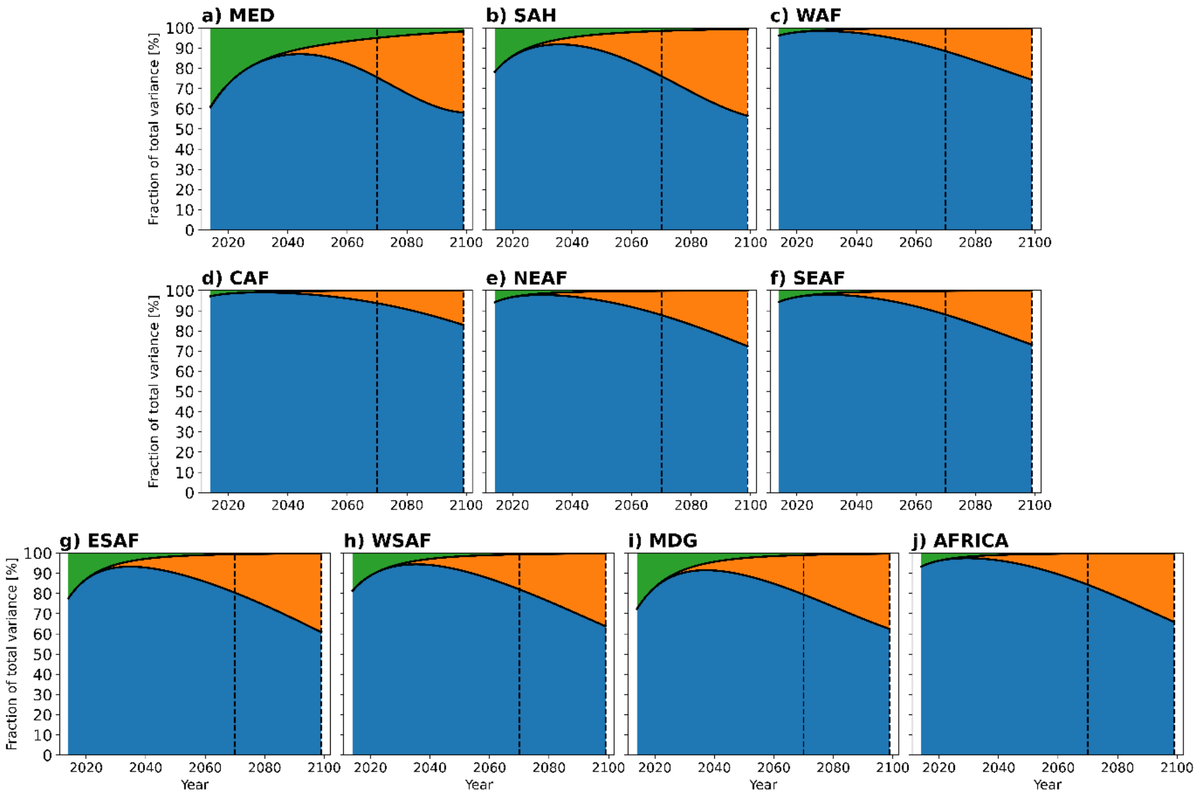

Figure S18. Percentage of total variance in the thermodynamic (TH) component of projected annual maximum 1-day precipitation scaling (Rx1day_scaling_TH) attributable to three sources of uncertainty, shown for the African regions (a–i) and for all of Africa (j). Blue, green, and orange shading denote model, internal variability, and scenario uncertainty, respectively.

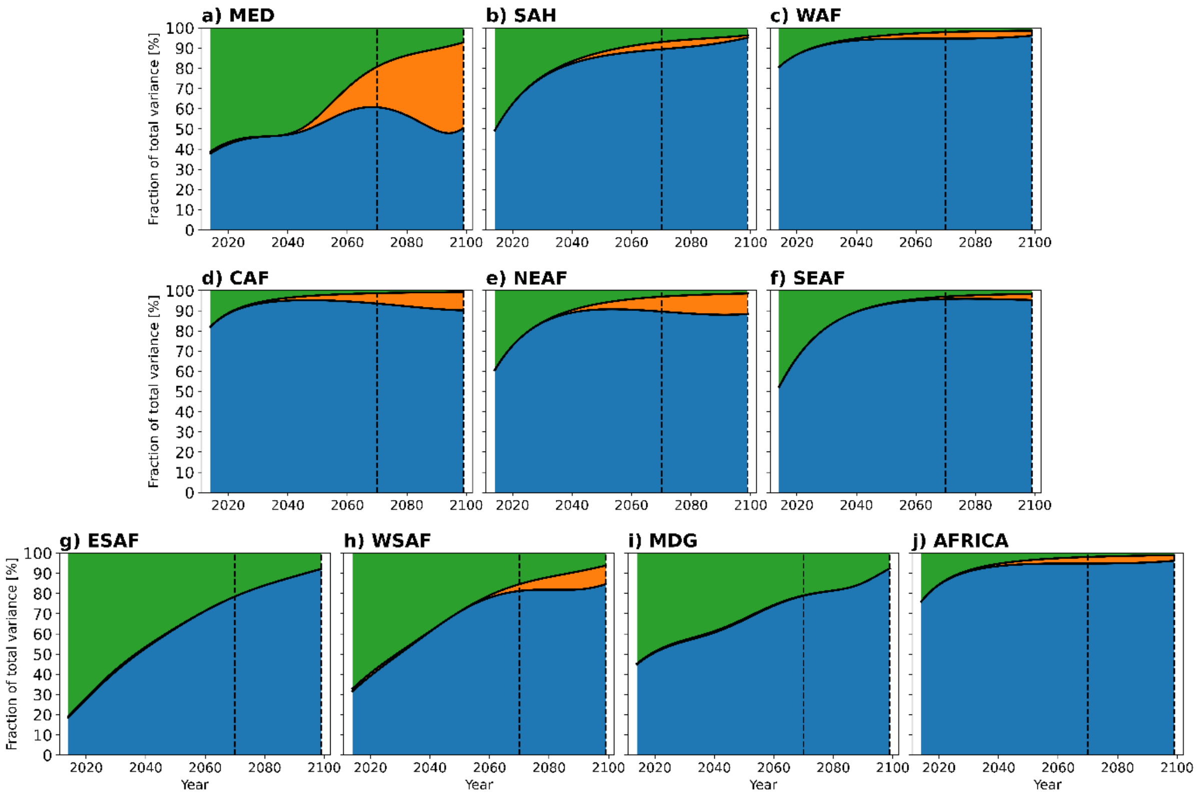

Figure S19. Percentage of total variance in the dynamic (DY) component of projected annual maximum 1-day precipitation scaling (Rx1day_scaling_DY) attributable to three sources of uncertainty, shown for the African regions (a–i) and for all of Africa (j). Blue, green, and orange shading denote model, internal variability, and scenario uncertainty, respectively.

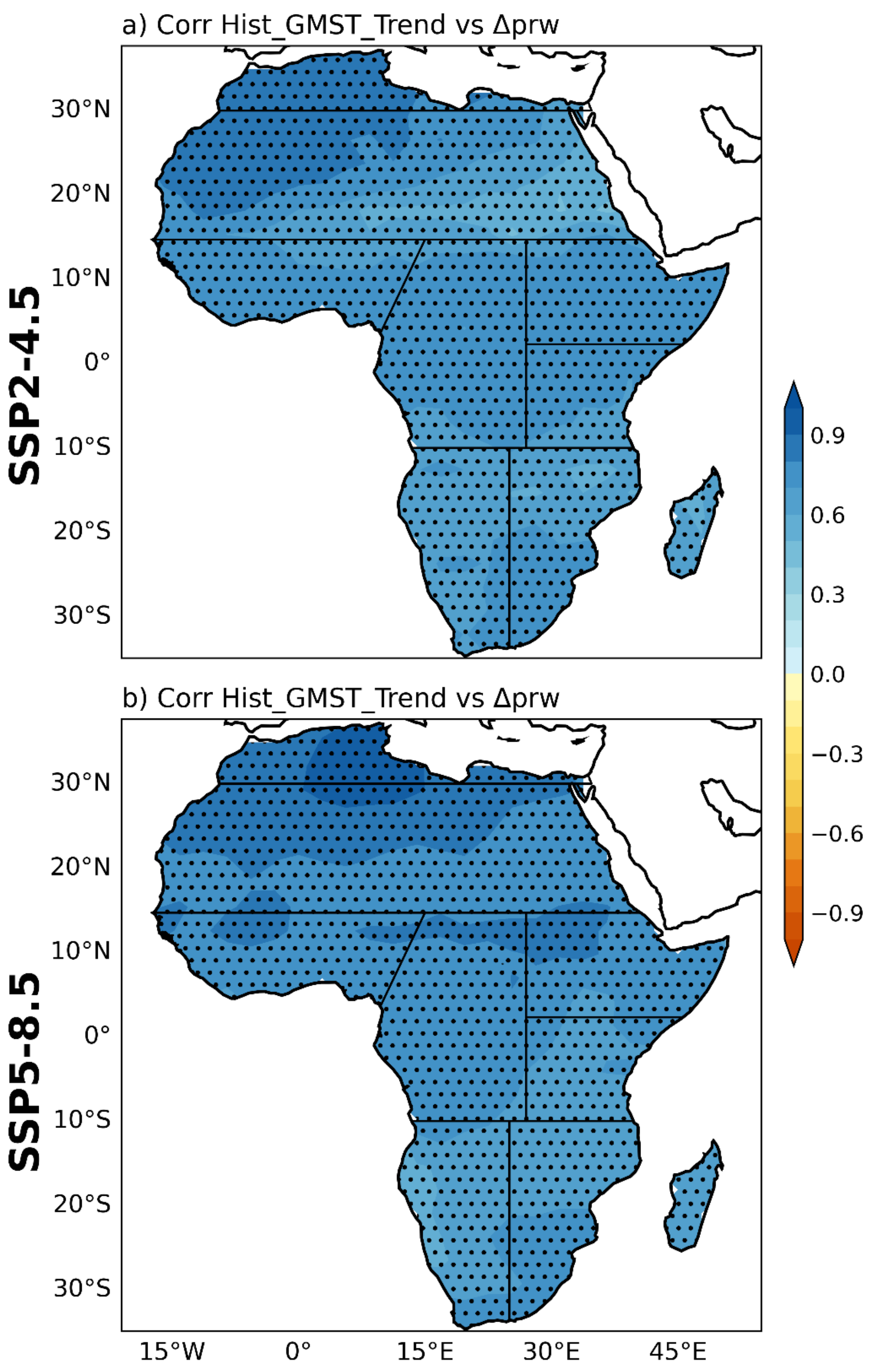

Figure S20. Intermodel correlation between historical (1985–2014) global mean surface temperature (GMST) trends and projected changes in column water vapor over Africa under the (a) SSP2-4.5 and (b) SSP5-8.5 scenarios. Stippling indicates grid points where correlations are statistically significant at the 95% confidence level based on a Student's t-test. Changes are calculated as the difference between the 2070–2099 and 1985–2014 means.

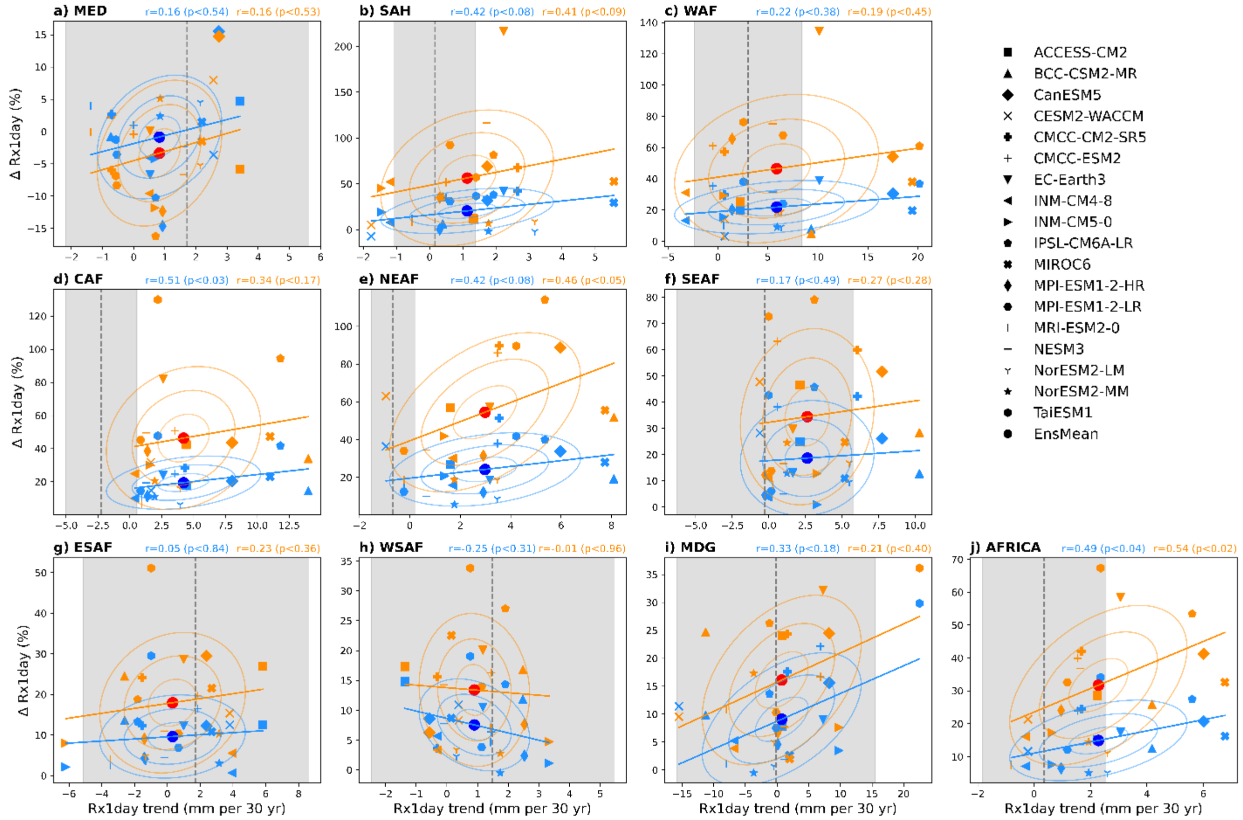

Figure S21. Intermodel relationship between projected changes in Rx1day (%) and historical (1985–2014) Rx1day trends across Africa and its subregions under SSP2-4.5 and SSP5-8.5. Markers represent individual models. Ellipses denote the bivariate probability density of Rx1day changes and historical Rx1day trends under the SSP2-4.5 (light blue) and SSP5-8.5 (orange) scenarios. The black vertical dashed line indicates the observed historical Rx1day trend, with uncertainty given by ±1 standard deviation (gray shading).

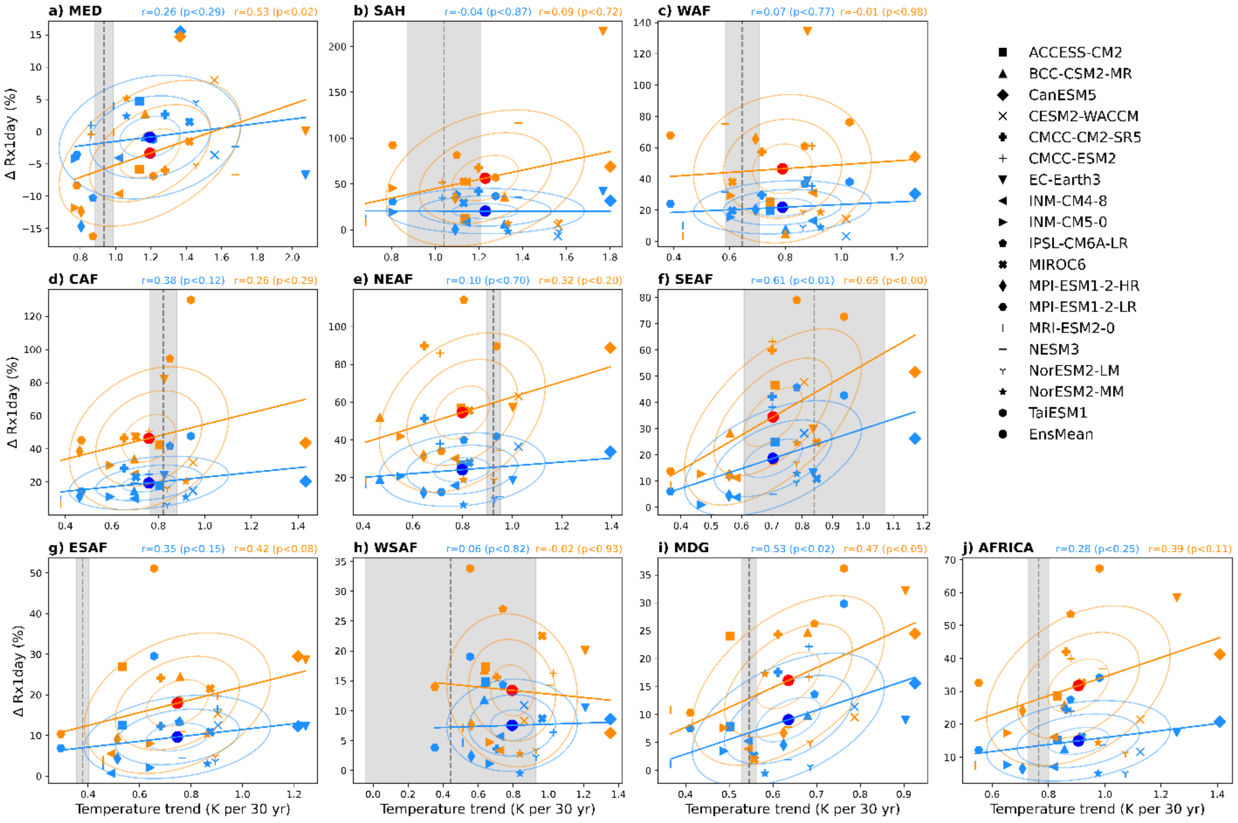

Figure S22. Intermodel relationship between projected changes in Rx1day (%) and historical (1985–2014) regional near-surface temperature trends across Africa and its subregions under SSP2-4.5 and SSP5-8.5. Markers represent individual models. Ellipses denote the bivariate probability density of Rx1day changes and historical regional near-surface temperature trends under the SSP2-4.5 (light blue) and SSP5-8.5 (orange) scenarios. The black vertical dashed line indicates the observed historical Rx1day trend, with uncertainty given by ±1 standard deviation (gray shading).

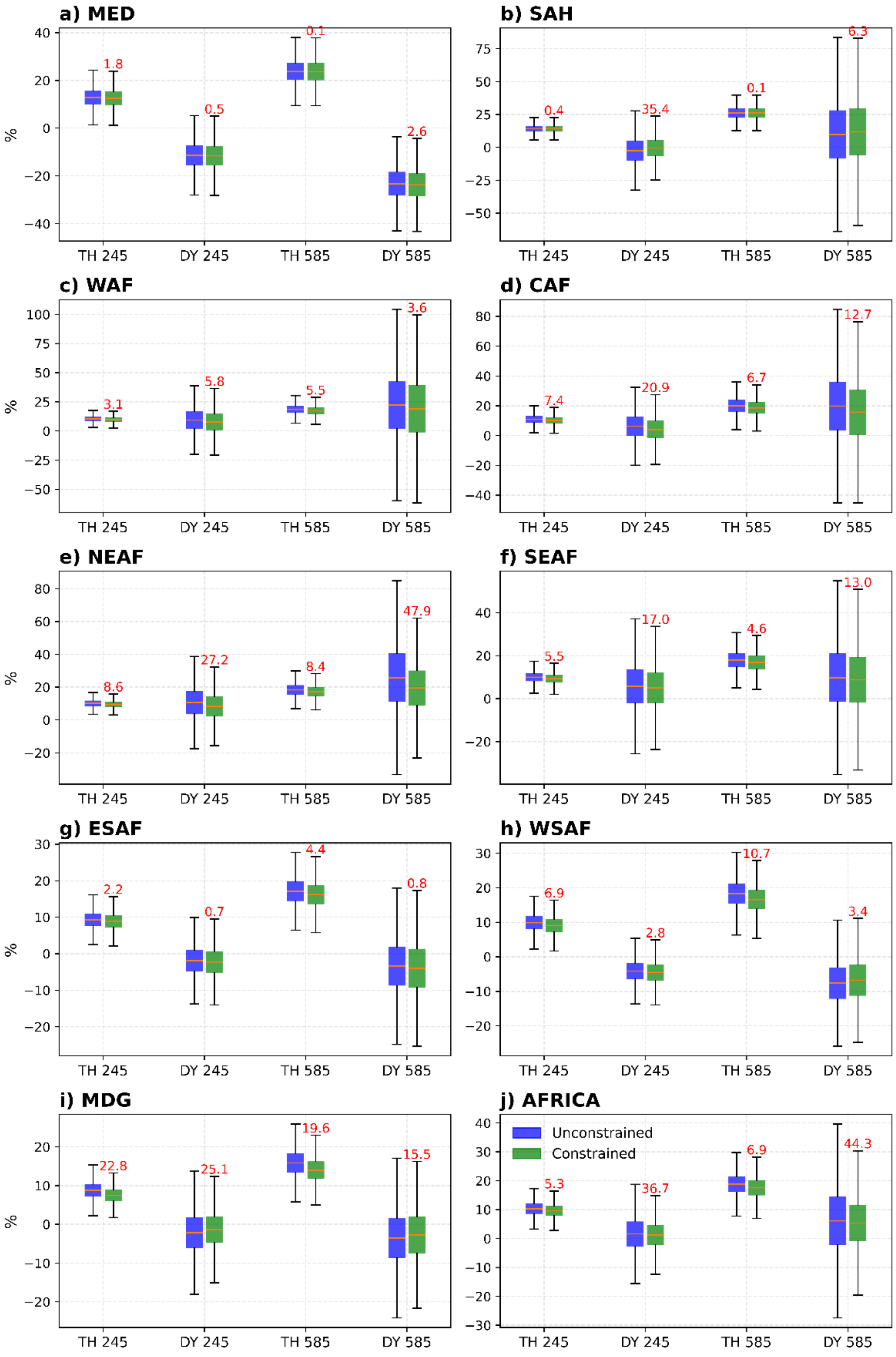

Figure S23. (a–i) Projected changes in the spread of unconstrained and constrained dynamic (DY) and thermodynamic (TH) components of Rx1day_scaling across Africa and its subregions under the SSP2-4.5 and SSP5-8.5 scenarios. Panels (a–j) show the distributions for each component and scenario. Box-and-whisker plots indicate the 10th, 25th, 50th (median), 75th, and 90th percentiles.

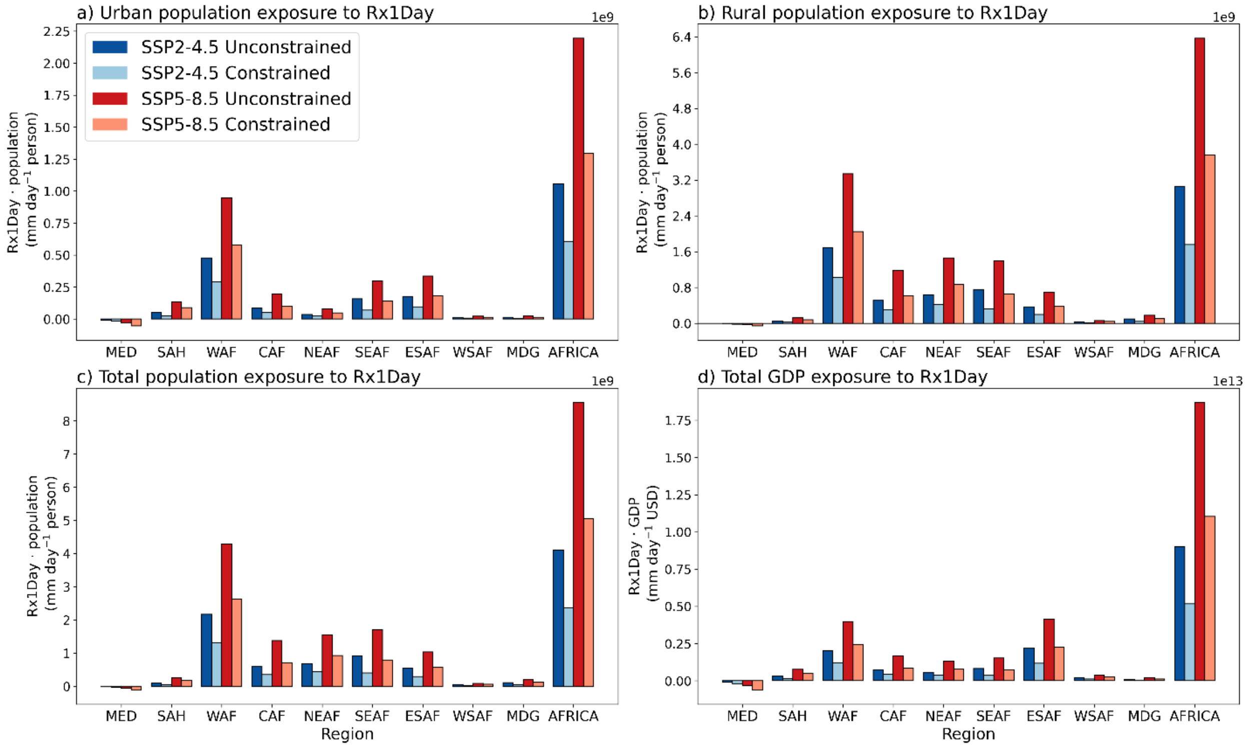

Figure S24. Bar plots show the contribution of climate effects (i.e., Rx1day) to total exposure for (a) urban population, (b) rural population, (c) total population, and (d) gross domestic product (GDP) across Africa and its subregions under the SSP2-4.5 and SSP5-8.5 scenarios. Changes are calculated for the future period (2070–2099) relative to the historical period (1985–2014).

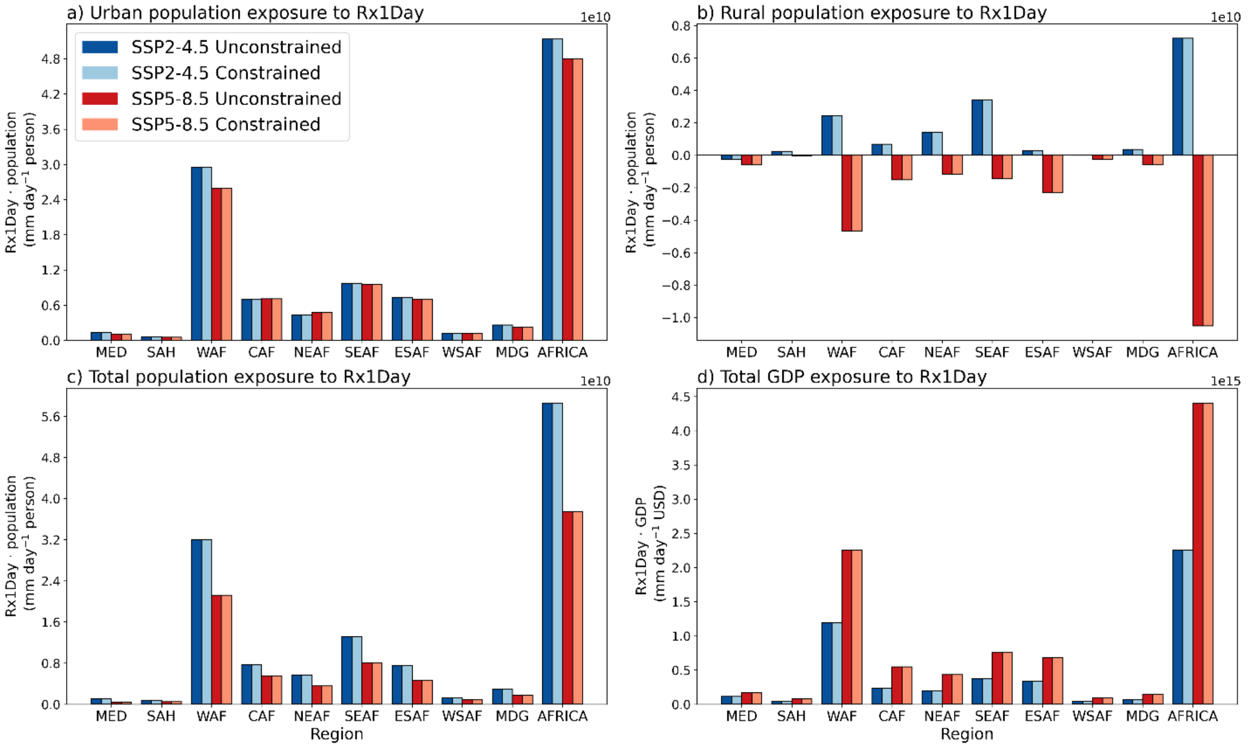

Figure S25. Bar plots show the contribution of population/GDP effects to total exposure for (a) urban population, (b) rural population, (c) total population, and (d) gross domestic product (GDP) across Africa and its subregions under the SSP2-4.5 and SSP5-8.5 scenarios. Changes are calculated for the future period (2070–2099) relative to the historical period (1985–2014).

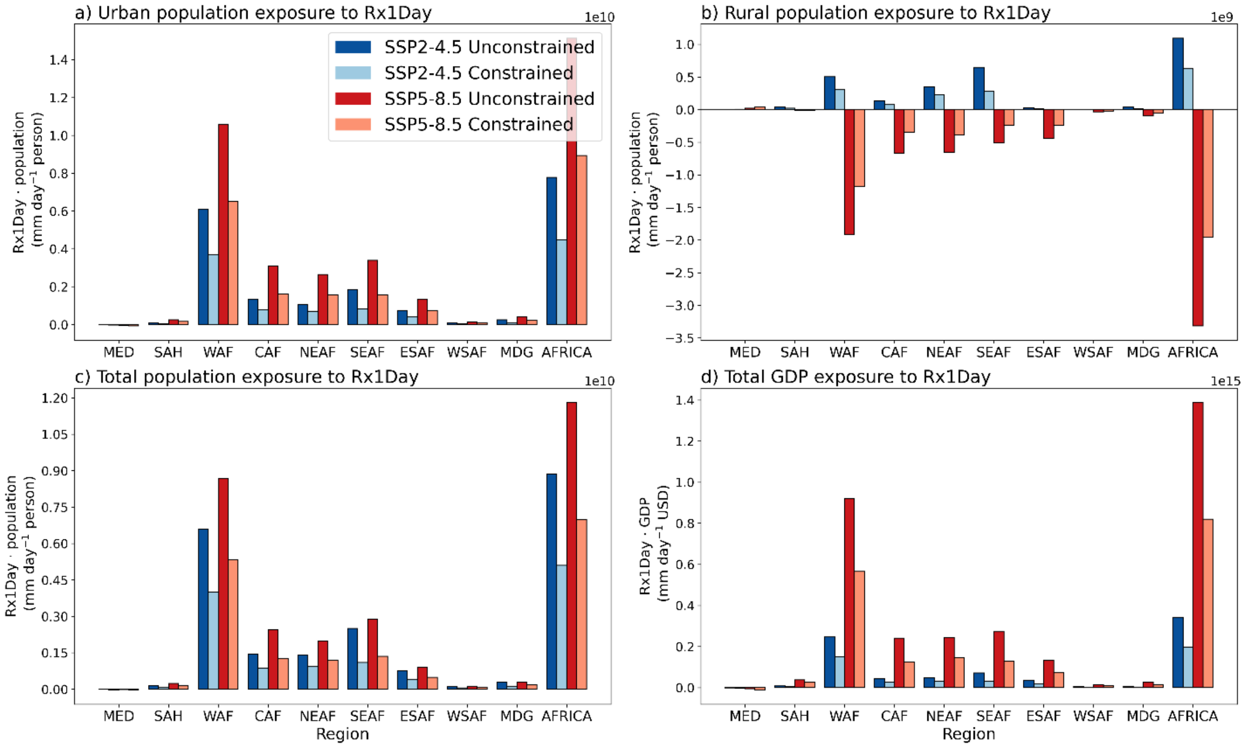

Figure S26. Bar plots show the contribution of interaction effects to total exposure for (a) urban population, (b) rural population, (c) total population, and (d) gross domestic product (GDP) across Africa and its subregions under the SSP2-4.5 and SSP5-8.5 scenarios. Changes are calculated for the future period (2070–2099) relative to the historical period (1985–2014).

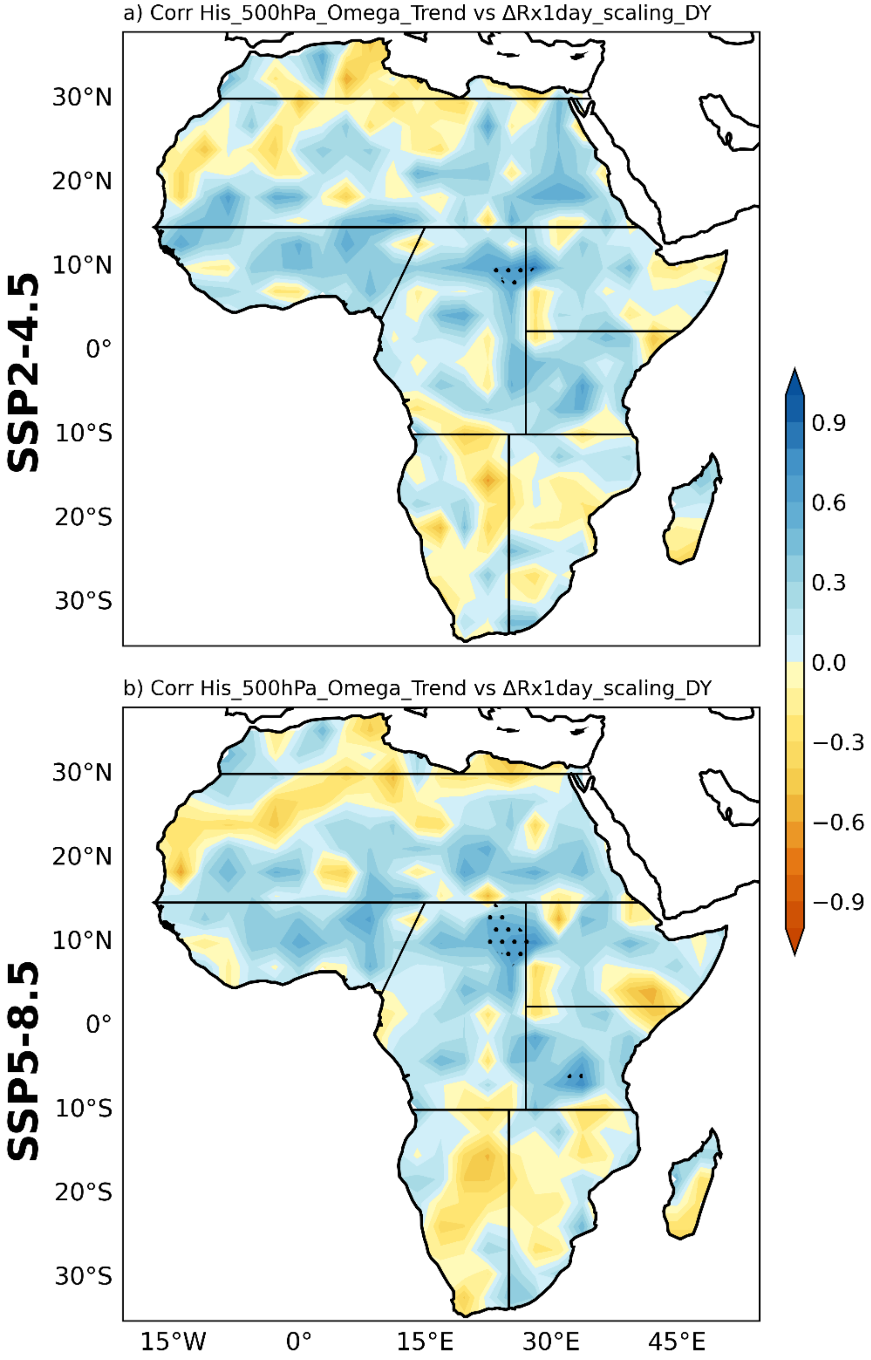

Figure S27. Intermodel correlation between historical (1985–2014) 500 hPa vertical velocity (omega) trends and projected changes in the dynamic (DY) component of Rx1day_scaling over Africa under the (a) SSP2-4.5 and (b) SSP5-8.5 scenarios. Stippling indicates grid points where correlations are statistically significant at the 95% confidence level based on a Student's t-test. Changes are calculated as the difference between the 2070–2099 and 1985–2014 means.

Supplementary References:

1. Dix, M. *et al.* CSIRO-ARCCSS ACCESS-CM2 model output prepared for CMIP6 CMIP historical. Earth System Grid Federation https://doi.org/10.22033/ESGF/CMIP6.4271 (2019).
2. Dix, M. *et al.* CSIRO-ARCCSS ACCESS-CM2 model output prepared for CMIP6 ScenarioMIP ssp245. Earth System Grid Federation https://doi.org/10.22033/ESGF/CMIP6.4321 (2019).
3. Dix, M. *et al.* CSIRO-ARCCSS ACCESS-CM2 model output prepared for CMIP6 ScenarioMIP ssp585. Earth System Grid Federation https://doi.org/10.22033/ESGF/CMIP6.4332 (2019).
4. Wu, T. *et al.* BCC BCC-CSM2MR model output prepared for CMIP6 CMIP historical. Earth System Grid Federation https://doi.org/10.22033/ESGF/CMIP6.2948 (2018).
5. Xin, X. *et al.* BCC BCC-CSM2MR model output prepared for CMIP6 ScenarioMIP ssp245. Earth System Grid Federation https://doi.org/10.22033/ESGF/CMIP6.3030 (2019).
6. Xin, X. *et al.* BCC BCC-CSM2MR model output prepared for CMIP6 ScenarioMIP ssp585. Earth System Grid Federation https://doi.org/10.22033/ESGF/CMIP6.3050 (2019).
7. Swart, N. C. *et al.* CCCma CanESM5 model output prepared for CMIP6 CMIP historical. Earth System Grid Federation https://doi.org/10.22033/ESGF/CMIP6.3610 (2019).
8. Swart, N. C. *et al.* CCCma CanESM5 model output prepared for CMIP6 ScenarioMIP ssp245. Earth System Grid Federation https://doi.org/10.22033/ESGF/CMIP6.3685 (2019).
9. Swart, N. C. *et al.* CCCma CanESM5 model output prepared for CMIP6 ScenarioMIP ssp585. Earth System Grid Federation https://doi.org/10.22033/ESGF/CMIP6.3696 (2019).
10. Danabasoglu, G. NCAR CESM2-WACCM model output prepared for CMIP6 CMIP historical. Earth System Grid Federation https://doi.org/10.22033/ESGF/CMIP6.10071 (2019).
11. Danabasoglu, G. NCAR CESM2-WACCM model output prepared for CMIP6 ScenarioMIP ssp245. Earth System Grid Federation https://doi.org/10.22033/ESGF/CMIP6.10101 (2019).
12. Danabasoglu, G. NCAR CESM2-WACCM model output prepared for CMIP6 ScenarioMIP ssp585. Earth System Grid Federation https://doi.org/10.22033/ESGF/CMIP6.10115 (2019).
13. Lovato, T. & Peano, D. CMCC CMCC-CM2-SR5 model output prepared for CMIP6 CMIP historical. Earth System Grid Federation https://doi.org/10.22033/ESGF/CMIP6.3825 (2020).

14. Lovato, T. & Peano, D. CMCC CMCC-CM2-SR5 model output prepared for CMIP6 ScenarioMIP ssp245. Earth System Grid Federation https://doi.org/10.22033/ESGF/CMIP6.3889 (2020).

15. Lovato, T. & Peano, D. CMCC CMCC-CM2-SR5 model output prepared for CMIP6 ScenarioMIP ssp585. Earth System Grid Federation https://doi.org/10.22033/ESGF/CMIP6.3896 (2020).

16. Lovato, T., Peano, D. & Butenschön, M. CMCC CMCC-ESM2 model output prepared for CMIP6 CMIP historical. Earth System Grid Federation https://doi.org/10.22033/ESGF/CMIP6.13195 (2021).

17. Lovato, T., Peano, D. & Butenschön, M. CMCC CMCC-ESM2 model output prepared for CMIP6 ScenarioMIP ssp245. Earth System Grid Federation https://doi.org/10.22033/ESGF/CMIP6.13252 (2021).

18. Lovato, T., Peano, D. & Butenschön, M. CMCC CMCC-ESM2 model output prepared for CMIP6 ScenarioMIP ssp585. Earth System Grid Federation https://doi.org/10.22033/ESGF/CMIP6.13259 (2021).

19. EC-Earth Consortium (EC-Earth). EC-Earth-Consortium EC-Earth3 model output prepared for CMIP6 CMIP historical. Earth System Grid Federation https://doi.org/10.22033/ESGF/CMIP6.4700 (2019).

20. EC-Earth Consortium (EC-Earth). EC-Earth-Consortium EC-Earth3 model output prepared for CMIP6 ScenarioMIP ssp245. Earth System Grid Federation https://doi.org/10.22033/ESGF/CMIP6.4880 (2019).

21. EC-Earth Consortium (EC-Earth). EC-Earth-Consortium EC-Earth3 model output prepared for CMIP6 ScenarioMIP ssp585. Earth System Grid Federation https://doi.org/10.22033/ESGF/CMIP6.4912 (2019).

22. Volodin, E. *et al.* INM INM-CM4-8 model output prepared for CMIP6 CMIP historical. Earth System Grid Federation https://doi.org/10.22033/ESGF/CMIP6.5069 (2019).

23. Volodin, E. *et al.* INM INM-CM4-8 model output prepared for CMIP6 ScenarioMIP ssp245. Earth System Grid Federation https://doi.org/10.22033/ESGF/CMIP6.12327 (2019).

24. Volodin, E. *et al.* INM INM-CM4-8 model output prepared for CMIP6 ScenarioMIP ssp585. Earth System Grid Federation https://doi.org/10.22033/ESGF/CMIP6.12337 (2019).

25. Volodin, E. *et al.* INM INM-CM5-0 model output prepared for CMIP6 CMIP historical. Earth System Grid Federation https://doi.org/10.22033/ESGF/CMIP6.5070 (2019).

26. Volodin, E. *et al.* INM INM-CM5-0 model output prepared for CMIP6 ScenarioMIP ssp245. Earth System Grid Federation https://doi.org/10.22033/ESGF/CMIP6.12328 (2019).

27. Volodin, E. *et al.* INM INM-CM5-0 model output prepared for CMIP6 ScenarioMIP ssp585. Earth System Grid Federation https://doi.org/10.22033/ESGF/CMIP6.12338 (2019).

28. Boucher, O. *et al.* IPSL IPSL-CM6A-LR model output prepared for CMIP6 CMIP historical. Earth System Grid Federation https://doi.org/10.22033/ESGF/CMIP6.5195 (2018).

29. Boucher, O. *et al.* IPSL IPSL-CM6A-LR model output prepared for CMIP6 ScenarioMIP ssp245. Earth System Grid Federation https://doi.org/10.22033/ESGF/CMIP6.5264 (2019).

30. Boucher, O. *et al.* IPSL IPSL-CM6A-LR model output prepared for CMIP6 ScenarioMIP ssp585. Earth System Grid Federation https://doi.org/10.22033/ESGF/CMIP6.5271 (2019).

31. Tatebe, H. & Watanabe, M. MIROC MIROC6 model output prepared for CMIP6 CMIP historical. Earth System Grid Federation https://doi.org/10.22033/ESGF/CMIP6.5603 (2018).

32. Shiogama, H., Abe, M. & Tatebe, H. MIROC MIROC6 model output prepared for CMIP6 ScenarioMIP ssp245. Earth System Grid Federation https://doi.org/10.22033/ESGF/CMIP6.5746 (2019).

33. Shiogama, H., Abe, M. & Tatebe, H. MIROC MIROC6 model output prepared for CMIP6 ScenarioMIP ssp585. Earth System Grid Federation https://doi.org/10.22033/ESGF/CMIP6.5771 (2019).

34. Jungclaus, J. *et al.* MPI-M MPI-ESM1.2-HR model output prepared for CMIP6 CMIP historical. Earth System Grid Federation https://doi.org/10.22033/ESGF/CMIP6.6594 (2019).

35. Schupfner, M. *et al.* DKRZ MPI-ESM1.2-HR model output prepared for CMIP6 ScenarioMIP ssp245. Earth System Grid Federation https://doi.org/10.22033/ESGF/CMIP6.4398 (2019).

36. Schupfner, M. *et al.* DKRZ MPI-ESM1.2-HR model output prepared for CMIP6 ScenarioMIP ssp585. Earth System Grid Federation https://doi.org/10.22033/ESGF/CMIP6.4403 (2019).

37. Wieners, K.-H. *et al.* MPI-M MPI-ESM1.2-LR model output prepared for CMIP6 CMIP historical. Earth System Grid Federation https://doi.org/10.22033/ESGF/CMIP6.6595 (2019).

38. Wieners, K.-H. *et al.* MPI-M MPI-ESM1.2-LR model output prepared for CMIP6 ScenarioMIP ssp245. Earth System Grid Federation https://doi.org/10.22033/ESGF/CMIP6.6693 (2019).

39. Wieners, K.-H. *et al.* MPI-M MPI-ESM1.2-LR model output prepared for CMIP6 ScenarioMIP ssp585. Earth System Grid Federation https://doi.org/10.22033/ESGF/CMIP6.6705 (2019).

40. Yukimoto, S. *et al.* MRI MRI-ESM2.0 model output prepared for CMIP6 CMIP historical. Earth System Grid Federation https://doi.org/10.22033/ESGF/CMIP6.6842 (2019).

41. Yukimoto, S. *et al.* MRI MRI-ESM2.0 model output prepared for CMIP6 ScenarioMIP ssp245. Earth System Grid Federation https://doi.org/10.22033/ESGF/CMIP6.6910 (2019).

42. Yukimoto, S. *et al.* MRI MRI-ESM2.0 model output prepared for CMIP6 ScenarioMIP ssp585. Earth System Grid Federation https://doi.org/10.22033/ESGF/CMIP6.6929 (2019).

43. Cao, J. & Wang, B. NUIST NESMv3 model output prepared for CMIP6 CMIP historical. Earth System Grid Federation https://doi.org/10.22033/ESGF/CMIP6.8769 (2019).

44. Cao, J. NUIST NESMv3 model output prepared for CMIP6 ScenarioMIP ssp245. Earth System Grid Federation https://doi.org/10.22033/ESGF/CMIP6.8781 (2019).

45. Cao, J. NUIST NESMv3 model output prepared for CMIP6 ScenarioMIP ssp585. Earth System Grid Federation https://doi.org/10.22033/ESGF/CMIP6.8790 (2019).

46. Seland, Ø. *et al.* NCC NorESM2-LM model output prepared for CMIP6 CMIP historical. Earth System Grid Federation https://doi.org/10.22033/ESGF/CMIP6.8036 (2019).

47. Seland, Ø. *et al.* NCC NorESM2-LM model output prepared for CMIP6 ScenarioMIP ssp245. Earth System Grid Federation https://doi.org/10.22033/ESGF/CMIP6.8253 (2019).

48. Seland, Ø. *et al.* NCC NorESM2-LM model output prepared for CMIP6 ScenarioMIP ssp585. Earth System Grid Federation https://doi.org/10.22033/ESGF/CMIP6.8319 (2019).

49. Bentsen, M. *et al.* NCC NorESM2-MM model output prepared for CMIP6 CMIP historical. Earth System Grid Federation https://doi.org/10.22033/ESGF/CMIP6.8040 (2019).

50. Bentsen, M. *et al.* NCC NorESM2-MM model output prepared for CMIP6 ScenarioMIP ssp245. Earth System Grid Federation https://doi.org/10.22033/ESGF/CMIP6.8255 (2019).

51. Bentsen, M. *et al.* NCC NorESM2-MM model output prepared for CMIP6 ScenarioMIP ssp585. Earth System Grid Federation https://doi.org/10.22033/ESGF/CMIP6.8321 (2019).

52. Lee, W.-L. & Liang, H.-C. AS-RCEC TaiESM1.0 model output prepared for CMIP6 CMIP historical. Earth System Grid Federation https://doi.org/10.22033/ESGF/CMIP6.9755 (2020).

53. Lee, W.-L. & Liang, H.-C. AS-RCEC TaiESM1.0 model output prepared for CMIP6 ScenarioMIP ssp245. Earth System Grid Federation https://doi.org/10.22033/ESGF/CMIP6.9808 (2020).

54. Lee, W.-L. & Liang, H.-C. AS-RCEC TaiESM1.0 model output prepared for CMIP6 ScenarioMIP ssp585. Earth System Grid Federation https://doi.org/10.22033/ESGF/CMIP6.9823 (2020).

55. Hauser, M., Engelbrecht, F. & Fischer, E. M. Transient global warming levels for CMIP5 and CMIP6. Zenodo https://doi.org/10.5281/ZENODO.3591806 (2022).
