# Supplementary material for: Robust intensification of projected regional precipitation extremes over Africa
Source: Nat Commun. 2026 May 16;17:6492. doi: 10.1038/s41467-026-73246-2 (PMC13377057; doi:10.1038/s41467-026-73246-2)
Supplement: Supplementary file 3 — Transparent Peer Review file [file 41467_2026_73246_MOESM3_ESM.pdf]

nature portfolio

Peer Review File

Robust intensification of projected regional precipitation extremes over Africa

Corresponding Author: Professor Akintomide Akinsanola

This file contains all reviewer reports in order by version, followed by all author rebuttals in order by version.

Version 0:

Reviewer comments:

Reviewer #1

(Remarks to the Author)
The paper is well written and covers the important topic of future changes in precipitation extremes over Africa and whether we can constrain projections using historical trends. However, I have a number of major concerns that would need to be fully addressed before I would consider the paper acceptable for publication in Nature Comms. These relate to the novelty of the paper and also to some of the methods employed.

Major comments
1. It is not clear to me that this provides a sufficient advance on our understanding of extreme precipitation changes to warrant publication in Nature Comms. There is a large volume of literature exploring changes in precipitation extremes and the role of thermodynamic changes (i.e. increasing atmospheric moisture with warming) in driving these. The authors need to be clear what the novelty is and in particular outline how this study provides a significant advance on previous studies.
2. The study uses trends in global mean surface temperature (GMST) to constrain future projections. Relationships between precipitation extremes and local temperature trends however were found to be statistically insignificant, so the authors need to provide a convincing argument on the mechanism by which global, but not local, temperature is related to local precipitation extremes. This mechanistic understanding is important to justify the assumption behind the constraints imposed – namely that a model's performance in representing global surface temperature in the historical climate has direct implications for projections of local rainfall extremes over Africa in the future.
3. Failing to explore other possible constraints, relating to the historical behaviour of variables other than GMST, feels like a significant omission. In particular, model uncertainty is found to dominate uncertainty in precipitation projections, with this largely associated with dynamical processes (related to model representation of sub-grid-scale processes notably convection, microphysics, and turbulence), and yet the work to produce constrained precipitation projections only explores historical warming trends which targets the thermodynamical component of the change. As a minimum, the authors need to provide justification for only exploring GMST constraints and discuss the implications of this choice related to findings from previous sections in the paper.
4. Although the main paper is well written, the methods themselves are not well explained. In particular just providing equations, with little explanation of the justification of the form these take, the underlying assumptions or acknowledgment of any caveats, is not sufficient. For example, for the emergent constraints the authors need to be clear on the assumptions, both the scientific basis of the constraint and any statistical assumptions (e.g. Gaussian distribution) and the implications of these.
5. Constraining projections through comparison with historical observations will only work if there are not systematic errors common to all models. Here CMIP6 models are being used to explore daily precipitation extremes, and in all cases the parameterisation of convection may lead to biases. It is likely that km-scale models may give different results (e.g. Kendon et al, 2019 Nature Comms https://doi.org/10.1038/s41467-019-09776-9), which constraining alone would not achieve unless km-scale models are included in the analysis from the outset. This is a caveat of this study that should be acknowledged and discussed.
6. For the thermodynamic and dynamic decomposition, the residual term is neglected with little evidence to support this, and yet this contains important non-linear variations and transient eddy effects. As a minimum, this residual term needs to be shown and explored within in the context of understanding evolving sources of uncertainty.

Minor comments
1. The paper is generally well written, but better links need to be made between sections, including how the understanding

from previous sections is relevant to the analysis and findings in later sections.
2. Line 56. This is not “as a result of” what was said in previous sentence. It is true that short duration precipitation extremes intensify more, but mechanisms for increases greater than Clausius Clapeyron should be explained here. These may be enhanced vertical motion, increased precipitation efficiency or an artefact of a shift in precipitation type to more convective.
3. Line 97. Need to be clear on the extent to which other studies have applied HEC approaches to constrain regional precipitation projections over Africa.
4. Line 111. Define RX1day on first use.
5. Line 171. Precipitation efficiency is only one factor, updraft strength is another. Fuller explanation of assumptions and caveats is needed here.
6. Line 273. A correlation coefficient of 0.6 means that only (0.6x0.6) 36% of the variance is explained by the TH component. So, although TH is a strong influence, it is not the only significant influence. This needs to be clearly conveyed.
7. Line 369. Would be good for the residual component of RX1day_scaling to also be shown and explored in this context.
8. Line 372. What are the implications of this for constraining future projections i.e. what is the possibility to reduce model uncertainty associated with dynamical process representation?
9. Line 380. This historical warming trend does not equate to climate sensitivity, and indeed many studies have shown that historical warming is not a strong constraint on the equilibrium response to greenhouse gas forcing.
10. Line 438 “steers projections toward more physically plausible outcomes” – what is the evidence for this statement?
11. Line 465. Flood risk is determined by many factors as well as precipitation extremes and size of population including catchment characteristics, antecedent conditions, flood defence measures and urban drainage systems, and infrastructure vulnerability. As a minimum, these points need to be acknowledged.
12. Line 655. The formulae here do not make sense and are missing terms. Even if there is no change in the population size, there would be an increase in exposure due to the increase in precipitation extremes. $\Delta Exp = Pop \times \Delta RX1day + \Delta Pop \times RX1day + \Delta Pop \times \Delta RX1day$, and similarly for GDP.
13. Line 719. Here do not provide satisfactory evidence to neglect eddy effects. Need to show this term over Africa as a minimum and explore within the uncertainty decomposition.
14. Line 783 and line 788, need to explain the adjustment factors and the forms these take. What are the underlying assumptions and caveats associated with this?
15. Line 796. Need to briefly explain the framework and calculations performed to do the decomposition here.

Signed: Professor Elizabeth Kendon

(Remarks on code availability)

Reviewer #2

(Remarks to the Author)
Summary and recommendation

The study examines projected changes in extreme precipitation over the African continent. Specifically, the authors quantify their future changes in CMIP6 projections, identify the underlying mechanisms of these changes, constrain uncertainties arising from inter-model differences, and assesses implications on exposure of population and GDP. The study finds that future increases in precipitation extremes are largest in Equatorial Africa where dynamic effects from diabatic heating in upward motion further amplify the spatially homogeneous thermodynamic influence. Extremely rare extremes are expected to occur more often towards the end of the 21st century. The study also uses a statistical method to reduce uncertainties arising from different warming trends across models, still resulting in an increase in extreme precipitation throughout much of the continent. Finally, the authors show that exposure of populations and assets will substantially increase as a result of extreme precipitation increases and population growth, specifically in urban areas, implying increased risk from hydrological hazards in the future.

Overall, I found the study very thorough, relevant, insightful, clearly structured, and well written. The manuscript covers a broad variety of aspects, from projected changes in extreme precipitation, to their thermodynamic and dynamic drivers, uncertainties stemming from inter-model differences, scenarios, and internal variability, to reducing these uncertainties using a statistical framework, and to quantifying the changes in exposure of population and assets. In my opinion, the manuscript is already in excellent shape. Below I list a few general and more specific comments which the authors may consider for improving the manuscript. I recommend publication of the manuscript after considering these suggestions.

General comments

1. Expression of projected precipitation changes. Already from the abstract it is clear that the study expresses the extreme precipitation changes in actual amounts. Have the authors considered expressing these in relative terms? I wonder whether this would give a more representative picture of extreme precipitation changes. When using actual values, these are almost automatically largest where the actual precipitation values are largest (e.g., Equatorial Africa). By contrast, relative extreme precipitation changes could be very large in regions with little precipitation too. Would adding a few figures in the supplement, similar as in the manuscript, but then expressing the changes in relative terms, be of added value to the study? This comment refers mostly to the first results section “Unconstrained projected precipitation extremes”.

2. Study region (e.g., Figure 1). I realized that the northern Africa, the zone northward of the Sahara domain, is not included in the study. I suppose because this region falls under the Mediterranean region according to the IPCC definition.

Nevertheless, I have the feeling that including this northern part of Africa would be rather important for the study, given its central focus on the African continent, especially given the large number of people living in this region, e.g., Morocco, Algeria, Tunisia, and Libya. Have the authors considered including this region in their study? Even if it would deviate from the commonly adopted IPCC definition, I feel that covering the part of northern Africa could make the study more complete.

Specific comments

Lines 61-74. The text summarizes here the atmospheric processes and variability patterns linked to extreme precipitation in different parts of Africa. However, it seems that northern Africa, including the Sahara and Mediterranean coast are missed, where extratropical forcing in the form of Rossby wave dynamics and surface (extratropical) cyclones are central to the occurrence of extreme precipitation. Adding a sentence on this may strengthen the manuscript.

Figure 1. The central panel of the figure shows a geographical map of Africa with topography. I wonder whether it would be more informative if the map would show characteristics of extreme precipitation, for example, the multi-model-mean extreme precipitation value of Rx1day or the multi-model mean change in extreme precipitation. This would give more insights in these characteristics or changes at the grid scale.

Line 201. Remove “better”?

Figure 2. Due to the used color bar in panels (a-d), as well as in several other figures, it is not easy to distinguish whether extreme precipitation increases or decreases if changes are relatively low as a result of the yellowish colors near zero values. Using a diverging color bar, perhaps with white color assigned to values near zero, may make the figures clearer.

Figure 2i,j. Could it be of added value to indicate statistical significance for changes in return periods in the different regions, for example, by adding hatching to the squares? Or are all changes in probability ratios significant anyways?

Lines 235-239 and Figure 2i,j. It took me a while to realize where in the figure I could find the numbers mentioned in the text. Would it be easier for a reader if the right-most column, corresponding to Africa, is emphasized by drawing a vertical line between this and the neighboring column to the left?

Line 276. Is it correct to attribute the decreased influence from the dynamics to “enhanced subsidence”? Wouldn’t it be more accurate to say that vertical motion / ascent is weaker during extreme precipitation?

Line 280-281. On what is the phrase “projected meridional contraction of the winter storm track” based? Isn’t it rather a weakening or poleward shift of the storm track that is relevant?

Line 281. Why is reference 36 included, which seems to relate to African easterly waves and not to winter storm track and cyclones?

Lines 315-316. I couldn’t exactly follow the phrase “and somewhat consistent but less under SSP2-4.5”.

Lines 327-329. Perhaps clarify that it is not the increase in 500-hPa geopotential height values itself, but rather a change in gradient that matters when speaking about a strengthened anticyclonic circulation. (spatially homogeneous increases in Z500 may be due to thermal expansion of the atmosphere and do not necessarily imply a strengthening of anticyclones). Showing the actual 500-hPa geopotential height contours under present-day climate conditions overlaid on future changes may help providing a reference of the actual circulation.

Lines 337-338. It is not clear to me where the inference “…are associated with weakened (enhanced) large-scale ascent and vertical moisture convergence” comes from.

Lines 349-351. In fact, Fig. 5ab,f, seem to show that contributions from the horizontal temperature advection term are nearly of similar magnitude as diabatic heating over northern Africa and western South Africa. This seems relevant as it suggests a weakening influence of midlatitude storm track, consistent with existing literature.

Lines 359-365. To me, Fig. 6 seems to tell that internal variability decreases with lead time whereas scenario uncertainty increases, but most of all, that model uncertainty dominates. The claim that model uncertainty becomes the dominant contributor by the end of the 21st century seems not to match Fig. 6, showing that this term actually decreases in some regions over the 2nd half of the 21st century, but dominates the overall uncertainty, independent of changes with lead time.

Lines 371-372. And what about internal variability? Is this source of uncertainty also associated with DY processes?

Line 373-375. What about misrepresentations of regional circulation patterns as a source of uncertainty?

Line 433. “consistently indicate larger increase in Rx1day”, larger, than what?

Line 467-468. Perhaps add a reference to the method section? At first, it wasn’t clear to me where the data on population exposure and GDP comes from.

Lines 512-513. The sentence seems to contrast fig. 8a,c showing that a greater number of people is exposed under SSP5-

8.5 than under SSP2-4.5, at least, for urban and total population.

Line 546. “subsidence-drive decreases” sounds a little odd to me since subsidence is usually linked to dry conditions. Perhaps speak about “reduced upward motion”?

Lines 549-550. Actually, the combination of Fig. 2a,c and Fig. 3b,d seems to suggest that the dynamic influence offsets the thermodynamic influence over northern Africa (including the Sahara) and western South Africa.

Line 559. Should “dynamical process” be “dynamical processes”?

Line 560. What about a possible role of regional circulation patterns?

Line 734. Add comma between “framework, which… “?

Line 766-767. Please, check the grammar of the sentences.

Lines 760-773. It is not entirely clear to me how I should interpret the different terms of the thermodynamic energy equation in relation to processes driving changes in the dynamics of precipitation extremes. Specifically, how should a reader interpret the local temperature tendency and horizontal temperature advection terms in this context?

Lines 701-719. Perhaps consider to make the use of acronyms more consistent by replacing “DYN” by “DY”?

(Remarks on code availability)

Version 1:

Reviewer comments:

Reviewer #1

(Remarks to the Author)
The authors have added important clarifications, additional analyses and better described methods and statistical assumptions. This has considerably improved the manuscript. However, there are a number of important underlying caveats to this analysis, which although acknowledged by the authors in their reviewer responses, are not explicitly stated in the manuscript. This needs to be addressed (see major comment below) but otherwise I am happy for the paper to be published.

Major comment:
The authors need to be clear that the method in this study is only constraining the large-scale atmospheric moistening. This is a key driver of future changes in precipitation extremes. However, there are other factors which will determine the actual realisation of greater large-scale atmospheric moisture in terms of precipitation extremes of the future. For example, local surface conditions are important for triggering convection and local dynamical processes for its development. Although it is not possible to constrain uncertainty arising from representation of these dynamical processes using observed trends and the HEC framework here, it is possible to assess the ability of models to capture dynamical variability and feedbacks through assessment of precipitation variability across a range of space and timescales in the present climate.
For confidence in the constrained projections, a key step forward would be to assess whether models not only represent GMST trends, but importantly also capture spatial and year-to-year variability in precipitation extremes. It maybe that all CMIP6 models are systematically underestimating changes in extremes, due to poor representation of local convective processes.
It is important that a clear statement of the caveats of this approach (namely that the constraint is based on large-scale atmospheric moisture only and the fact that only using CMIP6 models) is added to the manuscript, along with the implications for the reliability of the actual numbers quoted. Adding this statement does not undermine the value of the study – which is rather in the combined methodology employed and understanding gained – but rather gives important insights into future research directions.

Prof Lizzie Kendon, 20 April 2026

Reviewer #2

(Remarks to the Author)
After going carefully through the author responses to the raised comments and the revised manuscript, I feel that the revisions have adequately improved the manuscript. I have no further comments (except from a very minor one, listed below), and recommend acceptance of the manuscript in Nature Communications.

Lines 363-364. The phrase “These changes further enhance atmospheric moisture availability (Supplementary 363 Fig. S15a–b), thereby favoring more extreme precipitation events.” seems not obvious from the context. Strengthened subsidence would rather hinder extreme precipitation events.

Version 2:

Reviewer comments:

Reviewer #1

(Remarks to the Author)
The authors have not made any substantial changes to the manuscript in response to the 2nd review. The passages of text they point to in the Discussion were previously present in the earlier version of the manuscript. Therefore my previous review comments still hold, that the underlying caveats to this analysis have not been fully acknowledged in the manuscript. It is important that the caveats of this approach (namely that the constraint is based on large-scale atmospheric moisture only and the fact that only using CMIP6 models) are fully explained in the manuscript, along with the implications for the reliability of the actual numbers quoted.

Version 3:

Reviewer comments:

Reviewer #1

(Remarks to the Author)
Thank you for now sufficiently outlining the caveats of this approach, and implications for the reliability of the constrained projections.

I am happy for the paper to now be published.

There is a minor typo "convention" -> "convection" on line 740

Open Access This Peer Review File is licensed under a Creative Commons Attribution 4.0 International License, which permits use, sharing, adaptation, distribution and reproduction in any medium or format, as long as you give appropriate credit to the original author(s) and the source, provide a link to the Creative Commons license, and indicate if changes were made.
In cases where reviewers are anonymous, credit should be given to 'Anonymous Referee' and the source.
The images or other third party material in this Peer Review File are included in the article's Creative Commons license, unless indicated otherwise in a credit line to the material. If material is not included in the article's Creative Commons license and your intended use is not permitted by statutory regulation or exceeds the permitted use, you will need to obtain permission directly from the copyright holder.
To view a copy of this license, visit https://creativecommons.org/licenses/by/4.0/

Reviewer #1

Comment: *The paper is well written and covers the important topic of future changes in precipitation extremes over Africa and whether we can constrain projections using historical trends. However, I have a number of major concerns that would need to be fully addressed before I would consider the paper acceptable for publication in Nature Comms. These relate to the novelty of the paper and also to some of the methods employed.*

Response: We thank the reviewer for the positive assessment of the manuscript and for recognizing the importance of the topic. We acknowledge the concerns raised regarding the novelty of the study and aspects of the methodology. In the revised manuscript, we have carefully addressed these concerns. Below, we respond to each of the reviewer's specific comments in detail and describe the corresponding revisions made to the manuscript.

Major comments

Comment: *It is not clear to me that this provides a sufficient advance on our understanding of extreme precipitation changes to warrant publication in Nature Comms. There is a large volume of literature exploring changes in precipitation extremes and the role of thermodynamic changes (i.e. increasing atmospheric moisture with warming) in driving these. The authors need to be clear what the novelty is and in particular outline how this study provides a significant advance on previous studies.*

Response: We thank the reviewer for raising this important point. In our previous version, we acknowledged that numerous studies (mostly global in focus) have examined projected changes in precipitation extremes and highlighted the role of thermodynamic intensification associated with increasing atmospheric moisture due to warming. Our intention is not to revisit this well-established mechanism in isolation. Rather, this study advances previous work by integrating multiple complementary frameworks (statistical, physical, and socioeconomic) to provide a more comprehensive and constrained assessment of extreme precipitation changes, specifically for Africa.

Previous studies focusing on Africa have generally quantified projected changes in precipitation extremes or examined thermodynamic scaling relationships, but they rarely combine mechanistic attribution, formal uncertainty quantification, emergent constraints, and exposure assessment within a unified framework. To clarify this distinction, we have revised the Introduction and Discussion sections to more explicitly highlight the novelty and contributions of our study. In particular, this work provides a 4-tiered integration of statistical, physical, and socioeconomic analyses tailored to the African context:

1. **Hierarchical emergent constraint on regional precipitation extremes: While emergent constraints have been widely applied to quantities such as climate sensitivity or global-mean precipitation, their application to regional-scale daily precipitation extremes (Rx1day) over Africa remains very limited. Here, we develop a hierarchical emergent constraint (HEC) linking observed historical global warming trends to projected changes in Rx1day across African subregions. This framework narrows the**

spread of CMIP6 projections and provides observationally informed bounds on future extremes, reducing inter-model variance by up to ~35% in convectively active regions. In contrast to the broad envelopes of uncertainty typically reported in the literature, this approach yields more constrained and actionable projections relevant for regional climate-risk assessment. In addition, we addressed this under different GWLs

2. **Mechanistic attribution: Most studies stopped at TH or DY attribution. But here, we went further to assess the regional mechanism controlling TH by diagnosing the surface energy balance (SEB) budget and further assess mechanism of DY using the thermodynamic energy budget. This allows us to explicitly attribute TH to the intensification of net downwelling clear-sky longwave radiation (LWcs) and DY to net diabatic heating rate (Q1).**
3. **Quantification of the shift in return periods of rare extremes. A key result of this study is the quantification of how historically rare events evolve under warming. We show for example that 1-in-100-year precipitation events are projected to recur approximately every 2–14 years in many African regions by the end of the century. By explicitly characterizing the return periods of these rare events, this analysis provides a more intuitive representation of climate risk than changes in intensity alone.**
4. **Constrained projections and socioeconomic exposure. Finally, we translate both unconstrained and constrained projections of extreme precipitation into population and GDP exposure estimates across African subregions. This step connects physically based climate projections with societal risk, providing a policy-relevant assessment of potential impacts. Such integration of constrained climate projections with exposure metrics is still relatively uncommon in studies focused primarily on the physical drivers of precipitation extremes.**

Together, these elements help address a critical gap in literature. Africa remains one of the most under-studied regions regarding the physical drivers, uncertainties, and societal implications of extreme precipitation changes. By combining emergent constraints, mechanistic diagnostics, uncertainty attribution, and exposure analysis within a single framework, this study moves beyond documenting intensification of extremes to provide physically interpretable and observationally constrained projections of future risk. In addition, this study provides subregional insight.

To ensure that this contribution is clearer to readers, we have revised the final paragraph of the Introduction and expanded the Discussion section to explicitly emphasize these advances relative to previous studies. We hope these revisions better clarify how the present work advances current understanding of extreme precipitation changes over Africa.

Comment: *The study uses trends in global mean surface temperature (GMST) to constrain future projections. Relationships between precipitation extremes and local temperature trends however were found to be statistically insignificant, so the authors need to provide a convincing argument on the mechanism by which global, but not local, temperature is related to local precipitation extremes. This mechanistic understanding is important to justify the assumption behind the constraints imposed – namely that a model's performance in representing global surface*

temperature in the historical climate has direct implications for projections of local rainfall extremes over Africa in the future.

<u>Response</u>: We thank the reviewer for raising this important point regarding the physical interpretation of the emergent relationship used in the hierarchical emergent constraint (HEC). We agree that a clear mechanistic explanation is essential to justify the use of global mean surface temperature (GMST) as a predictor for regional precipitation extremes.

The rationale for using GMST rather than local temperature trends arises from the fact that the primary thermodynamic control on precipitation extremes operates through large-scale atmospheric moistening driven by global warming, rather than through local temperature changes alone. According to the Clausius–Clapeyron relationship, the water-holding capacity of the atmosphere increases by approximately 7% per degree of warming. Because the tropical atmosphere is dynamically well mixed, global-scale warming provides a robust first-order control on column-integrated moisture availability, which in turn influences the intensity of extreme precipitation events. As a result, models that simulate stronger historical global warming trend tend to exhibit larger increases in atmospheric moisture (see new Figure S20 below) and consequently larger intensification of precipitation extremes in future projections.

In contrast, local near-surface temperature trends are often weakly correlated with precipitation extremes, particularly in convectively active tropical regions such as Africa. Several factors contribute to this weak relationship. First, local temperature variability is strongly influenced by land–atmosphere feedbacks, cloud radiative effects, and evaporative cooling associated with precipitation events, which can decouple local temperature from the large-scale thermodynamic state of the atmosphere. Second, extreme precipitation events are primarily controlled by moisture convergence and vertical motion, which are governed by regional circulation dynamics rather than by local surface temperature alone. Consequently, local temperature trends do not necessarily provide a reliable proxy for the large-scale thermodynamic forcing that drives precipitation extremes.

Within this framework, GMST acts as an integrated measure of the global radiative forcing and associated large-scale atmospheric moistening, which is more directly linked to the thermodynamic component of precipitation intensification. This explains why the emergent relationship appears robust when using GMST trends but not when using local temperature trends. Our results support this interpretation: models with larger historical GMST trends systematically produce larger future increases in Rx1day across several African subregions. This behavior is consistent with previous emergent-constraint global studies that have linked global warming to changes in hydrological extremes through thermodynamic scaling mechanisms (e.g., Shiogama et al. 2022; Dai et al. 2024).

To clarify this point, we have revised the manuscript to better explain the physical basis of the GMST-based constraint.

"As a robust measure of large-scale thermodynamic forcing, GMST governs atmospheric moistening through Clausius–Clapeyron scaling, whereby near-saturation specific humidity, and thus extreme precipitation intensity, can increase by about 7% per degree of warming.

Consequently, models that simulate stronger historical GMST trends tend to exhibit larger increases in atmospheric water vapor (Supplementary Fig. S20) and, in turn, stronger projected intensification of precipitation extremes. GMST therefore serves as a more stable proxy for the large-scale thermodynamic forcing that governs atmospheric moisture availability and the intensity of precipitation extremes." (lines 431-438)

We hope that these clarifications help better justify the use of GMST as the predictor variable in the HEC framework and clarify the physical interpretation of the emergent relationship.

References:

Dai, P., Nie, J., Yu, Y., & Wu, R. (2024). Constraints on regional projections of mean and extreme precipitation under warming. *Proceedings of the National Academy of Sciences*, *121*(11), e2312400121. https://doi.org/10.1073/pnas.2312400121

Shiogama, H., Watanabe, M., Kim, H., & Hirota, N. (2022). Emergent constraints on future precipitation changes. *Nature*, *602*(7898), 612–616. https://doi.org/10.1038/s41586-021-04310-8

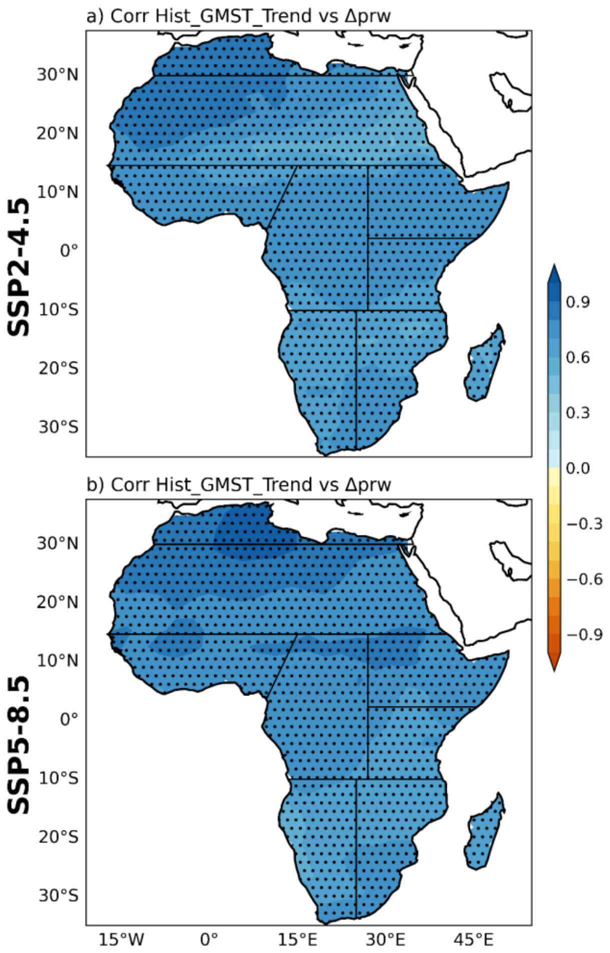

Figure S20. Intermodel correlation between historical (1985–2014) global mean surface temperature (GMST) trends and projected changes in column water vapor over Africa under the (a) SSP2-4.5 and (b) SSP5-8.5 scenarios. Stippling indicates grid points where correlations are statistically significant at the 95% confidence level based on a Student's t-test. Changes are calculated as the difference between the 2070–2099 and 1985–2014 means.

Comment: *Failing to explore other possible constraints, relating to the historical behaviour of variables other than GMST, feels like a significant omission. In particular, model uncertainty is found to dominate uncertainty in precipitation projections, with this largely associated with dynamical processes (related to model representation of sub-grid-scale processes notably convection, microphysics, and turbulence), and yet the work to produce constrained precipitation projections only explores historical warming trends which targets the thermodynamical component of the change. As a minimum, the authors need to provide justification for only exploring GMST constraints and discuss the implications of this choice related to findings from previous sections in the paper.*

Response: We thank the reviewer for this thoughtful comment. We agree that dynamical processes contribute substantially to the spread in precipitation projections and, therefore, the need to potentially consider constraints based on historical circulation changes/trends. However, we ultimately focused on GMST for two key reasons related to robustness and observational limitations.

First, the thermodynamic component of extreme precipitation is well understood and scales robustly with warming through Clausius–Clapeyron–driven increases in atmospheric moisture, which provides a physically grounded basis for using GMST as a predictor. Numerous studies have shown that extreme precipitation intensification is closely linked to warming-induced increases in near-saturation specific humidity (e.g., O'Gorman and Schneider, 2009; Allan et al., 2014; Pfahl et al., 2017). Because GMST reflects the strength of the externally forced warming signal in each model, it provides a stable proxy for the large-scale moistening that drives the thermodynamic component of precipitation extremes.

Second, identifying a robust historical constraint on the dynamical contribution (DY) is considerably more challenging. Dynamical changes in extreme precipitation are highly heterogeneous across regions and depend on processes such as convection, vertical motion, and circulation shifts that are strongly affected by internal variability and model representation of subgrid-scale processes. Most importantly, dynamical changes do not always scale consistently with thermodynamic forcing or follow a monotonic response to global warming, making them less predictable across models. For instance, numerous studies have documented that a wetter Sahel is associated with a strengthened Tropical Easterly Jet (TEJ), which enhances upper-level divergence and promotes deep convection and increased precipitation (e.g., Nicholson 2013). However, several studies, including ours, project a wetter Sahel by the end of the 21st century despite a weakening of the TEJ, implying that dynamical controls may become less influential under warming, with thermodynamic effects playing a more dominant role (e.g., Akinsanola and Zhou 2019a,b; Monerie et al. 2020).

Another limitation is that HEC requires historical observational input to accurately constraint and DY observations like vertical velocity over Africa are sparse/unavailable and uncertain. Although reanalysis products provide estimates of circulation fields, but these quantities are not directly observed and remain sensitive to model assumptions and data assimilation (e.g., Hersbach et al., 2020).

Following your comment, we evaluated the feasibility of a dynamical constraint by examining the relationship between historical trends in 500-hPa vertical velocity and projected changes in the dynamical contribution to Rx1day across models (see new Figure S27 below). Our results show that this relationship is weak and statistically insignificant, indicating that historical circulation trends cannot serve as a robust predictor of future dynamical changes in precipitation extremes over Africa. This lack of a strong intermodel relationship, combined with the limited observational reliability of vertical velocity trends, makes it difficult to construct a credible emergent constraint targeting the dynamic component.

For these reasons, the HEC framework in this study specifically targets the thermodynamic component of extreme precipitation change, where the physical linkage to warming is well established and the observational signal is robust.

More importantly, we note that the constraint presented herein can indirectly moderate part of the dynamical uncertainty because diabatic heating associated with latent heat release (quantified in our analysis through Q1 diagnostics) is partly controlled by atmospheric moisture availability, which itself scales with global warming.

We have clarified this rationale in the discussion section of the revised manuscript:

"We note that this constraint specifically targets the thermodynamic component of precipitation extremes, which is closely tied to global moisture availability and Clausius–Clapeyron scaling. While dynamic contributions dominate intermodel variance, constraining using historical circulation trends is challenging because regional dynamic processes over Africa are highly heterogeneous, observationally uncertain, and often dominated by internal variability. For example, historical trends in 500-hPa vertical velocity correlate only weakly with projected changes in the dynamic contribution (Supplementary Fig. S27), indicating insufficient predictive power for an emergent constraint. This complexity is illustrated in the Sahel, where wetter conditions have traditionally been linked to a strengthened Tropical Easterly Jet (TEJ) that enhances upper-level divergence and deep convection[98], yet future projections show increased precipitation despite a weakening TEJ, suggesting a reduced role of dynamic controls relative to thermodynamic forcing under warming[99]. Nonetheless, the HEC may indirectly moderate some dynamic uncertainty because the diabatic heating that drives vertical motion is partially dependent on the available moisture, which itself is constrained by GMST." (lines 666-679)

<u>References</u>:

Nicholson, Sharon E., The West African Sahel: A Review of Recent Studies on the Rainfall Regime and Its Interannual Variability, International Scholarly Research Notices, 2013, 453521, 32 pages, 2013. https://doi.org/10.1155/2013/453521

Akinsanola, A.A., Zhou, W. Projection of West African summer monsoon rainfall in dynamically downscaled CMIP5 models. ***Clim Dyn*** **53, 81–95 (2019a). https://doi.org/10.1007/s00382-018-4568-6**

Akinsanola, A.A., Zhou, W. Ensemble-based CMIP5 simulations of West African summer monsoon rainfall: current climate and future changes. ***Theor Appl Climatol*** **136, 1021–1031 (2019b). https://doi.org/10.1007/s00704-018-2516-3**

Monerie, PA., Wainwright, C.M., Sidibe, M. ***et al.*** **Model uncertainties in climate change impacts on Sahel precipitation in ensembles of CMIP5 and CMIP6 simulations.** ***Clim Dyn*** **55, 1385–1401 (2020). https://doi.org/10.1007/s00382-020-05332-0**

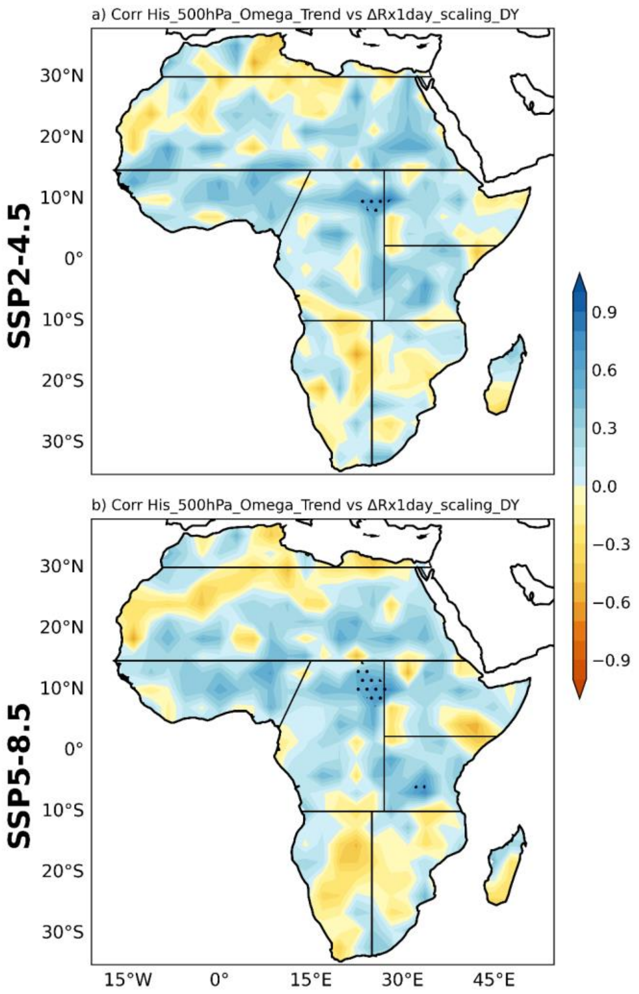

Figure S27. Intermodel correlation between historical (1985–2014) 500 hPa vertical velocity (omega) trends and projected changes in the dynamic (DY) component of Rx1day_scaling over Africa under the (a) SSP2-4.5 and (b) SSP5-8.5 scenarios. Stippling indicates grid points where

correlations are statistically significant at the 95% confidence level based on a Student's t-test. Changes are calculated as the difference between the 2070–2099 and 1985–2014 means.

Comment: *Although the main paper is well written, the methods themselves are not well explained. In particular just providing equations, with little explanation of the justification of the form these take, the underlying assumptions or acknowledgment of any caveats, is not sufficient. For example, for the emergent constraints the authors need to be clear on the assumptions, both the scientific basis of the constraint and any statistical assumptions (e.g. Gaussian distribution) and the implications of these.*

Response: We thank the reviewer for this important suggestion. We agree that the description of the hierarchical emergent constraint (HEC) framework should more clearly explain the physical rationale for the constraint, as well as the statistical assumptions and potential caveats. In the revised manuscript, we have expanded the Methods section to clarify (i) the scientific basis for using the historical global warming trend as a predictor of future precipitation extremes, (ii) the Bayesian formulation underlying the HEC framework, and (iii) the statistical assumptions involved, including the Gaussian approximation and the interpretation of the resulting constrained distributions. We also briefly acknowledge limitations of the approach, including potential sensitivity to observational uncertainty and the assumption of a linear intermodel relationship between the predictor and the projected response.

"Emergent constraints on projected extreme precipitation: To constrain the projected Rx1day over Africa, we apply the HEC framework proposed by Bowman et al. (2018)[58] and widely used in recent studies. The HEC framework builds on the concept of emergent constraints, which uses physically interpretable relationships across climate models between an observable aspect of the present-day climate and a future climate response. In this study, we use the historical global mean surface temperature (GMST) trend as the predictor variable because it captures intermodel differences in the large-scale thermodynamic response to warming, which influences precipitation extremes. The HEC approach provides a Bayesian formulation that accounts for uncertainties in both model simulations and observations. Unlike classical emergent-constraint methods based on simple regression, the hierarchical framework explicitly incorporates observational uncertainty and the strength of the intermodel relationship between the predictor and projected response. Under this framework, the constrained projections represent a probabilistic estimate of the future response conditioned on the observed value of the predictor variable.

Following Bowman et al. (2018)[58], constrained projections of extreme precipitation changes are assumed to follow a Gaussian distribution, characterized by a mean and variance. This assumption provides a good approximation for describing the intermodel spread, although deviations from normality may occur in some ensembles.

The mean of the constrained future projections is defined as:

$$\mu_{z|y} = \mu_z + \frac{\rho\sigma_z\sigma_x}{\sigma_x^2+\sigma_y^2}\left(\mu_y - \mu_x\right) \qquad (13)$$

where x and z denote the historical simulations and future projections, respectively, and y denotes observations. The symbols μ and σ refer to the mean and standard deviations, respectively, and ρ is the intermodel correlation coefficient between x and z.

The adjustment factor $\frac{\rho\sigma_z\sigma_x}{\sigma_x^2+\sigma_y^2}$ determines how strongly observational information modifies the unconstrained model mean. This factor increases when the intermodel correlation between the predictor and predictand is strong and when observational uncertainty is small relative to the spread of model simulations. Conversely, when observational uncertainty is large or the intermodel relationship is weak, the adjustment factor becomes small, and the constrained projection approaches the original multimodel mean.

The variance of the constrained future projections is computed as:

$$\sigma_{z|y}^2 = \left(1 - \frac{\rho^2}{1+\left(\frac{\sigma_x^2}{\sigma_y^2}\right)^{-1}}\right)\sigma_z^2 \qquad (14)$$

which reflects the reduction in projection uncertainty obtained by conditioning on observational information. Both unconstrained and constrained projections are assumed to be approximately Gaussian. The relative reduction of variance (RRV) is then calculated as:

$$RRV = \left(1 - \frac{\sigma_{z|y}^2}{\sigma_z^2}\right) \times 100\% \qquad (15)$$

This metric quantifies the extent to which observational information reduces projection uncertainty.

The HEC framework relies on several key assumptions. First, it assumes an approximately linear relationship across models between the historical predictor and the future response. Second, uncertainties are assumed to be approximately Gaussian, allowing the constrained distribution to be described by its mean and variance. Third, the observational estimate is assumed to be an unbiased representation of the true climate state. Deviations from these assumptions, including nonlinear predictor–predictand relationships, structural model errors, or underestimated observational uncertainties, may influence the strength of the constraint. Therefore, the robustness of the emergent constraint depends on the physical plausibility of the predictor–predictand relationship and the reliability of the observational dataset used to constrain the projections. " **(lines 943-987)**

Comment: *Constraining projections through comparison with historical observations will only work if there are not systematic errors common to all models. Here CMIP6 models are being used to explore daily precipitation extremes, and in all cases the parameterisation of convection may lead to biases. It is likely that km-scale models may give different results (e.g. Kendon et al, 2019 Nature Comms https://doi.org/10.1038/s41467-019-09776-9), which constraining alone would*

not achieve unless km-scale models are included in the analysis from the outset. This is a caveat of this study that should be acknowledged and discussed.

<u>Response</u>: We thank the reviewer for raising this important point regarding the limitations associated with convection parameterization in CMIP6 models. We fully agree that all CMIP6 models rely on parameterized convection, which can influence the simulation of precipitation extremes. Convection-permitting (CP) models operating at kilometer-scale resolution can explicitly resolve deep convective processes and therefore provide a more physically realistic representation of convective rainfall. For instance, our studies over the US (Akinsanola et al., 2024) show that convection-permitting simulation improves representation of extreme precipitation.

More importantly, studies using convection-permitting simulations over Africa (e.g., Kendon et al., 2019) show that explicitly resolving convection can lead to stronger increases in short-duration precipitation extremes compared with parameterized-convection models, suggesting that conventional climate models may underestimate the magnitude of future changes in rainfall extremes. For example, the CP4-Africa simulations at 4.5 km resolution show substantially larger increases in extreme rainfall intensity compared with a convection-parameterized regional model, highlighting the role of explicitly resolved convective dynamics in amplifying rainfall extremes.

While the CP is advantageous and represents a notable future direction, CP simulations remain computationally expensive and are currently available only for limited ensembles and regional domains, typically covering relatively short simulation periods. As a result, they cannot yet provide the large multi-model ensembles required for emergent-constraint analysis or for robust uncertainty quantification across emission scenarios. In contrast, CMIP6 provides the large ensemble of models necessary to identify intermodel relationships and apply hierarchical emergent constraints, and to systematically assess model agreement and quantify uncertainties across models and scenarios in a statistically robust framework.

Nevertheless, the direction of projected change presented in our study is consistent to those reported in studies using convection-parameterized and convection-permitting simulations, with both frameworks projecting increasing precipitation extremes under warming. Convection-permitting models generally show equal or slightly stronger intensification, which suggests that our CMIP6-based projections may represent a conservative estimate of future intensification. In this context, the emergent constraint applied here primarily narrows the uncertainty within the CMIP6 ensemble but does not address structural biases that may arise from the common use of convection parameterizations.

This limitation is now discussed in the revised manuscript.

"It is important to note that all CMIP6 models used in this study rely on parameterized convection, which may influence the representation of short-duration precipitation extremes. Convection-permitting (kilometer-scale) simulations that explicitly resolve deep convective processes often produce stronger intensification of rainfall extremes compared with convection-parameterized models. For example, high-resolution simulations over Africa show that explicitly resolving convection can substantially increase projected changes in extreme precipitation intensity relative to parameterized models[46]. While such simulations

provide valuable insights into convective processes, they are currently limited in spatial coverage, ensemble size, and simulation length, making them difficult to use in emergent-constraint frameworks that require large multimodel ensembles. The consistent direction of change between CMIP6 models and convection-permitting simulations, both projecting stronger precipitation extremes under warming, provides confidence in the qualitative signal reported herein. Also, the CMIP6 models used herein allow us to systematically assess model agreement and quantify uncertainties across models and scenarios in a statistically robust framework. However, because convection is not explicitly resolved in CMIP6 models, the constrained projections presented in this study should be interpreted as a likely conservative estimate, or lower bound, of future intensification of precipitation extremes across Africa. Future studies combining large-ensemble convection-permitting simulations with constraint frameworks may further refine these projections as such datasets become more widely available. " (lines 722-740)

References:
Akinsanola, A.A., Jung, C., Wang, J. *et al.* Evaluation of precipitation across the contiguous United States, Alaska, and Puerto Rico in multi-decadal convection-permitting simulations. *Sci Rep* 14, 1238 (2024). https://doi.org/10.1038/s41598-024-51714-3

Comment: *For the thermodynamic and dynamic decomposition, the residual term is neglected with little evidence to support this, and yet this contains important non-linear variations and transient eddy effects. As a minimum, this residual term needs to be shown and explored within in the context of understanding evolving sources of uncertainty.*

Response: We thank the reviewer for this important suggestion. The residual term, which represents nonlinear interactions and transient eddy contributions in the scaling decomposition, has now been explicitly included and analyzed. We have added a new figure (Supplementary Fig. S9; shown below) that illustrates the spatial distribution of the residual component under both SSP2-4.5 and SSP5-8.5 scenarios.

Our analysis shows that the residual contribution remains relatively small compared with the thermodynamic and dynamic components. Across most African regions, the residual term accounts for about 5% of the total change in Rx1day, indicating that nonlinear interactions and transient eddy effects play a small role in the large-scale intensification of precipitation extremes in the CMIP6 ensemble. This behavior is consistent with previous studies showing that the thermodynamic and large-scale dynamic contributions dominate extreme precipitation scaling, while higher-order nonlinear terms are generally smaller at continental scales (e.g., O'Gorman and Schneider, 2009; Pfahl et al., 2017).

We acknowledge that the residual term may contain contributions from nonlinear moisture–dynamics interactions and transient eddies, which can influence extreme precipitation locally. However, robustly diagnosing these processes typically requires higher temporal and spatial resolution datasets capable of resolving mesoscale and synoptic transient variability, which are not consistently available across the full CMIP6 multi-model ensemble used in this study. Such analyses are therefore better suited to high-resolution or convection-permitting model experiments.

Given the relatively small magnitude of the residual contribution across the ensemble and the lack of consistent high-resolution diagnostics across all models, we focus the primary decomposition on the thermodynamic and dynamic components, while now documenting the residual contribution in the Supplementary Material. The manuscript has been updated to clarify this point and to reference the new figure.

"The residual term, representing nonlinear interactions and transient eddy contributions, is also diagnosed but is not discussed in detail, as it accounts for only ~5% of the total change across most regions (Supplementary Fig. S9). Also, robust quantification of the mechanism modulating these nonlinear and transient processes would require higher temporal resolution data than are available from the CMIP6 models used herein. " (lines 282-287)

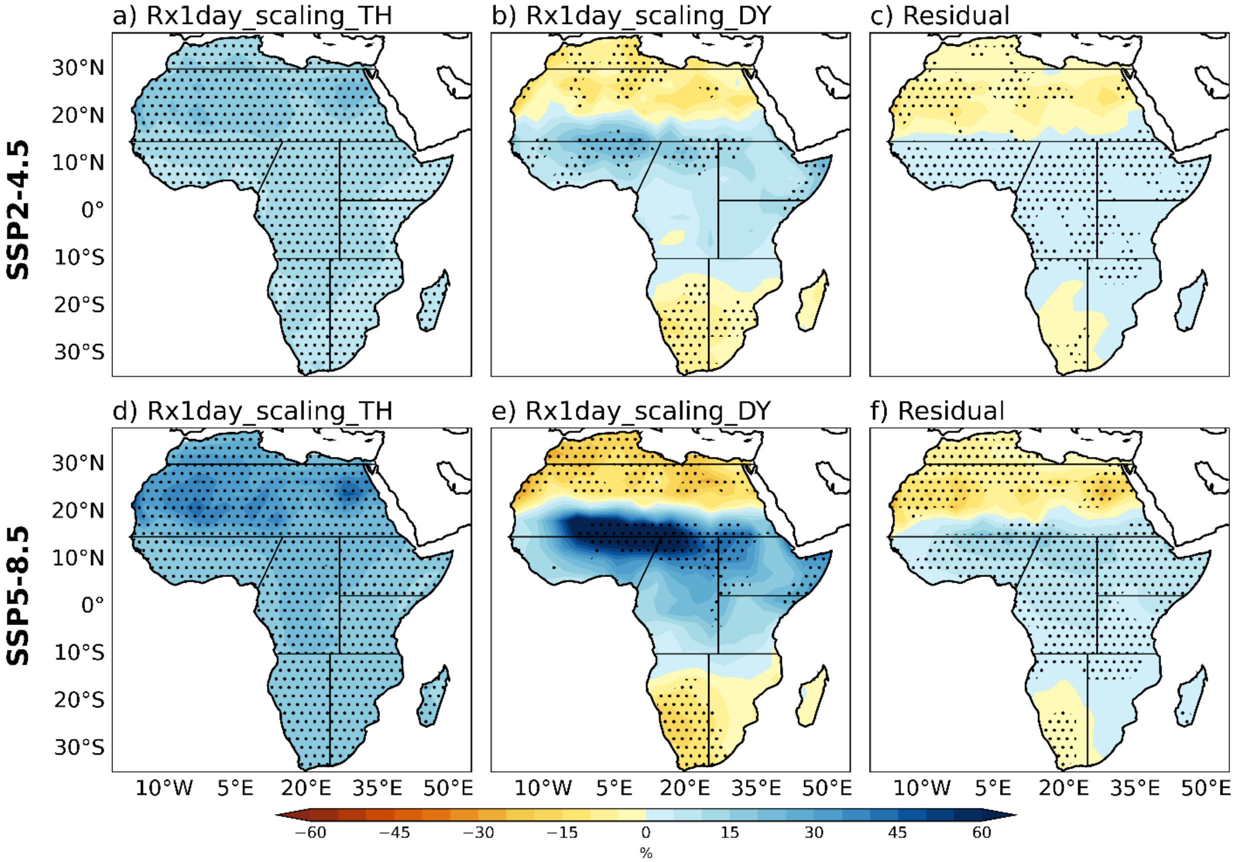

Figure S9. Ensemble-mean (EnsMean) changes (%) in thermodynamic, dynamic, and residual scaling under the (a-c) SSP2-4.5 and (d-f) SSP5-8.5 scenarios. Stippling denotes regions where at least 70% of models agree on the sign of EnsMean. Changes are calculated as the relative difference between the 2070–2099 and 1985–2014 means.

Minor comments

Comment: *The paper is generally well written, but better links need to be made between sections, including how the understanding from previous sections is relevant to the analysis and findings in later sections.*

Response: We thank the reviewer for this helpful suggestion. In the revised manuscript, we strengthened the narrative continuity across sections by adding explicit transitional sentences that clarify how the results from earlier analyses motivate and inform the subsequent sections.

Comment: *Line 56. This is not "as a result of" what was said in previous sentence. It is true that short duration precipitation extremes intensify more, but mechanisms for increases greater than Clausius Clapeyron should be explained here. These may be enhanced vertical motion, increased precipitation efficiency or an artefact of a shift in precipitation type to more convective.*

Response: We thank the reviewer for this important clarification. We agree that the wording suggested an overly direct causal relationship between the energetic constraint on global-mean precipitation and the stronger scaling of short-duration precipitation extremes. In the revised manuscript, we have rephrased this passage to remove the causal phrasing and to more clearly explain the physical mechanisms that can produce scaling rates at or above the Clausius–Clapeyron (CC) rate. Specifically, we now note that super-CC scaling may arise from enhanced vertical motion, increased precipitation efficiency, or shifts toward more convective precipitation regimes. These mechanisms are consistent with previous theoretical and observational studies of extreme precipitation scaling (e.g., O'Gorman and Schneider, 2009; Lenderink and van Meijgaard, 2008; Pfahl et al., 2017). The revised text better reflects the physical processes underlying departures from ideal CC scaling.

"In response to global warming, global-mean precipitation is energetically constrained and is projected to increase more slowly, at roughly 2–3% per degree Celsius[26,28], whereas short-duration precipitation extremes are more directly controlled by atmospheric moisture availability and vertical motion, and therefore often intensify at rates approaching or exceeding CC scaling. Scaling rates exceeding CC can arise from several mechanisms, including enhanced vertical motion during extreme events, increased precipitation efficiency, or shifts toward more convective precipitation regimes[29-31]. These processes highlight the important role of both thermodynamic moistening and dynamic amplification in shaping changes in precipitation extremes[3,5,32,33]." (lines 54-62)

References:

Lenderink, G., & van Meijgaard, E. (2008). ***Increase in hourly precipitation extremes beyond expectations from temperature changes.*** **Nature Geoscience. https://doi.org/10.1038/ngeo262**

O'Gorman, P. A., & Schneider, T. (2009). ***The physical basis for increases in precipitation extremes in simulations of 21st-century climate change.*** **PNAS. https://doi.org/10.1073/pnas.0907610106**

Pfahl, S., O'Gorman, P. A., & Fischer, E. M. (2017). ***Understanding the regional pattern of projected future changes in extreme precipitation.*** **Nature Climate Change. https://doi.org/10.1038/nclimate3287**

Comment: *Line 97. Need to be clear on the extent to which other studies have applied HEC approaches to constrain regional precipitation projections over Africa.*

Response: We appreciate the reviewer's comment. Although emergent constraints have been applied extensively to narrow uncertainties in global precipitation extremes (e.g., Shiogama et al., 2022; Zhang et al., 2025), our study represents, to our knowledge, the first use of HEC directed specifically at regional precipitation extremes over Africa, a distinction we have emphasized in the final paragraph of the introduction in the revised manuscript.

References:

Shiogama, H., Watanabe, M., Kim, H. *et al.* Emergent constraints on future precipitation changes. *Nature* 602, 612–616 (2022). https://doi.org/10.1038/s41586-021-04310-8

Zhang, Q. et al. Projections of climate change and its impacts based on CMIP6 models—calling attention to quantifying and constraining uncertainty. Environ. Res. Lett. 20, 031002 (2025). https://doi.org/10.1088/1748-9326/adb1f7

Comment: *Line 111. Define Rx1day on first use.*

Response: We thank the reviewer for pointing this out. We have revised the manuscript to define Rx1day at its first occurrence to improve clarity for readers who may not be familiar with the index.

Comment: *Line 171. Precipitation efficiency is only one factor, updraft strength is another. Fuller explanation of assumptions and caveats is needed here.*

Response: We thank the reviewer for this helpful suggestion. In the revised manuscript, we have expanded the discussion to acknowledge these additional dynamic factors and to clarify that the scaling approach represents a simplified diagnostic that does not explicitly resolve all processes controlling precipitation extremes. We now explicitly note that departures between the scaling estimate and simulated Rx1day may arise from variations in precipitation efficiency, vertical velocity (updraft strength), and other nonlinear processes associated with convective dynamics.

"Although Rx1day_scaling yields slightly lower intensities than the simulated Rx1day (Supplementary Fig. S2f), particularly across equatorial Africa, this discrepancy likely reflects simplifying assumptions inherent in the scaling framework. In particular, the diagnostic does not explicitly account for variations in precipitation efficiency[31] or changes in updraft strength (vertical velocity) during extreme events, both of which can enhance condensation rates and precipitation intensity[30,31]. Additional nonlinear processes associated with convective organization and mesoscale dynamics may also contribute to departures between the scaling estimate and fully simulated extremes, highlighting an important caveat when interpreting precipitation-scaling diagnostics." (lines 176-184)

Comment: *Line 273. A correlation coefficient of 0.6 means that only (0.6x0.6) 36% of the variance is explained by the TH component. So, although TH is a strong influence, it is not the only significant influence. This needs to be clearly conveyed.*

Response: We thank the reviewer for this important clarification. We have revised the text to explicitly state that while TH processes exert a strong influence on the spatial distribution of precipitation extremes, they do not fully explain the variability, and other factors, particularly dynamical processes, also play a significant role.

To further investigate this point, we performed a spatial intermodel regression analysis between Rx1day_scaling and the TH component (see new Figure S12 shown below). The results confirm that TH processes explain a substantial fraction of the spatial variability across most African regions. These results reinforce the interpretation that thermodynamic moistening provides the primary large-scale driver of intensification, while regional circulation changes and associated dynamical processes contribute importantly to spatial heterogeneity in precipitation extremes. The manuscript text has been revised accordingly to reflect this more nuanced interpretation.

"the pattern correlation between Rx1day_scaling and the TH component exceeds 0.6 in most regions, indicating that thermodynamic processes explain a substantial fraction of the spatial variability in projected precipitation extremes. However, this relationship also implies that a considerable portion of the variance remains unexplained by TH alone, highlighting the important contribution of dynamic processes and other regional factors in shaping the spatial distribution of Rx1day changes. Additional regression analysis further shows that the TH contribution is strongest across most African regions (Supplementary Fig. S12)." (lines 298-304)

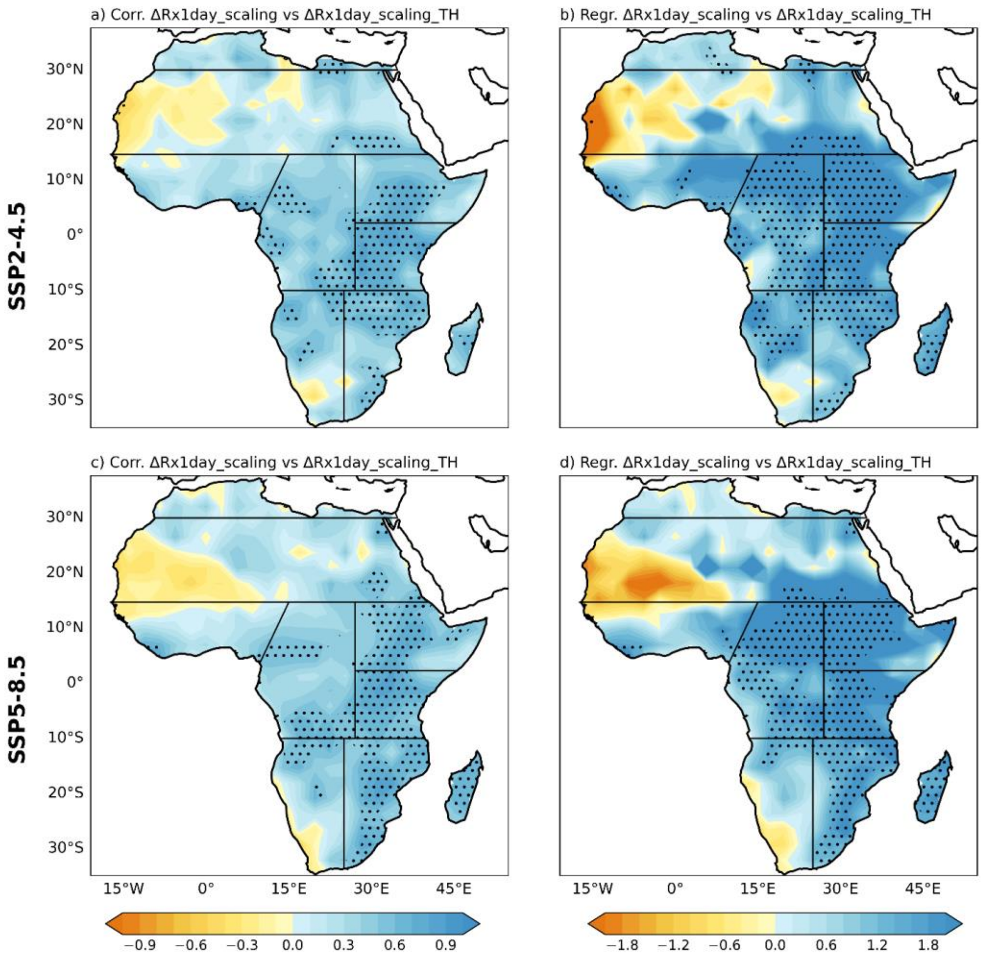

Figure S12. (a, c) Intermodel correlation and (b, d) regression between changes in Rx1day_scaling and its dynamic component under the (a, b) SSP2-4.5 and (c, d) SSP5-8.5 scenarios. Stippling indicates grid points where correlations/regressions are statistically significant at the 95% confidence level based on a Student's t-test. Changes are calculated as the difference between the 2070–2099 and 1985–2014 means.

Comment: *Line 369. Would be good for the residual component of Rx1day_scaling to also be shown and explored in this context.*

<u>Response</u>: We thank the reviewer for raising this point. Following our previous response, Figure S9 presents additional analysis confirming that the residual component of Rx1day_scaling has a minimal contribution. Given this, we chose not to include it in subsequent analyses.

Comment: *Line 372. What are the implications of this for constraining future projections i.e. what is the possibility to reduce model uncertainty associated with dynamical process representation?*

Response: We thank the reviewer for raising this important point. Because model uncertainty is largely associated with dynamical processes (DY), which depend strongly on the representation of subgrid-scale processes such as convection, cloud microphysics, and turbulence, the potential for directly constraining these DY processes using historical observations is limited by the availability of observed data of dynamical variables over Africa. Furthermore, large-scale circulation changes over Africa are often weak in the historical record and strongly influenced by internal variability, making robust observational constraints on dynamic responses difficult to establish. Consequently, the emergent constraint framework applied in this study primarily targets the thermodynamic component, which exhibits a clearer and more robust relationship with global warming. Importantly, as discussed earlier, constraining TH can also indirectly constrain DY, since thermodynamic changes influence atmospheric stability, moisture availability, and latent heating, which in turn modulate circulation patterns and vertical motion. We have revised the manuscript to address this comment:

"We note that the potential for directly constraining these DY processes using historical observations is limited by the availability of observed data for dynamic variables over Africa. Furthermore, large-scale circulation changes over Africa are often weak in the historical record and strongly influenced by internal variability. This limits the possibility of directly reducing model uncertainty associated with dynamic process representation through observational constraints alone. Consequently, the emergent-constraint framework applied in this study targets primarily the TH component. In addition, given that TH processes dominate the forced signal of precipitation extremes, this understanding motivates the use of an observationally informed emergent constraint based on historical global warming trends to reduce projection uncertainty." (lines 415-424)

Comment: *Line 380. This historical warming trend does not equate to climate sensitivity, and indeed many studies have shown that historical warming is not a strong constraint on the equilibrium response to greenhouse gas forcing.*

Response: We thank the reviewer for this important clarification. We agree that the historical warming trend should not be interpreted as equivalent to equilibrium climate sensitivity (ECS). In this study, the historical global mean surface temperature (GMST) trend is not used as a proxy for ECS, but rather as an empirical indicator of the strength of the forced warming response across models over the historical period, which influences large-scale atmospheric moistening and therefore extreme precipitation scaling. We have revised the manuscript to remove the wording inferring historical warming with climate sensitivity and to clarify that the GMST trend is used here as a statistical predictor that captures intermodel differences in the magnitude of historical warming.

"The GMST trend over 1985–2014 is used as the predictor for our HEC (see Methods). It is important to note that the GMST trend is employed here strictly as a statistical predictor that captures intermodel differences in large-scale thermodynamic response, rather than as a mechanistic or causal driver of regional precipitation extremes." (lines 442-446)

Comment: *Line 438 "steers projections toward more physically plausible outcomes" – what is the evidence for this statement?*

Response: We thank the reviewer for pointing this out. We have revised the sentence, and the revised text therefore focuses on the statistically demonstrated reduction in projection spread rather than implying an independent assessment of physical plausibility.

"Even in these regions, however, the emergent constraint still narrows the projected distributions and slightly reduces the spread of projected outcomes." (lines 493-494)

Comment: *Line 465. Flood risk is determined by many factors as well as precipitation extremes and size of population including catchment characteristics, antecedent conditions, flood defence measures and urban drainage systems, and infrastructure vulnerability. As a minimum, these points need to be acknowledged.*

Response: We thank the reviewer for this important clarification. In the revised manuscript, we have expanded the relevant sentence to explicitly acknowledge the role of catchment characteristics, antecedent soil moisture conditions, flood protection infrastructure, urban drainage capacity, and infrastructure vulnerability in shaping flood impacts. These additions better contextualize the role of precipitation extremes as one of several interacting drivers of flood risk. The revised text now reads as:

"Increases in Rx1day can intensify flood risk, disrupt infrastructure, and undermine socioeconomic development, making robust assessments of future changes essential. We note that flood risk is also shaped by multiple interacting factors, including catchment characteristics, antecedent soil moisture conditions, flood defense measures, urban drainage capacity, and the vulnerability of built infrastructure, in addition to precipitation extremes and population exposure[13,91,92]." (lines 519-524)

Comment: *Line 655. The formulae here do not make sense and are missing terms. Even if there is no change in the population size, there would be an increase in exposure due to the increase in precipitation extremes.* $\Delta Exp = Pop \times \Delta Rx1day + \Delta Pop \times Rx1day + \Delta Pop \times \Delta Rx1day$*, and similarly for GDP.*

Response: We thank the reviewer for carefully examining this section and for pointing out the issue with the formulation. We agree that the original expression was incomplete, and the previous analysis inadvertently considered only the interaction component, without fully accounting for the contributions from changes in population/GDP or changes in Rx1day.

Based on the existing literature and the reviewer's suggestion, we revised the analysis to decompose exposure change as: $\Delta Exp=Pop\times\Delta Rx1day+\Delta Pop\times Rx1day+\Delta Pop\times\Delta Rx1day$ **same for GDP. This allows us to quantify total exposure as well as the individual contributions from the climate effect (ΔRx1day), the social effect (changes in population/GDP), and the interaction effect.**

To summarize, most components contribute positively to exposure. However, for the rural population under SSP585, the social and interaction effects are negative due to rapid urbanization and migration, resulting in a decline in total exposure for rural populations.

We also carefully rechecked all other analysis to ensure there were no additional errors or misrepresentations and confirmed that the updated calculations are correct. The revised discussion and Figure are provided below.

"Exposure of population and GDP: The NASA Socioeconomic Data and Applications Center (SEDAC) provides projected gridded population data following SSP pathways at 1/8° resolution, with the year 2000 as the baseline[111]. Similarly, Murakami et al. (2021)[112] provide the projected GDP across SSP scenarios at 1/12° resolution. Using these datasets, we evaluate changes in population and GDP exposure for each grid cell and the relative importance of different factors in driving these changes[113,114]. These factors include the climate, population/GDP, and interaction effects.

$$\Delta Exposure_{POP-Rx1day} = \overline{Pop} \times \Delta Rx1day + \Delta Pop \times \overline{Rx1day} + \Delta Pop \times \Delta Rx1day \quad (1)$$

$$\Delta Exposure_{GDP-Rx1day} = \overline{GDP} \times \Delta Rx1day + \Delta GDP \times \overline{Rx1day} + \Delta GDP \times \Delta Rx1day \quad (2)$$

Here, Δ represents the mean change between the future period (2070–2099) and the historical period (1985–2014), and the overbar represents the mean during the historical period. For the population/GDP, the year 2000 was chosen as the historical period rather than a multiyear mean because socioeconomic data are available at decadal intervals, and the 2010 data are projections under the SSP scenarios rather than observations. The climate effect is defined as the impact of the Rx1day change on the historical population/GDP (1st term in the equation). The population/GDP effect is the impact of changes in population/GDP relative to historical Rx1day (2nd term). The interaction effect accounts for changes in both population/GDP and Rx1day (3rd term).**" (lines 785-801)**

"Our results show that model constraints consistently moderate the projected impacts. Decomposition of total exposure into climate, population/GDP, and interaction effects reveals that population/GDP changes are the dominant driver, followed by the interaction effect, with the climate effect being the smallest contributor, an order of magnitude lower than the others (Supplementary Figs. S24-26). The climate effect contributes positively across all regions, except for a minor negative contribution in the MED region, consistent with the decrease in Rx1day also noted in the relative change analysis (Supplementary Fig. S24). Urban populations experience substantial increases in exposure under both SSP2-4.5 and SSP5-8.5, regardless of whether projections are constrained or unconstrained. WAF exhibits the largest urban exposure, increasing from approximately 3.61×10^{10} person mm day^{-1} under SSP2-4.5 to nearly 3.75×10^{10} under SSP5-8.5 in the unconstrained projections (Fig 8a). The constrained EnsMean shows lower increases, around 3.35×10^{10} and 3.30×10^{10} person mm day^{-1}, respectively. CAF, NEAF, SEAF, and ESAF also show moderate increases, from about 0.5×10^{10} to nearly 1.1×10^{10} person mm day^{-1}. Increases in MED, SAH, WSAF, and MDG are relatively smaller but consistent with their population sizes. These upward trends indicate that both Rx1day intensification and demographic growth, reflected in the large population and interaction effects, contribute to increasing urban exposure across all subregions (Fig. 8a, and Supplementary Figs. S25a, S26a).

On the other hand, rural population exposure shows modest increases under SSP2-4.5 but declines under SSP5-8.5. WAF exhibits the largest negative rural exposure, approximately -0.33×10^{10} person mm day^{-1} in the unconstrained SSP5-8.5 projections and -0.38×10^{10} under constraints (Fig. 8b). CAF, NEAF, SEAF, and ESAF also show modest negative rural exposure up to -0.2×10^{10} in the unconstrained and constrained SSP5-8.5 scenario. Decomposition analysis clarifies that this rural decline is driven primarily by the population effect, specifically the projected decrease

in rural population due to high migration and urbanization under SSP5-8.5[93], with the interaction effect compounding the reduction, while the climate effect remains positive (Supplementary Figs. S24-26b). These contrasting magnitudes and directions of change in urban and rural exposure influence total exposure. Because urban exposure is consistently positive and increases by several tens of billions of person mm day^{-1}, whereas rural exposure decreases by only a few billion, total regional averages closely follow urban trends. Even in regions where rural averages are strongly negative, such as WAF and ESAF, the magnitude of rural declines is much smaller than the increase in urban exposure. Consequently, total population exposure continues to rise under both emissions pathways. Under SSP2-4.5, Africa-wide total exposure reaches approximately 7.16 × 10^{10} person mm day^{-1} in the unconstrained ensemble and ~6.60 × 10^{10} under constraints. Under SSP5-8.5, the unconstrained total falls to 5.78 × 10^{10}, while the constrained mean is reduced to roughly 4.95 × 10^{10} (Fig. 8c). These lower increases in SSP5-8.5 compared to SSP2-4.5 are due primarily to weaker overall population growth in SSP5-8.5 in Africa[93], rather than a negative change in rural exposure, which is also reflected in the population effect in urban exposure (Fig. 8a-c and Supplementary Figs. S24-S26a-c).

In contrast to population exposure, which is generally higher under SSP2-4.5, projected GDP exposure to Rx1day (Fig. 8d) is markedly greater under SSP5-8.5 across all African subregions, with model constraints similarly moderating but not reversing the direction of change. Under SSP2-4.5, Africa-wide GDP exposure reaches ~2.26 × 10^{15} USD mm day^{-1} in the unconstrained EnsMean and is reduced to ~2.45 × 10^{15} USD mm day^{-1} when constraints are applied. The amplification under SSP5-8.5 is much larger: unconstrained exposure climbs to ~5.81 × 10^{15} USD mm day^{-1} and is reduced to ~5.24 × 10^{15} USD mm day^{-1} with the constrained EnsMean. WAF dominates the continental signal, with unconstrained SSP5-8.5 exposure of ~3.18 × 10^{15} USD mm day^{-1} compared to ~2.82 × 10^{15} under constraints, while CAF, NEAF, and SEAF (MED, SAH, WSAF, and MDG) exhibit moderate (or minimal) but consistent increases. Decomposition of GDP exposure confirms that all three effects contribute positively across subregions, except for the climate and interaction effects in MED (Supplementary Figs. S24-S26d). These result in a minor negative contribution that partially offsets the net positive increase in GDP exposure from the GDP effect in MED. Overall, these patterns reflect the underlying socioeconomic assumptions of the two scenarios. SSP2-4.5 represents moderate population growth and steady economic development, whereas SSP5-8.5 assumes similar but slightly weaker population growth, with high migration and urbanization leading to rapid GDP expansion[93,94]. Consequently, higher total populations under SSP2-4.5 contribute to higher total population exposure, while accelerated economic growth under SSP5-8.5 may increase GDP exposure in densely populated or rapidly urbanizing regions.” **(lines 529-587)**

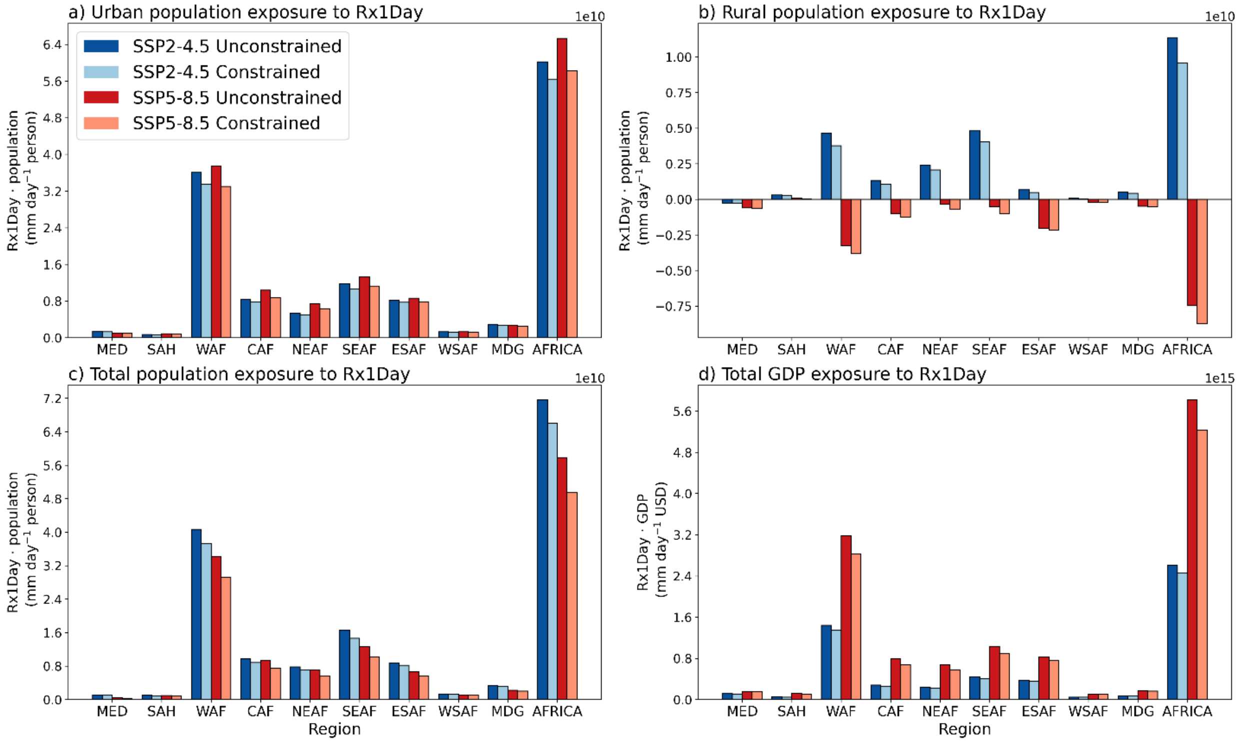

Figure 8: Population and economic exposure to extreme precipitation. Bar plots show the change in total exposure to unconstrained and constrained Rx1day for the (a) urban population, (b) rural population, (c) total population, and (d) gross domestic product (GDP) across Africa and its subregions under the SSP2-4.5 and SSP5-8.5 scenarios. Change is calculated for the future period (2070–2099) compared to the historical period (1985–2014).

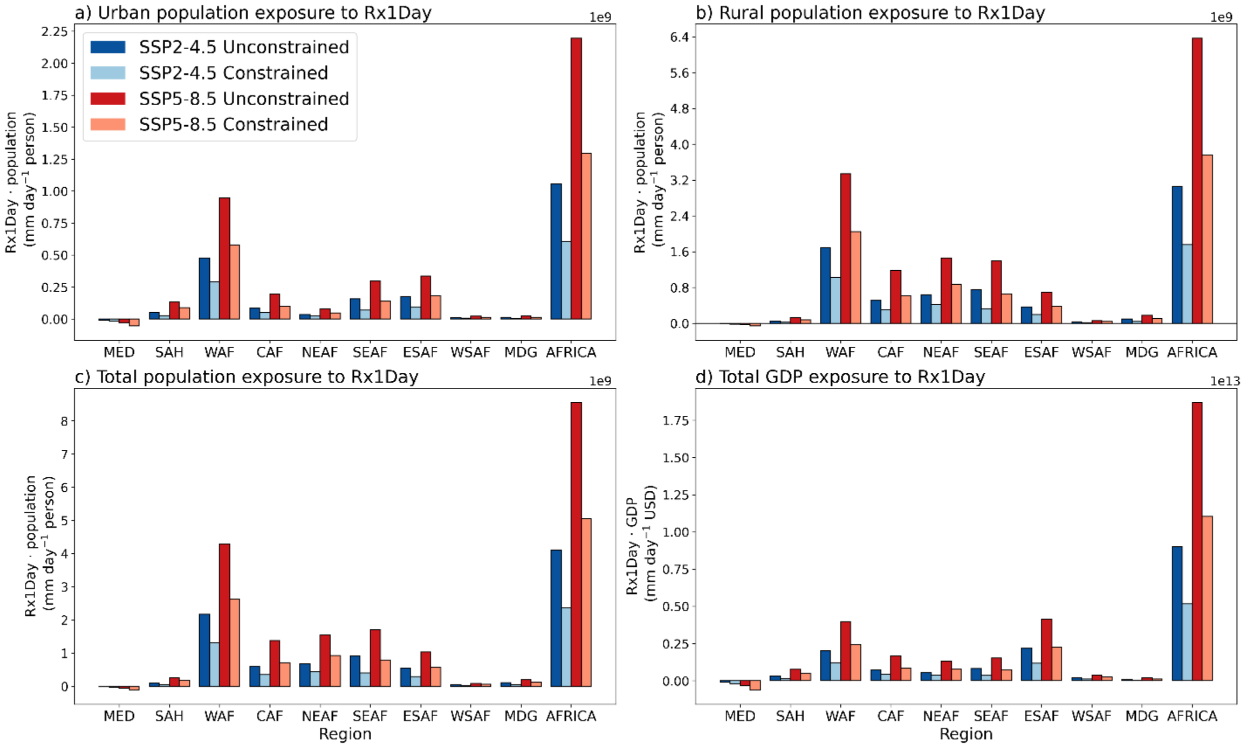

Figure S24. Bar plots show the change in the climate effect (Rx1day change) component of total exposure to unconstrained and constrained Rx1day for the (a) urban population, (b) rural population, (c) total population, and (d) gross domestic product (GDP) across Africa and its subregions under the SSP2-4.5 and SSP5-8.5 scenarios. Change is calculated for the future period (2070–2099) compared to the historical period (1985–2014).

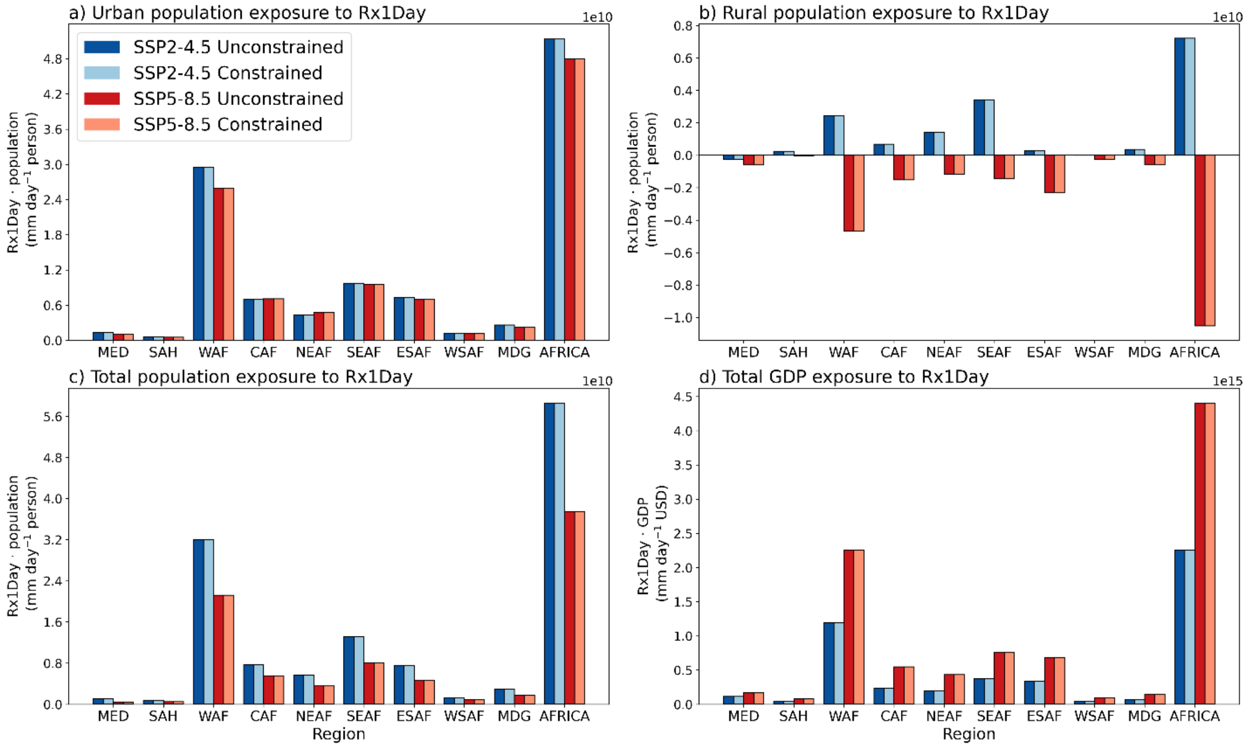

Figure S25. Bar plots show the change in the population/GDP change component of total exposure to unconstrained and constrained Rx1day for the (a) urban population, (b) rural population, (c) total population, and (d) gross domestic product (GDP) across Africa and its subregions under the SSP2-4.5 and SSP5-8.5 scenarios. Change is calculated for the future period (2070–2099) compared to the historical period (1985–2014).

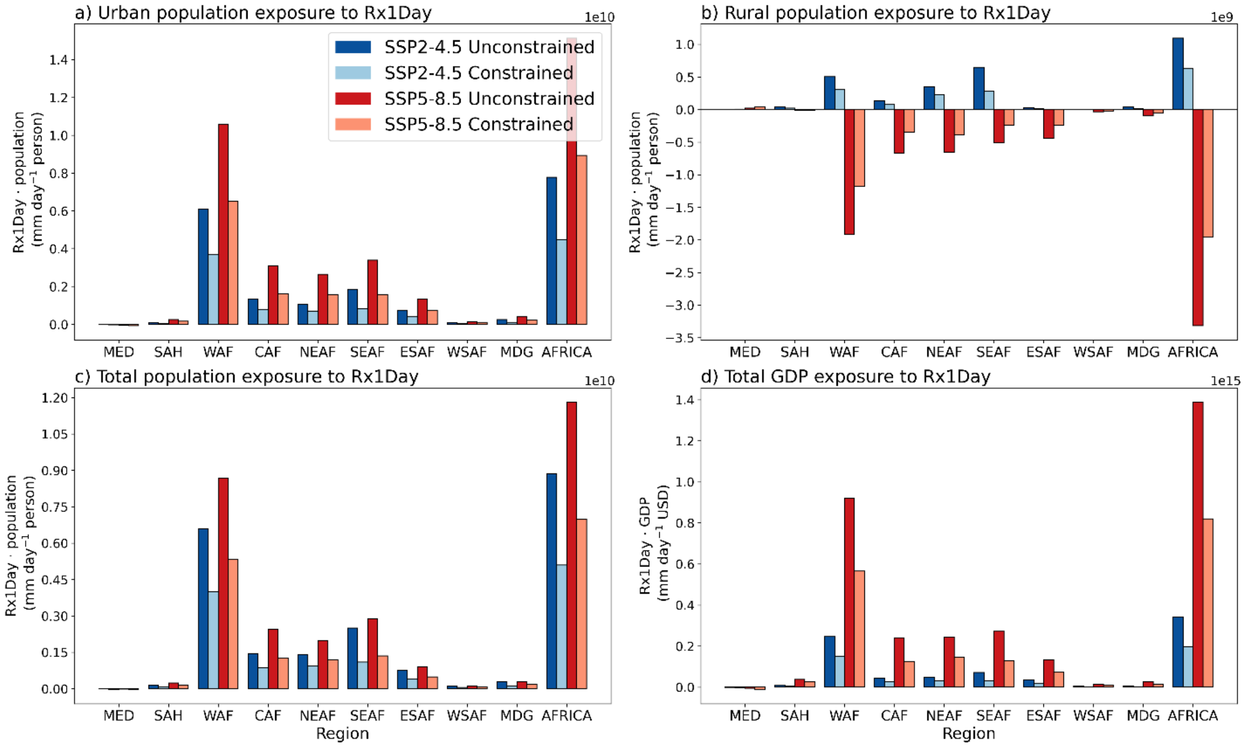

Figure S26. Bar plots show the change in the interaction component of total exposure to unconstrained and constrained Rx1day for the (a) urban population, (b) rural population, (c) total population, and (d) gross domestic product (GDP) across Africa and its subregions under the SSP2-4.5 and SSP5-8.5 scenarios. Change is calculated for the future period (2070–2099) compared to the historical period (1985–2014).

Comment: *Line 719. Here do not provide satisfactory evidence to neglect eddy effects. Need to show this term over Africa as a minimum and explore within the uncertainty decomposition.* **Response: We thank the reviewer for raising this point. Figure S9 presents additional analysis confirming that the residual component of Rx1day_scaling has a minimal contribution. Given this, we chose not to include it in subsequent analyses.**

Comment: *Line 783 and line 788, need to explain the adjustment factors and the forms these take. What are the underlying assumptions and caveats associated with this?*

Response: Thank you for this comment. The method section was revised accordingly:

"The adjustment factor $\frac{\rho\sigma_z\sigma_x}{\sigma_x^2+\sigma_y^2}$ determines how strongly observational information modifies the unconstrained model mean. This factor increases when the intermodel correlation between the predictor and predictand is strong and when observational uncertainty is small relative to the spread of model simulations. Conversely, when observational uncertainty is large or the intermodel relationship is weak, the adjustment factor becomes small, and the constrained projection approaches the original multimodel mean. "   (lines 965-970)

Comment: *Line 796. Need to briefly explain the framework and calculations performed to do the decomposition here.*

Response: We thank the reviewer for this suggestion. In the revised manuscript, we have expanded the Methods section to summarize the key steps of the Hawkins and Sutton framework and clarify how the total variance in projected Rx1day is decomposed into contributions from internal variability, model uncertainty, and scenario uncertainty. We decided not to include step-by-step mathematical equations because the manuscript is already becoming lengthy. Instead, we direct readers to our previous work, where these equations are presented in detail.

"We quantify these uncertainties following the framework of Hawkins and Sutton (2009, 2011)[137,138], and the procedure outlined in Akinsanola et al. (2024; see their Supporting Information, Eqs. 1–6)[5]. For each model and scenario, the time series of Rx1day is fitted with a smooth fourth-order polynomial, and deviations from this fit are taken as residuals. These residuals are attributed to natural internal variability arising from intrinsic fluctuations in the climate system. Internal variability is estimated from the multimodel mean of the variance of residuals across scenarios and time. Model uncertainty is defined as the multiscenario mean of the variance of the smooth fit across models. Scenario uncertainty is defined as the variance of the multimodel and multiscenario mean smooth fit. Assuming model uncertainty, scenario uncertainty, and internal variability are independent of each other, the total uncertainty can be expressed as the sum of these three components. The relative contribution of each component is computed as its fraction of the total variance in projected Rx1day changes. This decomposition provides a quantitative framework for assessing how the importance of different sources of uncertainty evolves throughout the 21st century." (lines 992-1005)

Thank you very much for your helpful suggestions, which have helped us improve the quality of the manuscript.

Reviewer #2

Summary and recommendation

The study examines projected changes in extreme precipitation over the African continent. Specifically, the authors quantify their future changes in CMIP6 projections, identify the underlying mechanisms of these changes, constrain uncertainties arising from inter-model differences, and assesses implications on exposure of population and GDP. The study finds that future increases in precipitation extremes are largest in Equatorial Africa where dynamic effects from diabatic heating in upward motion further amplify the spatially homogeneous thermodynamic influence. Extremely rare extremes are expected to occur more often towards the end of the 21st century. The study also uses a statistical method to reduce uncertainties arising from different warming trends across models, still resulting in an increase in extreme precipitation throughout much of the continent. Finally, the authors show that exposure of populations and assets will substantially increase as a result of extreme precipitation increases and population growth, specifically in urban areas, implying increased risk from hydrological hazards in the future.

Overall, I found the study very thorough, relevant, insightful, clearly structured, and well written. The manuscript covers a broad variety of aspects, from projected changes in extreme precipitation, to their thermodynamic and dynamic drivers, uncertainties stemming from inter-model differences, scenarios, and internal variability, to reducing these uncertainties using a statistical framework, and to quantifying the changes in exposure of population and assets. In my opinion, the manuscript is already in excellent shape. Below I list a few general and more specific comments which the authors may consider for improving the manuscript. I recommend publication of the manuscript after considering these suggestions.

Response: We sincerely thank the reviewer for the positive and encouraging assessment of our manuscript. We appreciate the recognition of the study's scope, structure, and relevance, as well as the constructive suggestions provided to further improve the paper. In the revised manuscript, we have carefully considered all comments and implemented the recommended clarifications and revisions to strengthen the presentation and interpretation of our results.

General comments

Comment: *Expression of projected precipitation changes. Already from the abstract it is clear that the study expresses the extreme precipitation changes in actual amounts. Have the authors considered expressing these in relative terms? I wonder whether this would give a more representative picture of extreme precipitation changes. When using actual values, these are almost automatically largest where the actual precipitation values are largest (e.g., Equatorial Africa). By contrast, relative extreme precipitation changes could be very large in regions with little precipitation too. Would adding a few figures in the supplement, similar as in the manuscript, but then expressing the changes in relative terms, be of added value to the study? This comment refers mostly to the first results section "Unconstrained projected precipitation extremes".*

<u>Response</u>: We thank the reviewer for this suggestion. We have added the relative change in Rx1day, defined as the ratio of future Rx1day to the historical ensemble-mean Rx1day (i.e., future/historical), for both SSP scenarios and for constrained versus unconstrained projections, to illustrate the magnitude of future changes. Across Africa, Rx1day increases by 15% (32%) under SSP2-4.5 (SSP5-8.5) in the unconstrained projections, with NEAF increasing by up to 25% (56%); applying the constraint reduces the mean increase to 9% (19%), with NEAF reduced to 16% (34%). Only MED shows a decrease, of about -1% (-3%) unconstrained to -2% (-7%) constrained under SSP2-4.5 (SSP5-8.5). We have included the corresponding figure and highlighted these changes in the manuscript.

"we examine the relative change in Rx1day, defined as the ratio of future to historical Rx1day values. This metric highlights pronounced regional contrasts in projected Rx1day across Africa (Fig. S5). NEAF shows the strongest intensification, with ratios of 1.25 and 1.56 under SSP2-4.5 and SSP5-8.5, respectively, compared to Africa-wide increases of 1.15 and 1.32, respectively. The northern and central Africa subregions (SAH, WAF, CAF, NEAF) also exceed a ratio of 1.4 under SSP5-8.5, whereas MED is the only exception, exhibiting a slight projected decrease (~1–3%)." (lines 203-209)

"Notably, this brings the area-averaged projected relative increase in Rx1day from above 40% in unconstrained projections (as high as 56% in NEAF) to below 35% across all subregions, thereby reducing the most extreme projected signals (Supplementary Fig. S5)." (lines 471-473)

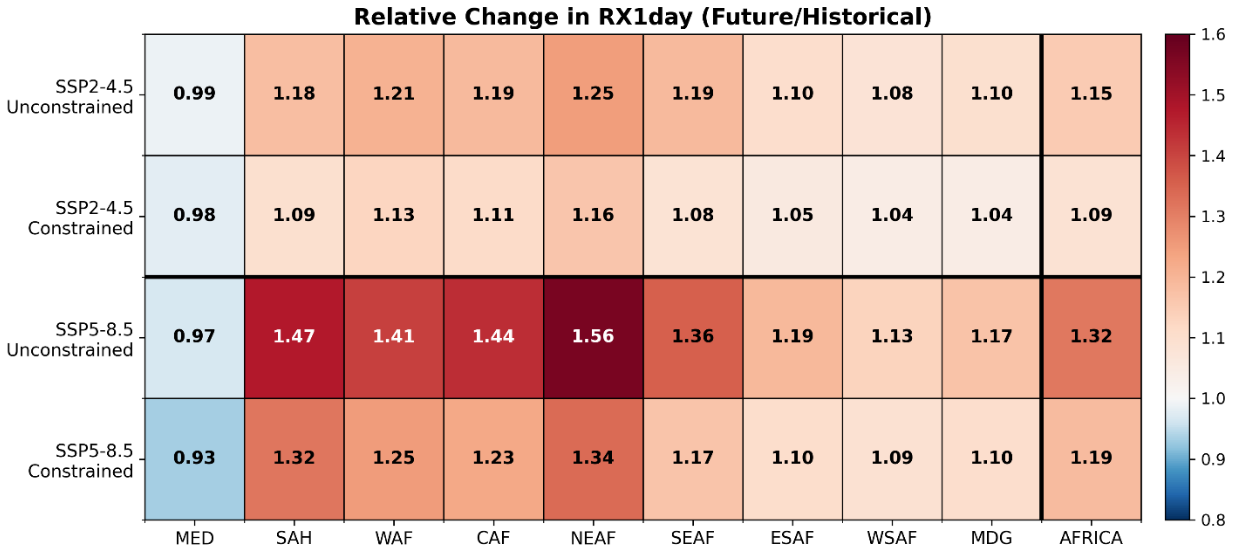

Figure S5. The relative change in Rx1day, expressed as the ratio of the weighted area-averaged future Rx1day (2070-2099) to the historical period (1985-2014), for the SSP2-4.5 and SSP5-8.5 scenarios across Africa and its subregions.

Comment: *Study region (e.g., Figure 1). I realized that the northern Africa, the zone northward of the Sahara domain, is not included in the study. I suppose because this region falls under the Mediterranean region according to the IPCC definition. Nevertheless, I have the feeling that*

including this northern part of Africa would be rather important for the study, given its central focus on the African continent, especially given the large number of people living in this region, e.g., Morocco, Algeria, Tunisia, and Libya. Have the authors considered including this region in their study? Even if it would deviate from the commonly adopted IPCC definition, I feel that covering the part of northern Africa could make the study more complete.

Response: Thank you for the suggestion. We agree that excluding the Mediterranean (MED) region omitted parts of northern Africa that should be included. Therefore, following your suggestion, we have added the MED region that overlaps with the African continent in this study. This change is reflected in the updated Methods section (copied below) and in all area-averaged Figures.

"For regional analyses, we focus on nine subregions based on the IPCC AR6 WGI regional classification[102]. These include eight African subregions: Sahara (SAH), Western Africa (WAF), Central Africa (CAF), North Eastern Africa (NEAF), South Eastern Africa (SEAF), East Southern Africa (ESAF), West Southern Africa (WSAF), and Madagascar (MDG). In addition, we include the Mediterranean (MED) region from the IPCC AR6 classification; however, only the portion of MED that overlaps with Africa is considered in this analysis." (lines 762-768)

Specific comments

Comment: *Lines 61-74. The text summarizes here the atmospheric processes and variability patterns linked to extreme precipitation in different parts of Africa. However, it seems that northern Africa, including the Sahara and Mediterranean coast are missed, where extratropical forcing in the form of Rossby wave dynamics and surface (extratropical) cyclones are central to the occurrence of extreme precipitation. Adding a sentence on this may strengthen the manuscript.*

Response: Thank you for this helpful suggestion. We agree that northern Africa, including the Saharan and Mediterranean coastal regions, was not explicitly discussed in the original text despite the importance of extratropical dynamics in driving extreme precipitation there. To address this, we have added a sentence describing the role of Rossby wave dynamics and extratropical cyclones in this region.

"For instance, in northern Africa, including the Saharan and Mediterranean coast, extreme precipitation events are often associated with extratropical dynamics, including upper-tropospheric Rossby wave breaking and surface extratropical cyclones that drive anomalous moisture transport into an otherwise hyper-arid region[36-39]." (lines 63-67)

Comment: *Figure 1. The central panel of the figure shows a geographical map of Africa with topography. I wonder whether it would be more informative if the map would show characteristics of extreme precipitation, for example, the multi-model-mean extreme precipitation value of Rx1day or the multi-model mean change in extreme precipitation. This would give more insights in these characteristics or changes at the grid scale.*

Response: We thank the reviewer for this helpful suggestion. The multi-model mean Rx1day for the historical period, together with observations, is already presented in the Supplementary Material, and the projected spatial changes in extreme precipitation are

shown in Fig. 2. Since these characteristics and changes are already illustrated elsewhere in the manuscript, we prefer to retain the topography in Fig. 1. We believe this provides a clearer geographical context and helps readers who may be less familiar with the regional heterogeneity across Africa.

Comment: *Line 201. Remove "better"?*

<u>Response</u>: Thank you for pointing this out. We have removed "better" from the revised manuscript.

Comment: *Figure 2. Due to the used color bar in panels (a-d), as well as in several other figures, it is not easy to distinguish whether extreme precipitation increases or decreases if changes are relatively low as a result of the yellowish colors near zero values. Using a diverging color bar, perhaps with white color assigned to values near zero, may make the figures clearer.*

<u>Response</u>: We thank the reviewer for this helpful suggestion. We have revised the colormap in Fig. 2 and the other relevant figures by using a diverging color scale, with values near zero shown in a contrasting tone, to make increases and decreases in extreme precipitation easier to distinguish. This change improves the overall clarity and readability of the figures.

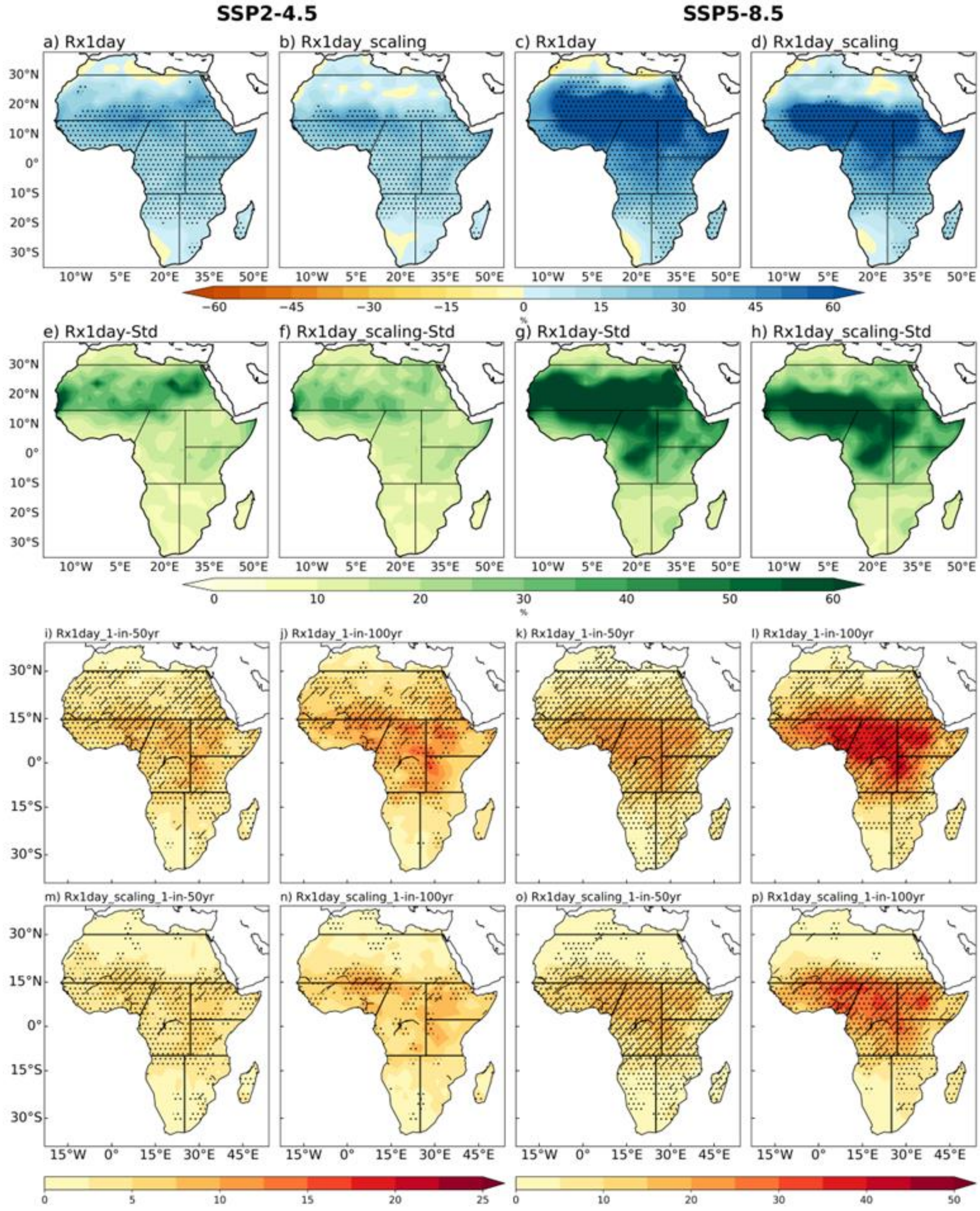

SSP2-4.5
SSP5-8.5
a) Rx1day
b) Rx1day_scaling
c) Rx1day
d) Rx1day_scaling
e) Rx1day-Std
f) Rx1day_scaling-Std
g) Rx1day-Std
h) Rx1day_scaling-Std
i) Rx1day_1-in-50yr
j) Rx1day_1-in-100yr
k) Rx1day_1-in-50yr
l) Rx1day_1-in-100yr
m) Rx1day_scaling_1-in-50yr
n) Rx1day_scaling_1-in-100yr
o) Rx1day_scaling_1-in-50yr
p) Rx1day_scaling_1-in-100yr
%

Figure 2: Projected changes in precipitation extremes and their scaling across Africa. (a–d) EnsMean changes in annual 1-day maximum precipitation (Rx1day) and precipitation-extreme scaling (%) under the SSP2-4.5 and SSP5-8.5 scenarios. Stippling denotes regions where at least 70% of models agree on the sign of the EnsMean change. (e–h) Multi-model standard deviation in Rx1day and Rx1day_scaling (%) under the SSP2-4.5 and SSP5-8.5 scenarios. (i–l) Probability ratios of (i,k,m,o) Rx1day and (j,l,n,p) Rx1day scaling frequency relative to the historical baseline period (1985–2014), estimated using the Gumbel extreme value distribution for (i-l) 1-in-50-year and (m-p) 1-in-100-year return periods under (i,j,m,n) SSP2-4.5 and (k,l,o,p) SSP5-8.5. Stippling and hatching in (i–p) denote grid points where at least 70% of models agree on the direction of change (increase/decrease) with EnsMean and where the change is statistically significant based on bootstrapping, respectively.

Comment: *Figure 2i,j. Could it be of added value to indicate statistical significance for changes in return periods in the different regions, for example, by adding hatching to the squares? Or are all changes in probability ratios significant anyways?*

<u>Response</u>: We thank the reviewer for this helpful suggestion. To better illustrate the spatial structure and robustness of the results, we have updated the figure by adding a spatial map in the main text, including robustness information indicated by stippling and hatching. The stippling denote grid point where at 70% models agree on the ensemble mean sign of the change and hatching denote statistical significance estimated using a bootstrap method. The bootstrap analysis was performed by resampling the historical distribution, and significance was assessed using the 95% confidence interval. The corresponding analysis technique is described in the Methods section. For this reason, the original area-averaged panels have been moved to the Supplementary Material, while the spatial figure is now presented in the main text (see Figure 2 above).

Comment: *Lines 235-239 and Figure 2i,j. It took me a while to realize where in the figure I could find the numbers mentioned in the text. Would it be easier for a reader if the right-most column, corresponding to Africa, is emphasized by drawing a vertical line between this and the neighboring column to the left?*

<u>Response</u>: Thank you for your comment. We have added thick lines to separate the SSP scenario and the Africa column for better readability, as seen in the figure below.

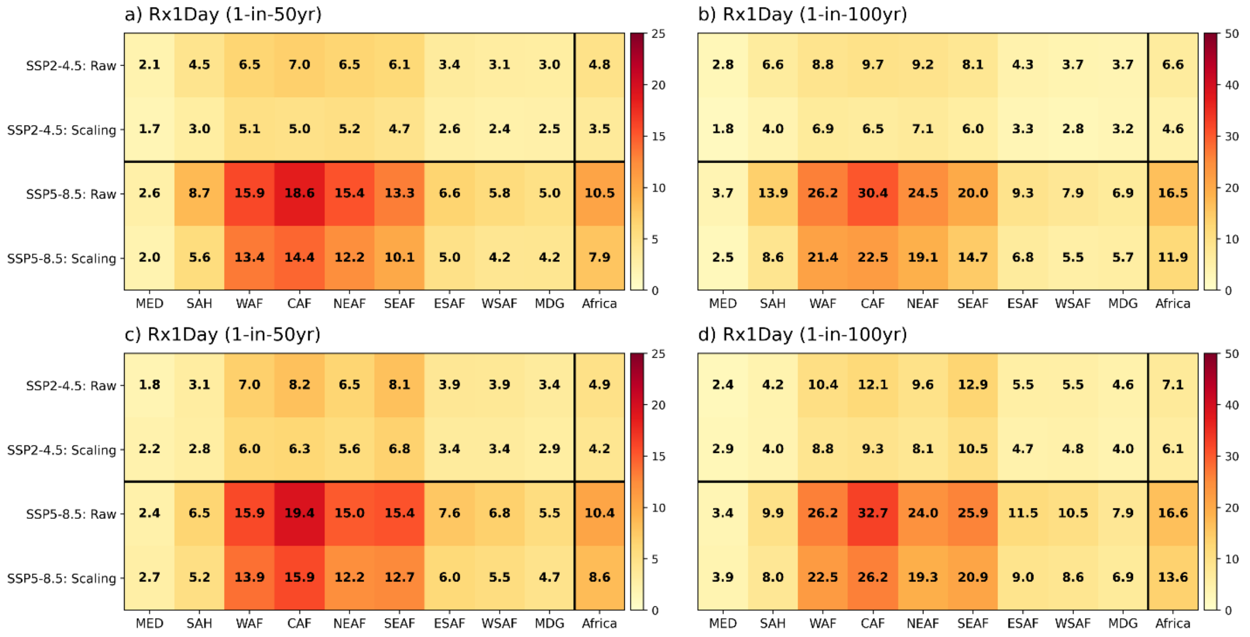

Figure S7. Area-weighted probability ratios for the occurrence of (a, c) 1-in-50-year and (b, d) 1-in-100-year future-to-historical Rx1day events, estimated using a (a, b) Gumbel extreme value and (c, d) GEV distribution, under the SSP2-4.5 and SSP5-8.5 scenarios. Results are presented for both the raw Rx1day projections and Rx1day_scaling, covering all of Africa and its individual subregions under both SSP2-4.5 and SSP5-8.5.

Comment: *Line 276. Is it correct to attribute the decreased influence from the dynamics to "enhanced subsidence"? Wouldn't it be more accurate to say that vertical motion / ascent is weaker during extreme precipitation?*

<u>Response</u>: We thank the reviewer for this helpful clarification. We agree that describing the signal as weaker ascent during extreme events is more precise than attributing it to "enhanced subsidence," since the scaling diagnostic reflects changes in vertical velocity during precipitation extremes rather than a transition to subsiding conditions. We have therefore revised the text to clarify that the dynamical contribution reflects a "weaker upward motion" (lines 306-307).

Comment: *Line 280-281. On what is the phrase "projected meridional contraction of the winter storm track" based? Isn't it rather a weakening or poleward shift of the storm track that is relevant?*

<u>Response</u>: The phrase "projected meridional contraction of the winter storm track" is based on prior studies (refs 73-75) documenting narrowing alongside poleward shifts under warming. This manifests as a northward shift in midlatitude troughs (Fig. 2; following Chang et al. 2012 ref 75), reducing activity at the northern and southern edges of the North Atlantic storm track parts while activity increases poleward of 60°N, indicating relevant contraction, consistent with weakened cyclone frequency.

Chang, E. K. M., Y.Guo, and X.Xia (2012), CMIP5 multimodel ensemble projection of storm track change under global warming, J. Geophys. Res., 117, D23118, doi:10.1029/2012JD018578.

Comment: *Line 281. Why is reference 36 included, which seems to relate to African easterly waves and not to winter storm track and cyclones?*

Response: Although the referenced study primarily focuses on African easterly waves (AEWs), it was originally cited because it discusses the migration of low-level moisture convergence over the Sahel and Guinea Coast and its influence on AEW activity. However, we agree that this reference may be confusing in the context of winter storm tracks and cyclones. Therefore, we have removed this citation from the revised manuscript.

Comment: *Lines 315-316. I couldn't exactly follow the phrase "and somewhat consistent but less under SSP2-4.5".*

Response: We thank the reviewer for pointing this out. We agree that the phrase was unclear. We have revised the sentence to clarify that SSP2-4.5 shows a similar spatial pattern of warming, but with smaller magnitudes than SSP5-8.5.

"The strongest warming is projected over MED and SAH, exceeding 5°C, while in most other subregions warming is well below 5°C under SSP5-8.5, with a similar but weaker pattern under SSP2-4.5." (lines 346-348)

Comment: *Lines 327-329. Perhaps clarify that it is not the increase in 500-hPa geopotential height values itself, but rather a change in gradient that matters when speaking about a strengthened anticyclonic circulation. (spatially homogeneous increases in Z500 may be due to thermal expansion of the atmosphere and do not necessarily imply a strengthening of anticyclones). Showing the actual 500-hPa geopotential height contours under present-day climate conditions overlaid on future changes may help providing a reference of the actual circulation.*

Response: We thank the reviewer for this key point. The revised text now clarifies that anticyclone strengthening arises from Z500 gradients and not background thermal expansion. We have updated the figure to show the historical 500-hPa geopotential height contours on the original figure. The updated text and figure are copied below

"strengthened subtropical anticyclonic circulation over Africa is reflected in steeper 500 hPa geopotential height gradients relative to the historical period (Supplementary Fig. S15e–f). These enhanced gradients promote subsidence and more persistent warm conditions, with a stronger response poleward of 15°N and 15°S. These changes further enhance atmospheric moisture availability (Supplementary Fig. S15a–b), thereby favoring more extreme precipitation events." (lines 360-365)

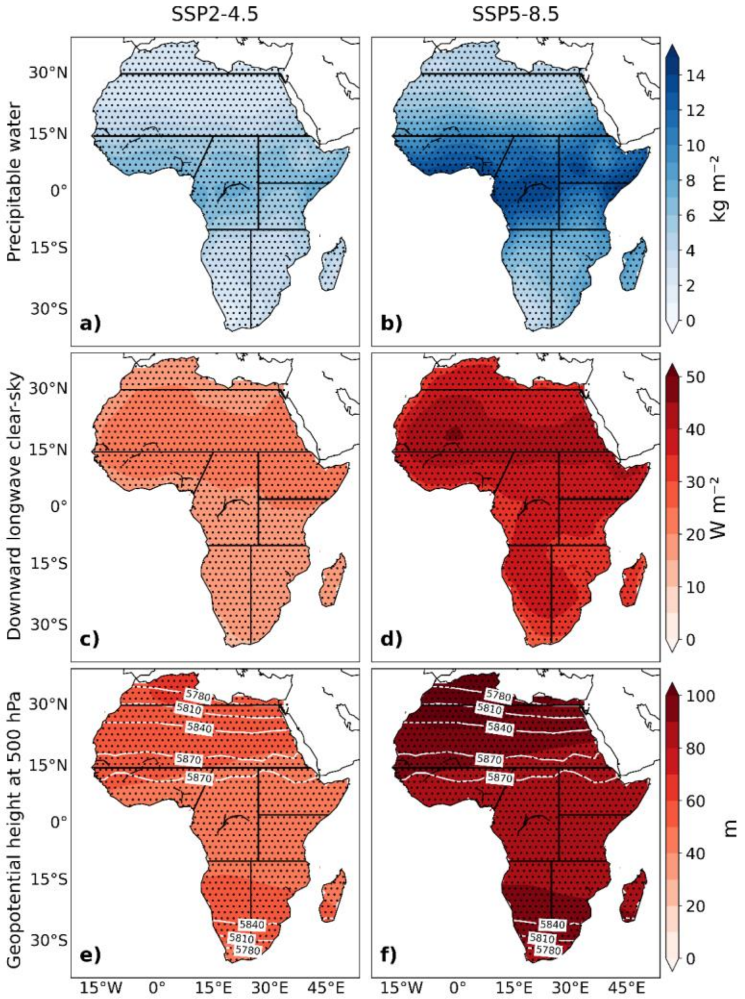

Figure S15. EnsMean changes in (a, b) precipitable water, (c, d) clear-sky downward longwave radiation, and (e, f) 500-hPa geopotential height for the late twenty-first century (2070–2099) relative to the historical baseline period (1985–2014) under the SSP2-4.5 and SSP5-8.5 scenarios. Stippling indicates regions where at least 70% of models agree on the sign of the EnsMean change. White contours in (e, f) indicate the climatological mean 500-hPa geopotential height during the historical baseline period (1985–2014).

Comment: *Lines 337-338. It is not clear to me where the inference "…are associated with weakened (enhanced) large-scale ascent and vertical moisture convergence" comes from.*

Response: We thank the reviewer for pointing out that this inference was not sufficiently explained. The interpretation was based on the diagnostics of the vertical velocity term (ω_e) used in the scaling framework, which represents changes in large-scale vertical motion during extreme precipitation events. To avoid implying a separate moisture-convergence analysis that was not explicitly presented, we have revised the sentence to directly refer to the diagnosed changes in vertical velocity shown in Fig. 3j,l.

“Decreased (increased) DY contributions are evident over WSAF as well as northern SAH and MED (CAF, WAF, NEAF, and SEAF; Fig. 3b,d) and are associated with weakened (enhanced) vertical velocity (Fig. 3j,l).” (lines 369-371)

Comment: *Lines 349-351. In fact, Fig. 5ab,f, seem to show that contributions from the horizontal temperature advection term are nearly of similar magnitude as diabatic heating over northern Africa and western South Africa. This seems relevant as it suggests a weakening influence of midlatitude storm track, consistent with existing literature.*

Response: We thank the reviewer for this insightful observation. We agree that horizontal temperature advection contributes comparably to diabatic heating over northern Africa and western southern Africa. This contribution likely reflects changes in large-scale circulation linked to midlatitude storm-track dynamics. Previous studies have shown that climate warming can lead to shifts in the position and intensity of midlatitude storm tracks, which strongly influence moisture transport and precipitation patterns in adjacent subtropical regions (e.g., Paul A. O’Gorman, 2010; Yutian Wu et al., 2010). Such circulation changes can weaken baroclinic activity and reduce synoptic-scale ascent and moisture transport toward subtropical land regions, contributing to reduced precipitation extremes. We have revised the manuscript to explicitly acknowledge the role of horizontal temperature advection and its potential link to storm-track changes.

“In contrast, over subtropical regions such as WSAF and SAH, contributions from horizontal temperature advection are somewhat comparable in magnitude to diabatic heating (Fig. 5b,d,f,h). These signals are consistent with projected shifts and modifications of midlatitude storm tracks that alter baroclinic eddy activity and moisture transport into subtropical regions[79,80], contributing to reduced upward motion and limiting convective processes (and thus leading to reduced precipitation extremes) despite strong surface warming.” (lines 381-387)

References:

O’Gorman, P. A. (2010). *Understanding the varied response of the extratropical storm tracks to climate change.* Proceedings of the National Academy of Sciences. https://doi.org/10.1073/pnas.1011547107

Wu, Y., Ting, M., Seager, R., Huang, H.-P., & Cane, M. (2010). *Changes in storm tracks and energy transports in a warmer climate.* Climate Dynamics. https://doi.org/10.1007/s00382-010-0776-4

Comment: *Lines 359-365. To me, Fig. 6 seems to tell that internal variability decreases with lead time whereas scenario uncertainty increases, but most of all, that model uncertainty dominates. The claim that model uncertainty becomes the dominant contributor by the end of the 21st century seems not to match Fig. 6, showing that this term actually decreases in some regions over the 2nd half of the 21st century, but dominates the overall uncertainty, independent of changes with lead time.*

Response: We thank the reviewer for this careful reading of Fig. 6 and for pointing out the ambiguity in our wording. We have revised the text to clarify this interpretation and to more accurately reflect the information shown in Fig. 6.

“Our results show that, across all regions, uncertainty from internal variability (green shading) decreases with lead time, whereas scenario uncertainty (orange shading) generally emerges around mid-century (2030–2040) and increases toward the end of the century. Model uncertainty (blue shading) contributes the most to total variance throughout the projection period, although its fractional contribution slightly decreases in the late 21st century in some regions as scenario uncertainty grows. Overall, model uncertainty dominates the projection uncertainty, accounting for more than 70% of the total variance in Rx1day projections across most African regions and reaching up to about 85% over MDG.” (lines 395-402)

Comment: *Lines 371-372. And what about internal variability? Is this source of uncertainty also associated with DY processes?*

Response: We thank the reviewer for raising this important point. Internal variability can indeed be driven by both thermodynamic and dynamical processes; however, in the context of precipitation extremes it is often closely linked to fluctuations in large-scale circulation and vertical motion, which affect the dynamical component of extreme precipitation. We have revised the text to acknowledge that internal variability can also be associated with dynamical processes, particularly through natural variability in atmospheric circulation patterns that modulate extreme precipitation events.

“internal variability likely reflects fluctuations in large-scale atmospheric circulation and vertical motion that modulate the DY component of extreme precipitation” (lines 408-409)

Comment: *Line 373-375. What about misrepresentations of regional circulation patterns as a source of uncertainty?*

Response: We thank the reviewer for highlighting this important point. In addition to subgrid-scale physical parameterizations, uncertainties in the representation of large-scale and regional circulation patterns can also contribute to differences in the dynamical (DY) component of precipitation extremes across models. We have revised the manuscript to explicitly acknowledge this additional source of model uncertainty.

“as well as differences in how models represent regional atmospheric circulation patterns that regulate moisture transport and vertical motion[81,82]” (lines 413-415)

Comment: *Line 433. “consistently indicate larger increase in Rx1day”, larger, than what?*

Response: We thank the reviewer for pointing out that the comparison was not clearly specified. Our intention was to indicate that both unconstrained and constrained projections show larger increases in Rx1day relative to the historical baseline period. We have revised the sentence to clarify the reference point and improve the wording.

“Accordingly, both unconstrained and constrained projections consistently indicate substantial increases in Rx1day relative to the historical period” (lines 488-489)

Comment: *Line 467-468. Perhaps add a reference to the method section? At first, it wasn't clear to me where the data on population exposure and GDP comes from.*

Response: We thank the reviewer for pointing this out. We have now clarified the data sources in the text and added a reference to the Methods section. The population data used in the analysis are sourced from the NASA Socioeconomic Data and Applications Center (SEDAC), which provides a baseline for the year 2000 along with decadal projections following SSP pathways. The GDP data are obtained from Murakami et al. (2021), which provide projections at a similar temporal resolution. These details are now explicitly described in the Methods section.

"The NASA Socioeconomic Data and Applications Center (SEDAC) provides projected gridded population data following SSP pathways at 1/8° resolution, with the year 2000 as the baseline[111]. Similarly, Murakami et al. (2021)[112] provide the projected GDP across SSP scenarios at 1/12° resolution." (lines 785-788)

Comment: *Lines 512-513. The sentence seems to contrast fig. 8a,c showing that a greater number of people is exposed under SSP5-8.5 than under SSP2-4.5, at least, for urban and total population.*

Response: We thank the reviewer for pointing this out. The sentence was intended to refer to total population exposure, which was not clearly stated in the previous version and could therefore appear inconsistent with Fig. 8a and 8c. We have revised the text to explicitly clarify that the statement refers to total population exposure, which resolves the apparent contradiction.

"Consequently, higher total populations under SSP2-4.5 contribute to higher total population exposure, while accelerated economic growth under SSP5-8.5 may increase GDP exposure in densely populated or rapidly urbanizing regions." (lines 584-587)

In addition, this section has undergone substantial revision following comments from Reviewer 1. In the previous version, the analysis underrepresented total exposure because it did not fully account for the independent contributions of changes in climate (ΔRx1day) and population/GDP to exposure. We have now corrected the formulation and decomposed exposure into the climate effect, the social effect (population/GDP change), and the interaction effect. This updated framework provides a more complete representation of total exposure across scenarios. The methods, results/discussion, and corresponding figures have been updated accordingly, and the revised text and figure are provided below.

"Exposure of population and GDP: The NASA Socioeconomic Data and Applications Center (SEDAC) provides projected gridded population data following SSP pathways at 1/8° resolution, with the year 2000 as the baseline[111]. Similarly, Murakami et al. (2021)[112] provide the projected GDP across SSP scenarios at 1/12° resolution. Using these datasets, we evaluate changes in population and GDP exposure for each grid cell and the relative importance of different factors in driving these changes[113,114]. These factors include the climate, population/GDP, and interaction effects.

$$\Delta Exposure_{POP-Rx1day} = \overline{Pop} \times \Delta Rx1day + \Delta Pop \times \overline{Rx1day} + \Delta Pop \times \Delta Rx1day \quad (1)$$

$$\Delta Exposure_{GDP-Rx1day} = \overline{GDP} \times \Delta Rx1day + \Delta GDP \times \overline{Rx1day} + \Delta GDP \times \Delta Rx1day \quad (2)$$

Here, Δ represents the mean change between the future period (2070–2099) and the historical period (1985–2014), and the overbar represents the mean during the historical period. For the population/GDP, the year 2000 was chosen as the historical period rather than a multiyear mean because socioeconomic data are available at decadal intervals, and the 2010 data are projections under the SSP scenarios rather than observations. The climate effect is defined as the impact of the Rx1day change on the historical population/GDP (1st term in the equation). The population/GDP effect is the impact of changes in population/GDP relative to historical Rx1day (2nd term). The interaction effect accounts for changes in both population/GDP and Rx1day (3rd term).**" (lines 785-801)**

"Our results show that model constraints consistently moderate the projected impacts. Decomposition of total exposure into climate, population/GDP, and interaction effects reveals that population/GDP changes are the dominant driver, followed by the interaction effect, with the climate effect being the smallest contributor, an order of magnitude lower than the others (Supplementary Figs. S24-26). The climate effect contributes positively across all regions, except for a minor negative contribution in the MED region, consistent with the decrease in Rx1day also noted in the relative change analysis (Supplementary Fig. S24). Urban populations experience substantial increases in exposure under both SSP2-4.5 and SSP5-8.5, regardless of whether projections are constrained or unconstrained. WAF exhibits the largest urban exposure, increasing from approximately 3.61×10^{10} person mm day^{-1} under SSP2-4.5 to nearly 3.75×10^{10} under SSP5-8.5 in the unconstrained projections (Fig 8a). The constrained EnsMean shows lower increases, around 3.35×10^{10} and 3.30×10^{10} person mm day^{-1}, respectively. CAF, NEAF, SEAF, and ESAF also show moderate increases, from about 0.5×10^{10} to nearly 1.1×10^{10} person mm day^{-1}. Increases in MED, SAH, WSAF, and MDG are relatively smaller but consistent with their population sizes. These upward trends indicate that both Rx1day intensification and demographic growth, reflected in the large population and interaction effects, contribute to increasing urban exposure across all subregions (Fig. 8a, and Supplementary Figs. S25a, S26a).

On the other hand, rural population exposure shows modest increases under SSP2-4.5 but declines under SSP5-8.5. WAF exhibits the largest negative rural exposure, approximately -0.33×10^{10} person mm day^{-1} in the unconstrained SSP5-8.5 projections and -0.38×10^{10} under constraints (Fig. 8b). CAF, NEAF, SEAF, and ESAF also show modest negative rural exposure up to -0.2×10^{10} in the unconstrained and constrained SSP5-8.5 scenario. Decomposition analysis clarifies that this rural decline is driven primarily by the population effect, specifically the projected decrease in rural population due to high migration and urbanization under SSP5-8.5[93], with the interaction effect compounding the reduction, while the climate effect remains positive (Supplementary Figs. S24-26b). These contrasting magnitudes and directions of change in urban and rural exposure influence total exposure. Because urban exposure is consistently positive and increases by several tens of billions of person mm day^{-1}, whereas rural exposure decreases by only a few billion, total regional averages closely follow urban trends. Even in regions where rural averages are strongly negative, such as WAF and ESAF, the magnitude of rural declines is much smaller than the increase in urban exposure. Consequently, total population exposure continues to rise under both emissions pathways. Under SSP2-4.5, Africa-wide total exposure reaches approximately 7.16×10^{10} person mm day^{-1} in the unconstrained ensemble and ~6.60×10^{10} under constraints. Under SSP5-8.5, the unconstrained total falls to 5.78×10^{10}, while the constrained mean is reduced to

roughly 4.95 × 10^{10} (Fig. 8c). These lower increases in SSP5-8.5 compared to SSP2-4.5 are due primarily to weaker overall population growth in SSP5-8.5 in Africa[93], rather than a negative change in rural exposure, which is also reflected in the population effect in urban exposure (Fig. 8a-c and Supplementary Figs. S24-S26a-c).

In contrast to population exposure, which is generally higher under SSP2-4.5, projected GDP exposure to Rx1day (Fig. 8d) is markedly greater under SSP5-8.5 across all African subregions, with model constraints similarly moderating but not reversing the direction of change. Under SSP2-4.5, Africa-wide GDP exposure reaches ~2.26 × 10^{15} USD mm day^{-1} in the unconstrained EnsMean and is reduced to ~2.45 × 10^{15} USD mm day^{-1} when constraints are applied. The amplification under SSP5-8.5 is much larger: unconstrained exposure climbs to ~5.81 × 10^{15} USD mm day^{-1} and is reduced to ~5.24 × 10^{15} USD mm day^{-1} with the constrained EnsMean. WAF dominates the continental signal, with unconstrained SSP5-8.5 exposure of ~3.18 × 10^{15} USD mm day^{-1} compared to ~2.82 × 10^{15} under constraints, while CAF, NEAF, and SEAF (MED, SAH, WSAF, and MDG) exhibit moderate (or minimal) but consistent increases. Decomposition of GDP exposure confirms that all three effects contribute positively across subregions, except for the climate and interaction effects in MED (Supplementary Figs. S24-S26d). These result in a minor negative contribution that partially offsets the net positive increase in GDP exposure from the GDP effect in MED. Overall, these patterns reflect the underlying socioeconomic assumptions of the two scenarios. SSP2-4.5 represents moderate population growth and steady economic development, whereas SSP5-8.5 assumes similar but slightly weaker population growth, with high migration and urbanization leading to rapid GDP expansion[93,94]. Consequently, higher total populations under SSP2-4.5 contribute to higher total population exposure, while accelerated economic growth under SSP5-8.5 may increase GDP exposure in densely populated or rapidly urbanizing regions." **(lines 529-587)**

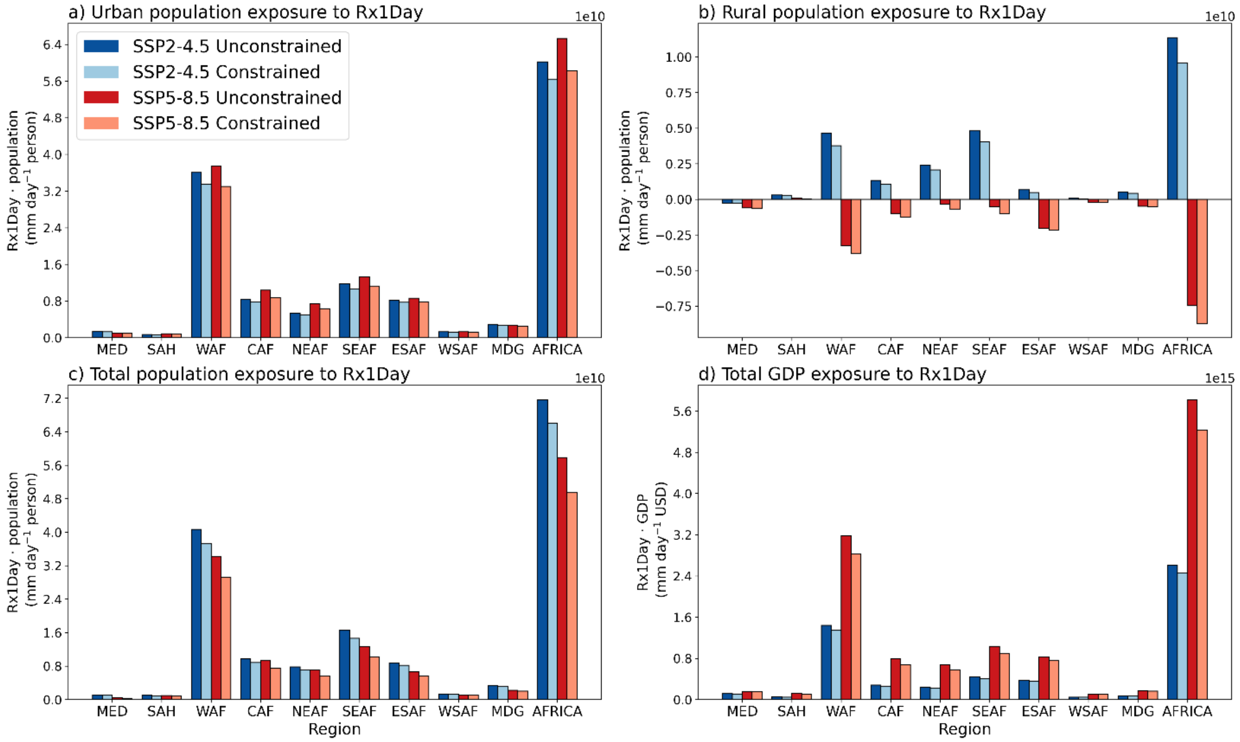

Figure 8: Population and economic exposure to extreme precipitation. Bar plots show the change in total exposure to unconstrained and constrained Rx1day for the (a) urban population, (b) rural population, (c) total population, and (d) gross domestic product (GDP) across Africa and its subregions under the SSP2-4.5 and SSP5-8.5 scenarios. Change is calculated for the future period (2070–2099) compared to the historical period (1985–2014).

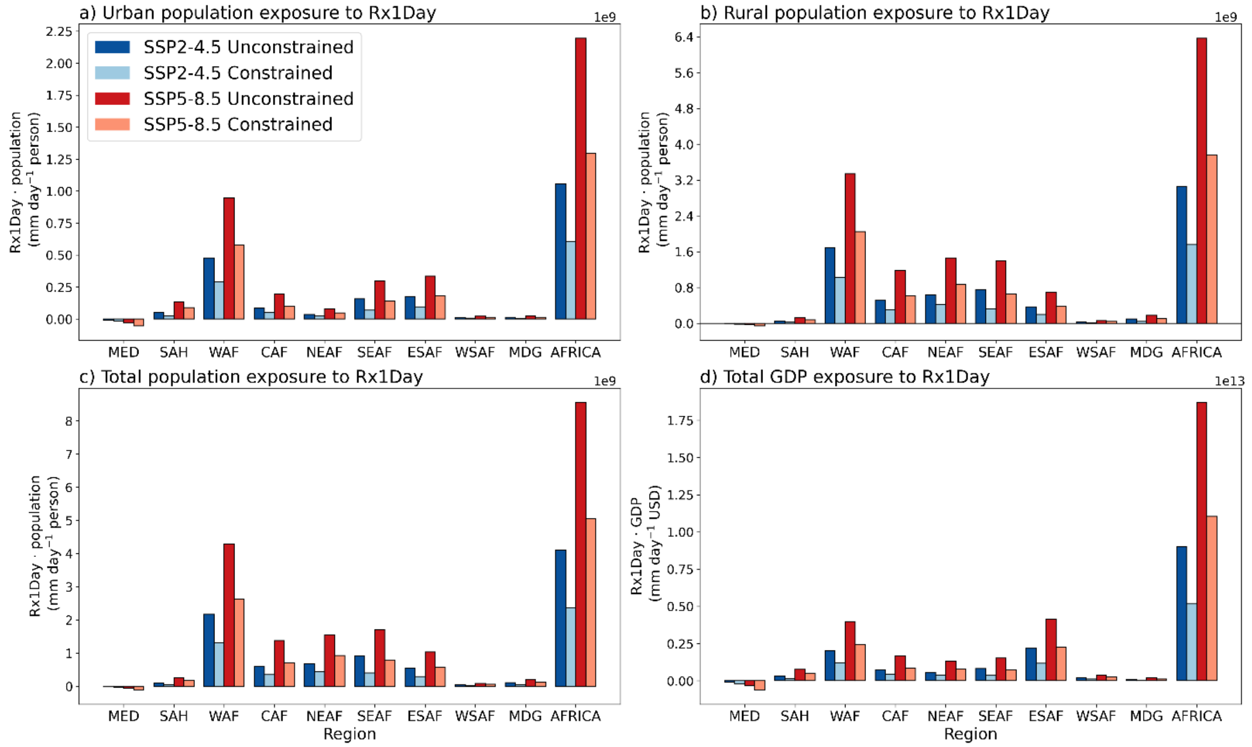

Figure S24. Bar plots show the change in the climate effect (Rx1day change) component of total exposure to unconstrained and constrained Rx1day for the (a) urban population, (b) rural population, (c) total population, and (d) gross domestic product (GDP) across Africa and its subregions under the SSP2-4.5 and SSP5-8.5 scenarios. Change is calculated for the future period (2070–2099) compared to the historical period (1985–2014).

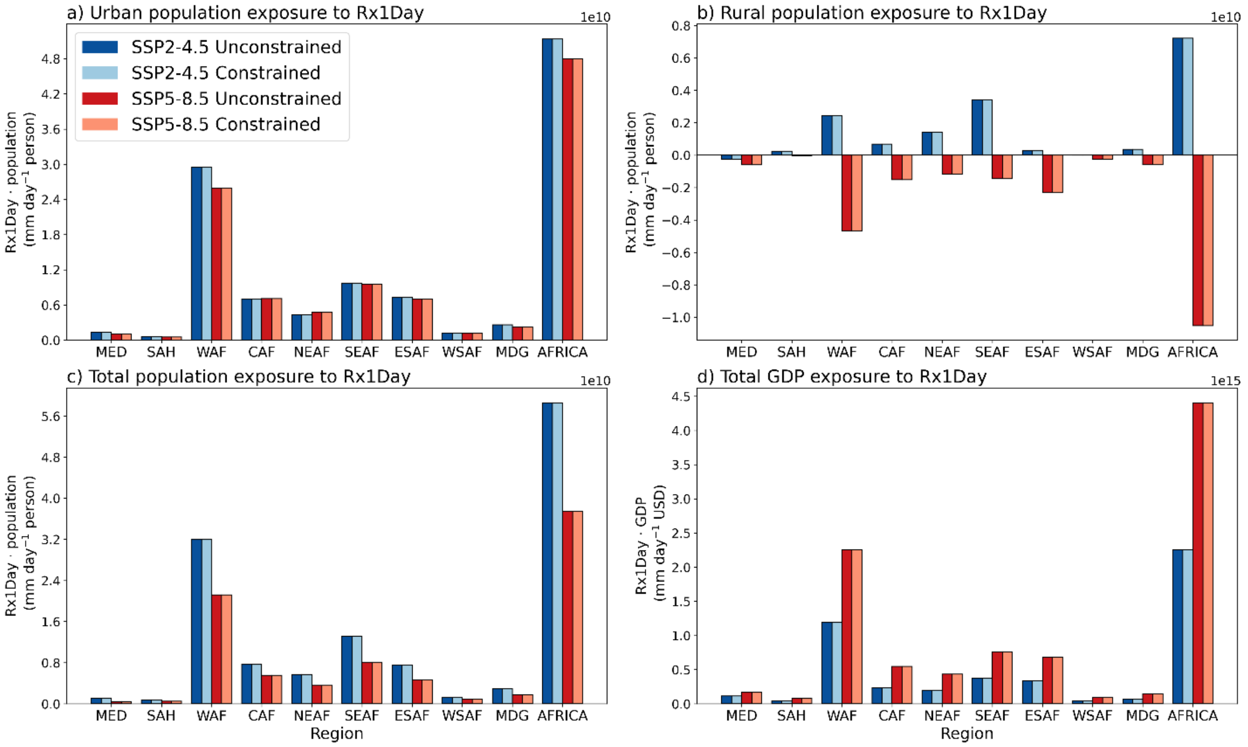

Figure S25. Bar plots show the change in the population/GDP change component of total exposure to unconstrained and constrained Rx1day for the (a) urban population, (b) rural population, (c) total population, and (d) gross domestic product (GDP) across Africa and its subregions under the SSP2-4.5 and SSP5-8.5 scenarios. Change is calculated for the future period (2070–2099) compared to the historical period (1985–2014).

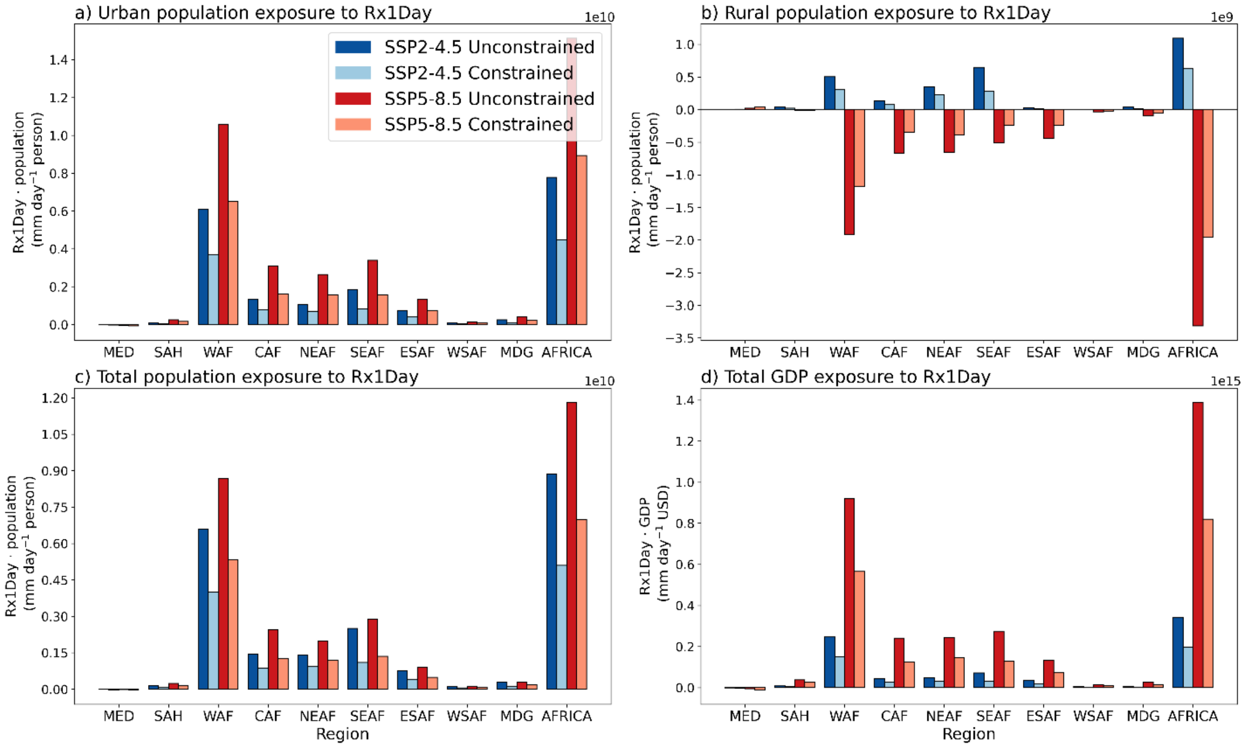

Figure S26. Bar plots show the change in the interaction component of total exposure to unconstrained and constrained Rx1day for the (a) urban population, (b) rural population, (c) total population, and (d) gross domestic product (GDP) across Africa and its subregions under the SSP2-4.5 and SSP5-8.5 scenarios. Change is calculated for the future period (2070–2099) compared to the historical period (1985–2014).

Comment: *Line 546. "subsidence-drive decreases" sounds a little odd to me since subsidence is usually linked to dry conditions. Perhaps speak about "reduced upward motion"?*

Response: We thank the reviewer for this helpful suggestion. We agree that describing the signal as reduced upward motion provides a clearer and more precise interpretation of the dynamical contribution during extreme precipitation events. We have therefore revised the sentence accordingly:

"reduced upward motion contributes to decreases in extreme precipitation in parts of subtropical southern Africa and the northern Sahara" (lines 630-632)

Comment: *Lines 549-550. Actually, the combination of Fig. 2a,c and Fig. 3b,d seems to suggest that the dynamic influence offsets the thermodynamic influence over northern Africa (including the Sahara) and western South Africa.*

Response: We thank the reviewer for this careful observation. We agree that in some regions, particularly WSAF, ESAF and MDG, the dynamical contribution can partially offset the thermodynamic amplification of precipitation extremes. We have revised the text to acknowledge this regional behavior while maintaining the broader conclusion that

thermodynamic processes dominate across most of the continent. The revised sentence now reads as:

"Although the dynamic response strengthens under SSP5-8.5, except in WSAF, ESAF, and MDG (Supplementary Fig. S1), it generally does not offset thermodynamic intensification." (lines 633-635)

Comment: *Line 559. Should "dynamical process" be "dynamical processes"?*

<u>Response</u>: Thank you for catching this. We have corrected "dynamical process" to "dynamical processes" in the revised manuscript.

Comment: *Line 560. What about a possible role of regional circulation patterns?*

<u>Response</u>: We thank the reviewer for highlighting the potential role of regional circulation patterns. We agree that uncertainties in projected precipitation extremes are not only related to the representation of subgrid-scale processes but may also reflect differences in how models simulate large-scale regional circulation systems. In the revised manuscript, we have expanded the discussion to acknowledge that variations in the representation of regional circulation features (monsoon flows, tropical convergence zones, and associated vertical motion) can also contribute to model spread in projected precipitation extremes. The revised text now reflects both sources of dynamical uncertainty.

"These contributions, which are dominated by dynamic processes, likely reflect both structural differences in how CMIP6 models represent subgrid-scale physical processes such as deep convection, cloud microphysics, and boundary-layer turbulence as well as differences in the simulation of regional circulation patterns that regulate large-scale ascent and moisture transport. These factors have remained major sources of uncertainty in tropical precipitation projections[34,95-97]." (lines 647-652)

Comment: *Line 734. Add comma between "framework, which… "?*

<u>Response</u>: Thank you for pointing this out. We have added the comma after "framework" in the revised manuscript.

Comment: *Line 766-767. Please, check the grammar of the sentences.*

<u>Response</u>: Thank you for this comment. We have revised the sentences to correct all the grammatical errors.

Comment: *Lines 760-773. It is not entirely clear to me how I should interpret the different terms of the thermodynamic energy equation in relation to processes driving changes in the dynamics of precipitation extremes. Specifically, how should a reader interpret the local temperature tendency and horizontal temperature advection terms in this context?*

Response: Thank you for this helpful comment. We have revised the discussion of Eq. (12) to briefly clarify the interpretability of each term in the thermodynamic energy budget. We have also copied the updated subsection below.

"In Equation 12, the term on the left-hand side, $\frac{\partial T}{\partial t}$, denotes the local temperature tendency. The three terms on the right-hand side describe the processes governing this tendency. The first term, $-\boldsymbol{V_h} \cdot \boldsymbol{\nabla T}$, represents horizontal temperature advection, i.e., the lateral transport of heat by the large-scale circulation. The second term, $\boldsymbol{S_p \omega}$, represents temperature changes due to vertical advection, such that upward motion ($\omega<0$) leads to adiabatic cooling and downward motion ($\omega>0$) leads to adiabatic warming, with the magnitude of the response modulated by the static stability S_p. The third term, $\frac{Q_1}{C_p}$, represents diabatic heating, i.e., net non-adiabatic heating of the column arising from processes such as latent heat release, radiation, and sensible heat fluxes." (lines 933-941)

Comment: *Lines 701-719. Perhaps consider to make the use of acronyms more consistent by replacing "DYN" by "DY"?*

Response: Thank you for catching this. We have revised the manuscript to ensure that the acronyms are used consistently throughout the manuscript.

Thank you very much for your helpful suggestions, which have helped us improve the quality of the manuscript.

Reviewer #1

<u>Comment</u>: *The authors have added important clarifications, additional analyses and better described methods and statistical assumptions. This has considerably improved the manuscript. However, there are a number of important underlying caveats to this analysis, which although acknowledged by the authors in their reviewer responses, are not explicitly stated in the manuscript. This needs to be addressed (see major comment below) but otherwise I am happy for the paper to be published.*

<u>Response</u>: We sincerely thank the reviewer for the positive assessment of our revised manuscript and for acknowledging the improvements made in response to the previous comments. We are particularly encouraged that the reviewer considers the manuscript substantially improved and suitable for publication.

We agree with the reviewer that the underlying caveats of our analysis should be more explicitly stated in the manuscript. We have now revised the Discussion section to clearly articulate these limitations. We believe that this addition improves the transparency and robustness of the manuscript, and we thank the reviewer for highlighting this important aspect.

Major comment:

<u>Comment</u>: *The authors need to be clear that the method in this study is only constraining the large-scale atmospheric moistening. This is a key driver of future changes in precipitation extremes. However, there are other factors which will determine the actual realisation of greater large-scale atmospheric moisture in terms of precipitation extremes of the future. For example, local surface conditions are important for triggering convection and local dynamical processes for its development. Although it is not possible to constrain uncertainty arising from representation of these dynamical processes using observed trends and the HEC framework here, it is possible to assess the ability of models to capture dynamical variability and feedbacks through assessment of precipitation variability across a range of space and timescales in the present climate.*

For confidence in the constrained projections, a key step forward would be to assess whether models not only represent GMST trends, but importantly also capture spatial and year-to-year variability in precipitation extremes. It maybe that all CMIP6 models are systematically underestimating changes in extremes, due to poor representation of local convective processes.

It is important that a clear statement of the caveats of this approach (namely that the constraint is based on large-scale atmospheric moisture only and the fact that only using CMIP6 models) is added to the manuscript, along with the implications for the reliability of the actual numbers quoted. Adding this statement does not undermine the value of the study – which is rather in the

combined methodology employed and understanding gained – but rather gives important insights into future research directions.

<u>Response</u>: We thank the reviewer for this insightful and constructive comment. We fully agree that our methodological framework primarily constrains the large-scale atmospheric moistening component, which is a key thermodynamic driver of future precipitation extremes. However, other important processes, such as local surface conditions, convective triggering, and dynamical feedbacks, also play a critical role in determining the realized changes in extremes.

As noted by the reviewer, an important aspect of building confidence in constrained projections is the ability of models to represent present-day characteristics of precipitation extremes. In this regard, we would like to clarify that, in the revised manuscript, we evaluate the historical spatial patterns of precipitation extremes across Africa. Overall, the models reproduce the spatial distribution reasonably well, although noticeable regional biases remain (see Figure S2). This provides some confidence in the large-scale features captured by the models, while also highlighting their limitations.

We also acknowledge the reviewer's point regarding the representation of year-to-year variability. While CMIP6 models are free-running and are not expected to reproduce the observed temporal evolution (i.e., they are not nudged with observations, so 1995 in a CMIP6 model does not correspond to 1995 in observations), they are designed to capture the statistical characteristics of internal interannual variability. However, we agree that the reliance on parameterized convection can lead to deficiencies in representing convectively driven variability and may contribute to biases in simulated precipitation extremes, as also reflected in our evaluation (Figure S2). We have therefore clarified in the manuscript that our constrained projections should be interpreted as likely conservative estimates, given these known physical limitations (lines 772–778):

"However, because convection is not explicitly resolved in CMIP6 models, the constrained projections presented in this study should be interpreted as a likely conservative estimate, or lower bound, of future intensification of precipitation extremes across Africa. Future studies combining large-ensemble convection-permitting simulations with constraint frameworks may further refine these projections as such datasets become more widely available."

In response to the reviewer's suggestion, we have further strengthened the Discussion section to explicitly state the caveat associated with the thermodynamic nature of the constraint (large-scale atmospheric moisture) and the absence of constraints on dynamical and convective processes. We have added the following text to the Discussion section:

"Nonetheless, although the HEC framework primarily constrains the thermodynamic contribution to precipitation extremes through large-scale atmospheric moistening, a dominant driver of Rx1day intensification under warming, it may also indirectly reduce

some dynamic uncertainty, because the diabatic heating that drives vertical motion depends partly on moisture availability, which is itself constrained by GMST. (lines 713-718)

We thank the reviewer again for this important suggestion, which has helped us improve the clarity and interpretation of our results.

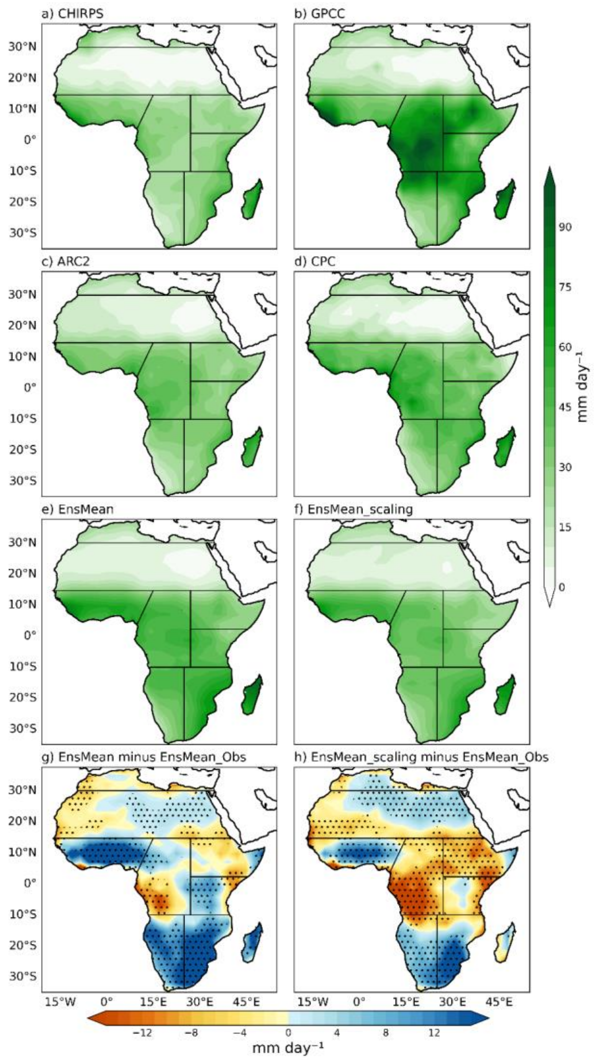

Figure S2. Mean annual maximum 1-day precipitation (Rx1day, mm/day) during the historical period (1985–2014) from (a–d) observational datasets (CHIRPS, GPCC, ARC2, and CPC), (e) the CMIP6 ensemble mean (EnsMean) RX1day, and (f) the EnsMean computed from physical-scaling diagnostics (EnsMean_scaling). Panels (g) and (h) show the bias in EnsMean Rx1day and EnsMean Rx1day_scaling, respectively, relative to the observational mean. Stippling in (g) and (h) indicates grid points where differences are statistically significant at the 95% confidence level using a Student's t-test.

Thank you very much for your helpful suggestions, which have helped us improve the quality of the manuscript.

Reviewer #2

Comment: After going carefully through the author responses to the raised comments and the revised manuscript, I feel that the revisions have adequately improved the manuscript. I have no further comments (except from a very minor one, listed below), and recommend acceptance of the manuscript in Nature Communications.

Response: We sincerely thank the reviewer for the careful evaluation of our revised manuscript and for recognizing the improvements made. We are especially grateful for the recommendation for acceptance.

We also appreciate the thoughtful and constructive feedback provided throughout the review process, which has significantly strengthened the manuscript. We address the minor comment below.

Comment: Lines 363-364. The phrase "These changes further enhance atmospheric moisture availability (Supplementary 363 Fig. S15a–b), thereby favoring more extreme precipitation events." seems not obvious from the context. Strengthened subsidence would rather hinder extreme precipitation events.

Response: We thank the reviewer for pointing out this important issue. We agree that the original phrasing could be misleading, as strengthened subsidence is typically associated with suppressed convection and would not directly favor extreme precipitation.

To address this, we have revised the sentence to better clarify the underlying mechanism and avoid potential confusion. The updated text now distinguishes between the dynamical suppression of ascent and the thermodynamic effect of increased moisture-holding capacity associated with subsidence-induced warming.

The sentence has been revised as follows:

"While subsidence dynamically suppresses ascent, the resulting warming enhances the atmospheric water-holding capacity and total column moisture (Supplementary Fig. S15a–b), thereby providing a larger moisture reservoir that favors more intense precipitation extremes when such events occur." (Lines 365-368)

We believe this revised wording more accurately reflects the physical processes involved and improves the clarity of the manuscript.

Thank you very much for your helpful suggestions, which have helped us improve the quality of the manuscript.

Reviewer #1

<u>Comment</u>: The authors have not made any substantial changes to the manuscript in response to the 2nd review. The passages of text they point to in the Discussion were previously present in the earlier version of the manuscript. Therefore my previous review comments still hold, that the underlying caveats to this analysis have not been fully acknowledged in the manuscript. It is important that the caveats of this approach (namely that the constraint is based on large-scale atmospheric moisture only and the fact that only using CMIP6 models) are fully explained in the manuscript, along with the implications for the reliability of the actual numbers quoted.

Response: We thank the reviewer for reiterating this important point. In the previous revision, the relevant caveats were present but not sufficiently explicit in terms of clearly delineating the limitations of the emergent-constraint framework. In the revised manuscript, we have now strengthened and explicitly expanded this discussion in the Discussion section (lines 736–746), ensuring that the limitations are clearly stated and directly linked to the interpretation of the constrained projections

Also copied below "A key limitation of the HEC applied here is that it primarily constrains the thermodynamic contribution to precipitation extremes through large-scale atmospheric moistening, as represented by its relationship with GMST. It does not directly constrain uncertainties associated with local surface conditions or subregional dynamic processes that are important for triggering convention and its subsequent development. In addition, the constraint is derived from the CMIP6 ensemble, in which convection is parameterized rather than explicitly resolved. As a result, systematic biases in representing convective variability and precipitation extremes may not be corrected by the constraint, and the constrained projections may therefore represent a conservative estimate of future change. Although the constraint reduces intermodel spread and improves consistency with observed large-scale warming, the constrained magnitudes of RX1day change should be interpreted with caution."
